# Synthesis of 3-(Pyridin-2-yl)quinazolin-2,4(1*H,*3*H*)-diones via Annulation of Anthranilic Esters with *N*-pyridyl Ureas †

**DOI:** 10.3390/ijms24087633

**Published:** 2023-04-21

**Authors:** Svetlana O. Baykova, Kirill K. Geyl, Sergey V. Baykov, Vadim P. Boyarskiy

**Affiliations:** Institute of Chemistry, Saint Petersburg State University, 7/9 Universitetskaya Nab., Saint Petersburg 199034, Russia; s.baykova@spbu.ru (S.O.B.); kirillgeyl@outlook.com (K.K.G.); s.baykov@spbu.ru (S.V.B.)

**Keywords:** quinazoline-2,4-diones, thienopyrimidine-2,4-diones, pyridines, quinolines, ureas, anthranilic acids derivatives, annulation

## Abstract

A new route for the synthesis of quinazolin-2,4(1*H,*3*H*)-diones and thieno [2,3-*d*]pyrimidine-2,4(1*H*,3*H*)-diones substituted by pyridyl/quinolinyl moiety in position 3 has been developed. The proposed method concluded in an annulation of substituted anthranilic esters or 2-aminothiophene-3-carboxylates with 1,1-dimethyl-3-(pyridin-2-yl) ureas. The process consists of the formation of *N*-aryl-*N*′-pyridyl ureas followed by their cyclocondensation into the corresponding fused heterocycles. The reaction does not require the use of metal catalysts and proceeds with moderate to good yields (up to 89%). The scope of the method is more than 30 examples, including compounds with both electron-withdrawing and electron-donating groups, as well as diverse functionalities. At the same time, strong electron-acceptor substituents in the pyridine ring of the starting ureas reduce the product yield or even prevent the cyclocondensation step. The reaction can be easily scaled to gram quantities.

## 1. Introduction

The quinazoline-2,4-dione fragment is part of many clinical candidates: selurampanel (an AMPA/kainate receptor antagonist for epilepsy treatment) [1,2], elinogrel (a P2Y12 receptor antagonist for the treatment of cardiovascular atherothrombotic disease) [3,4,5], zenarestat (an aldose reductase inhibitor for the management of diabetic peripheral neuropathy) [6,7], ketanserin (a 5-HT2 receptor antagonist with the hypotensive action) [8,9], carotegrast (α4 integrin antagonist for the treatment of ulcerative colitis) [10,11], senaparib (a PARP inhibitor for the therapy of solid tumors) [12,13,14], and BMS-986142 (a Bruton’s tyrosine kinase inhibitor for the treatment of rheumatoid arthritis) [15,16,17]. In addition, quinazoline-2,4-dione derivatives were recognized as inhibitors of several cancer-related enzymes [18] (including carbonic anhydrases IX and XII [19], histone deacetylase-6 [20], VEGFR-2 [21], tankyrases [22], aminopeptidase [23]) and as modulators of autoimmune processes [24,25,26,27]. Moreover, they are widely used to combat viral [28,29], bacterial [30,31,32], parasitic [33,34,35], and fungal [36] infections.

Thienopyrimidine-2,4-diones is another class of medicinally important annulated dicarbonyl heterocycles [37]. These compounds found application in the design of gonadotropin-releasing hormone receptor antagonists [38,39,40,41,42,43] and acetyl-CoA carboxylase inhibitors [44,45,46].

In agriculture, both scaffolds (quinazoline-2,4-diones and thienopyrimidine-2,4-diones) are employed for weed control [47,48].

**Scheme 1:**
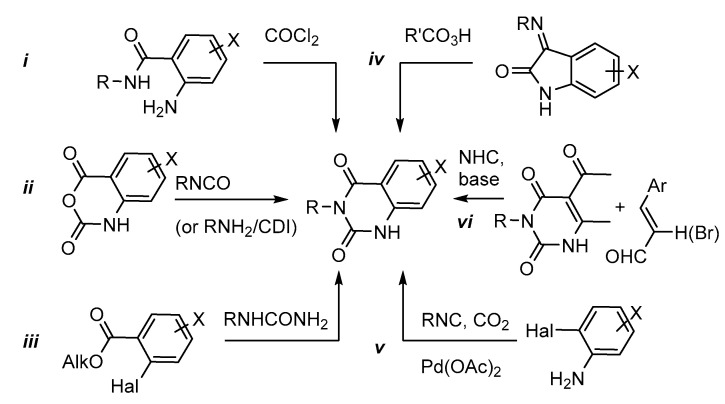
Methods for synthesis of substituted quinazolin-2,4-diones.

Among the quinazoline and thienopyrimidine diones described in the literature, most of the compounds contain a substituent in position 3. The biological significance causes the emergence of a number of methods for the synthesis of 3-substituted quinazoline-2,4-diones, including (i) the treatment of 2-aminobenzamides with phosgene, (ii) the reaction of isatoic anhydride with amines or isocyanates, (iii) the condensation of 2-halobenzoates with monoalkylureas, (iv) Baeyer–Villiger oxidation of 4-iminoisatins, (v) the three-component catalytic condensation of 2-haloanilines with CO_2_ and isocyanides (Scheme 1) [49,50]. Instead of phosgene, various phosgene surrogates can be used as a carbonylating agent, including phenylisocyanate [51] or Troc-group [52]. Recently, an alternative route for the synthesis of quinazoline-2,4-diones has been proposed, which consists of benzannulation strategy—the heteroaromatic condensed system is created by closing not the hetero-, but the carbocycle (Scheme 1, route vi) [53]. All these methods have a number of disadvantages; for example, poorly available or highly toxic reagents, harsh reaction conditions, difficulty in purifying target products, and low yields. Despite this, the synthesis of 3-substituted quinazolin-2,4-diones is still attractive due to their potential therapeutic application. Therefore, the development of convenient methods for the synthesis of new quinazolin-2,4-dione derivatives is an important task for organic and medicinal chemistry.

The access to 3-substituted thienopyrimidine-2,4-diones is another significant point that does not have a convenient and general synthetic solution. In the literature, only two methods for the preparation of these compounds are described. One of them is a nucleophilic attack of an aminothiophene derivative on an isocyanate in the presence of a catalytic amount of triethylamine in refluxing 1,4-dioxane followed by treatment of formed intermediates with NaOR in refluxing ROH [54,55,56]. The other is a reaction of aminothiophene carboxamides with 2,2,6-trimethyl-*4H*-1,3-dioxin-4-one in xylene followed by formed enamino amides’ fragmentation and cyclization [57].

Another significant goal is the introduction of the pyridyl moiety into organic molecules. In addition to being important pharmacophore themselves, this heterocycle can improve pharmacokinetic properties such as aqueous solubility and permeability through biological membranes. Therefore, it is of considerable interest to find convenient access to quinazolin-2,4-diones substituted in position 3 with pyridyl moiety. Unfortunately, a general synthetic method for obtaining a wide range of substituted 3-(pyridin-2-yl)quinazolin-2,4-diones is unknown hitherto. One of the best synthetic approaches to these compounds is a copper-catalyzed domino C–C bond cleavage of 2,3-unsubstituted indole/indolines/oxindoles through oxidation followed by insertion of 2-aminopyridines [58]. However, this method utilizes pyridine-2-amines, which are characterized by insufficient diversity and a rather high cost of commercially available compounds.

At present, our research is focused on the development of simple methods for introducing pyridin-2-yl and quinolin-2-yl fragments into organic and organometallic compounds based on the use of masked isocyanates—*N*,*N*-dialkyl-*N*′-(pyridine-2-yl)ureas [59,60,61,62,63,64]. These compounds are easily synthesized from the corresponding pyridines [65,66,67,68]. In this paper, we report a simple one-step protocol for the synthesis of 3-pyridyl-substituted quinazoline- and thienopyrimidine-2,4-diones from anthranilic or 2-aminothiophene-3-carboxylic acid esters that uses this approach. Taking into account the wide range of commercially available pyridines, the proposed method has potential in the design of pharmaceutically relevant fused heterocycles.

## 2. Results and Discussion

**Scheme 2:**
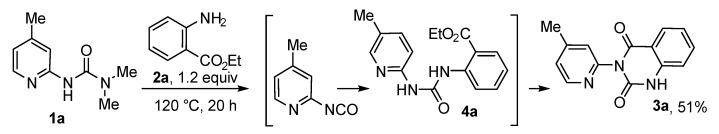
Formation of 3-(4-methylpyridin-2-yl)quinazoline-2,4(1*H*,3*H*)-dione (**3a**).

Recently, we described the reaction of *N*-pyridyl ureas with a broad spectrum of amines [59]. During this study, the reaction of 1,1-dimethyl-3-(4-methylpyridin-2-yl)urea (**1a**) with anthranilic acid ethyl ester **2a** was carried out and unexpectedly quinazoline-2,4-dione **3a** was identified as the main product (the isolated yield of 51%). The more detailed study showed that the initially formed ethyl 2-(3-(5-methylpyridin-2-yl)ureido)benzoate (**4a**) undergoes further cyclocondensation under the reaction conditions to afford quinazolin-2,4-dione **3a** (Scheme 2).

**Figure 1:**
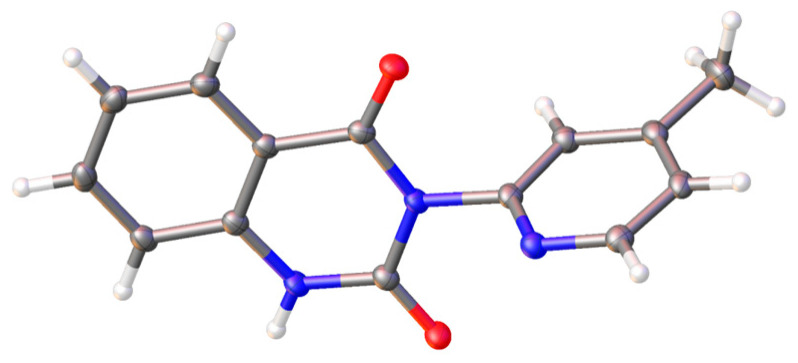
Molecular structure of quinazoline-2,4-dione **3a** (Olex2 view).

Product **3a** was characterized by high-resolution mass spectrometry and ^1^H and ^13^C NMR spectroscopies. The structure of the compound was confirmed by single-crystal X-ray diffraction (XRD, Figure 1 and Table S1).

We investigated the influence of the reaction conditions on the yield of **3a** (Table 1). We have determined that increasing the reaction time and temperature does not affect the yield (Table 1, entries 1 and 2). We enlarged the excess of anthranilic acid ethyl ester **2a** step by step in the reaction mixture and found that 5 equiv. of the one affords a higher yield (Table 1, entries 3–5). Then, we carried out the reaction under solvent-free conditions and the desired product **3a** was obtained in a better yield (Table 1, entry 6). A further increase in the **2a** excess reduced the yield of the product (Table 1, entry 7), so for the further experiments we used a 5-fold excess. Finally, we tested whether anthranilic acid could be used instead of its ester and found that the desired quinazoline-2,4-dione **3a** was formed, albeit in a lower yield (Table 1, entry 8).

**Scheme 3:**
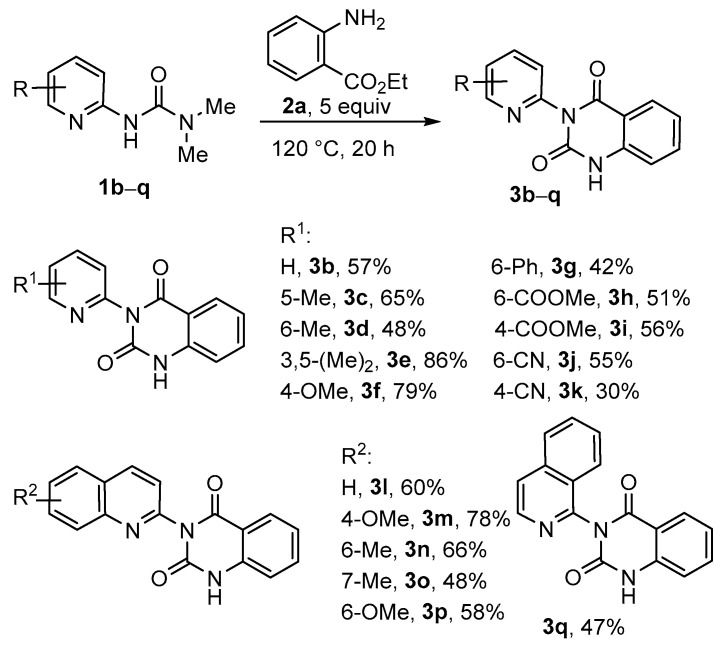
Synthesis of quinazolin-2,4-diones substituted in the pyridine moiety.

After finding the optimal reaction conditions, we determined the range of possible substrates that can participate in the process. First, we studied various substituted (pyridin-2-yl)ureas **1** and showed that the reaction proceeds in all cases, but the reaction yields vary. This indicates the sensitivity of the reaction to electronic effects of substituents in the pyridine ring (Scheme 3), but the nature of this influence is ambiguous. It can be said that electron-donating groups in positions 4 and 5 of the pyridine ring have a positive effect on the yields of quinazoline-2,4-diones **3**. The presence of two methyl groups at positions 3 and 5 of the pyridine ring (*ortho* and *para* with respect to the nitrogen atom of the ureide fragment entering into the cyclocondensation) provides the target product **3e** with the highest yield (86%). It should be noted that quinazoline-2,4-dione **3c** bearing 5-methylpyridyl moiety was obtained in a slightly lower yield (48%).

**Scheme 4:**
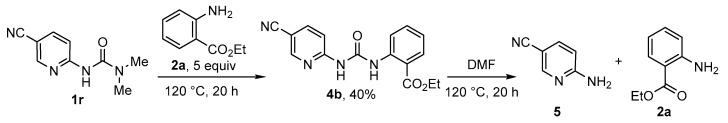
Formation and destruction of ethyl 2-(3-(5-cyanopyridin-2-yl)ureido)benzoate **4b** under the reaction conditions.

The presence of electron-withdrawing groups in the heterocyclic ring also decreased the yield of products **3**. Perhaps this is due to such substituents adversely affecting the cyclocondensation step. Particularly, in the case of the substrate containing a nitro-group at position 4 of the pyridine ring, the desired quinazoline-2,4-dione was formed in only 19% yield (according to ^1^H NMR data for the reaction mixture), whereas the main product was the corresponding intermediate urea. Moreover, the presence of the electron-withdrawing cyano-group in position 5 (*para* with respect to the nitrogen atom of the ureide fragment) completely suppressed the cyclocondensation. In this case, urea **4b** was isolated in 40% yield as the main product. The prolonged heating of **4b** at 120 °C only led to its degradation to the 2-aminopyridine **5** and the starting anthranilic acid ethyl ester **2a** (Scheme 4).

Substituents in position 6 of the starting *N*–pyridylureas **1** slightly reduced the yield of target quinazolin-2,4-diones **3** (Scheme 3). The reason for this is the steric hindrances, but their effect is not very significant. Therefore, using ureas bearing quinoline and isoquinoline moieties **1l–q** as starting compounds, we synthesized 6 corresponding quinazolin-2,4-diones (**3l**–**q**) in moderate to good yields (47–78%, Scheme 3).

**Scheme 5:**
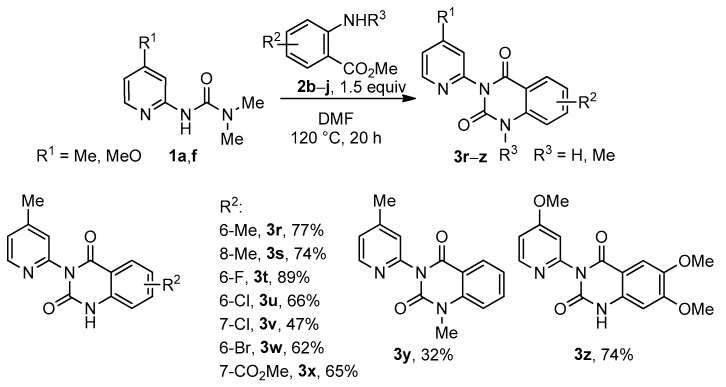
Investigation of the reactivity of various anthranilic esters.

Next, we checked the possibility of synthesizing quinazolin-2,4-diones bearing substituents in the quinazoline fragment via the developed procedure. For this goal, the scope of functionalized anthranilic esters was investigated. Reactions were carried out with 1.5 equiv. of esters **2b**–**j** in DMF at 120 °C. We found that neither electron-donating nor electron-withdrawing substituents prevented the reaction and the corresponding quinazolin-2,4-diones (**3r**–**x,z)** were successfully obtained in 47–89% yields (Scheme 5). In addition, this method allows us to obtain *N1*-alkylsubstituted quinazolin-2,4-diones, however, with less yield. Particularly, the reaction between urea **1a** and *N*-methyl anthranilic ester **2h** provided target product **3y** with 32% yield only. Presumably, such poor yield of **3y** is caused by instability of *N*-alkyl-*N*-aryl urea (the proposed intermediate) in the reaction conditions and its side transformation into 1,3-bis(4-methylpyridin-2-yl)urea, which was also detected in the reaction mixture. This process was described in our previous work [59].

**Scheme 6:**
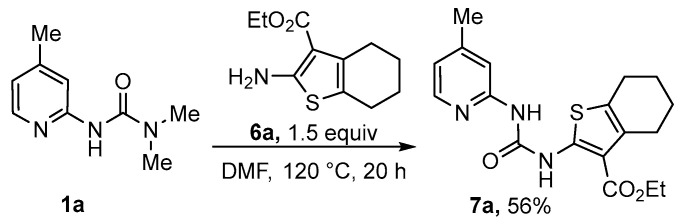
Coupling of ureas **1a** with 2-aminothiophene-3-carboxylates **6a**.

To expand the reaction scope, we studied the reactivity 2-aminothiophene-3-carboxylates (products of the Gewald reaction) in this process. It turned out that when ethyl 2-amino-4,5,6,7-tetrahydrobenzo[*b*]thiophene-3-carboxylate (**6a**) was used as a starting amine, the reaction stopped at the stage of the urea **7a** formation and any traces of a desired thienopyrimidine-2,4-dione were not observed in the reaction mixture (Scheme 6).

**Scheme 7:**
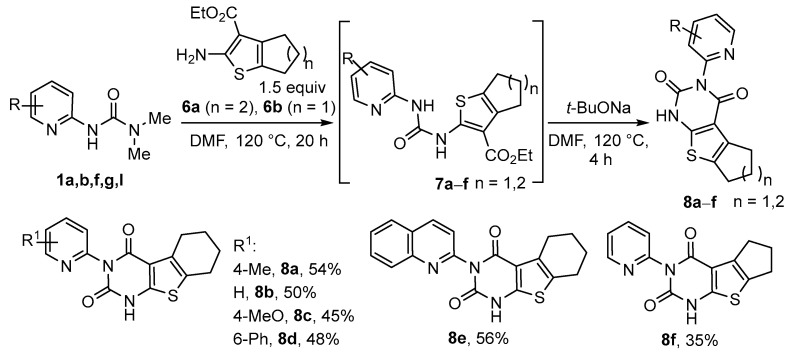
Two-stage one-pot synthesis of thienopyrimidine-2,4-diones **8a**–**f**.

Then, the conditions search for the implementation of the cyclocondensation was performed. Since the occurrence of similar reactions under basic conditions is described in the literature [50,69], we tested several bases and *t*-BuONa gave the best results. Having chosen the conditions for the cyclocondensation of the intermediate urea **7**, we synthesized thienopyrimidine-2,4-diones **8a**–**f** in the overall yield of 35–56% according to the two-stage one-pot procedure (Scheme 7).

**Scheme 8:**
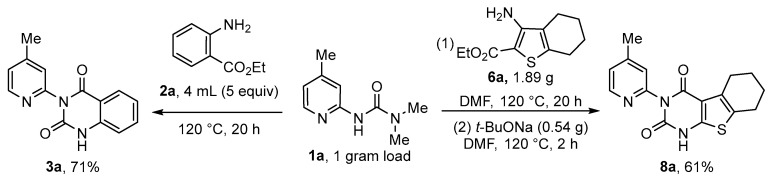
Gram-scale syntheses of **3a** and **8a**. To achieve these yields, a 100 mL flask has to be used.

Finally, to highlight the practicality of this method, the scale-up syntheses of quinazoline-2,4-dione **3a** and thienopyrimidine-2,4-dione **8a** were performed (Scheme 8).

## 3. Material and Methods

### 3.1. General

The starting *N*-oxides, used to obtain the *N*-piridyl ureas, were synthesized according to the literature procedures [65,70,71,72]. All other reagents and solvents were purchased and were used as is. Column chromatography was carried out with silica gel grade 60 (0.040–0.063 mm) 230–400. NMR spectra were recorded on Bruker Avance DPX 400 (400 MHz, 101 MHz, and 376 MHz for ^1^H, ^13^C, and ^19^F, respectively) in DMSO–*d*_6_ or CDCl_3_. Chemical shifts are reported as parts per million (*δ*, ppm). The ^1^H and ^13^C spectra were calibrated using the residual signals of nondeuterated solvents as internal reference (2.50 ppm for residual ^1^H and 39.50 ppm for ^13^C in DMSO–*d*_6_, 7.26 ppm for residual ^1^H and 77.16 ppm for ^13^C in CDCl_3_). ^19^F NMR spectra were referenced through the solvent lock (2H) signal according to IUPAC recommended secondary referencing method and the manufacturer’s protocols and the chemical shifts are reported relative to CFCl_3_ (δ 0.0 ppm). Multiplicities are abbreviated as follows: s = singlet, d = doublet, t = triplet, q = quartet, m = multiplet, br = broad; coupling constants, *J*, are reported in Hertz (Hz). Melting points were determined in open capillary tubes on Electrothermal IA 9300 series digital melting point apparatus. High–resolution mass spectra (HRMS) were measured on Bruker Maxis HRMS–ESI–qTOF (ESI ionization).

Singe crystal for X-ray studying was obtained by slow evaporation of DMSO solution of quinazoline-2,4,-dione **3a** at RT in air. X-ray diffraction data were collected via Rigaku XtaLAB Synergy–S diffractometer using CuKα (λ = 0.154184 nm) radiation. The structure was solved with the ShelXT [73] structure solution program using intrinsic phasing and refined with the ShelXL [74] refinement program incorporated in the OLEX2 program package [75] using least squares minimization. Empirical absorption correction was applied in the CrysAlisPro [76] program complex using spherical harmonics, implemented in SCALE3 ABSPACK scaling algorithm. Supplementary crystallographic data for this paper have been deposited at Cambridge Crystallographic Data Centre and can be obtained free of charge via www.ccdc.cam.ac.uk/data_request/cif (CCDC number 2249286) (accessed on 28 March 2023).

### 3.2. Preparation of Starting Ureas ***1a–r***

Ureas **1a–d**,**f**,**i**–**k**,**r** [65], **1e,g,m** [59], **1l**,**q** [61] were prepared according to the previously reported protocols. Ureas **1h,n–p** were synthesized and characterized for the first time.

**Synthesis of methyl 6-(3,3-dimethylureido)picolinate 1h**. A mixture of *N*-oxide (1 mmol), dimethylcyanamide (1.5 mmol), and acetonitrile (2 mL, 20 mmol) was stirred at RT for 2 min, and then methanesulfonic acid (1.5 mmol) was added dropwise over 3 min. Then, the reaction mixture was gently heated to 60 °C and stirred for 3 h, cooled to RT, diluted with a saturated aq. Na_2_CO_3_ (2 mL) and aq. NaCl solution (5 mL) and extracted with ethyl acetate (4×15 mL). Combined organic fractions were dried over anhydrous Na_2_SO_4_, filtered, and concentrated in a rotary evaporator. The crude product was subjected to column chromatography on silica gel (EtOAc/hexane) to give target urea **1h** in 40% yield (90 mg) as a light-yellow powder; mp 74–75 °C. ^1^H NMR (400 MHz, CDCl_3_) *δ* 8.30 (dd, *J* = 7.7, 1.5 Hz, 1H), 7.74–7.82 (m, 2H), 7.48 (s, 1H), 3.98 (s, 3H), 3.07 (s, 6H). ^13^C NMR (101 MHz, CDCl_3_) *δ* 165.5, 154.9, 153.2, 145.5, 139.0, 119.8, 117.3, 52.9, 36.6 (2C). HRMS (ESI), m/z: [M + Na]^+^ calcd. for C_10_H_13_N_3_O_3_ 246.0849; found 246.0849.

**Synthesis of ureas 1n–p**. A mixture of substituted quinoline *N*-oxide (1 mmol), dimethylcyanamide (2 mmol), and acetonitrile (0.5 mL, 5 mmol) was stirred at RT for 2 min, and then methanesulfonic acid (1.1 mmol) was added dropwise over 3 min. Then, the reaction mixture was gently heated to 60 °C and stirred for 2 h, cooled, and diluted with a saturated aq. Na_2_CO_3_ (2 mL) and distilled water (5 mL). The precipitate formed was filtered off, washed with diethyl ether (10 mL) to give compounds **1n–p**.

**1,1-Dimethyl-3-(6-methylquinolin-2-yl)urea 1n**. Beige powder; 47 yield (108 mg); mp 140–141 °C. ^1^H NMR (400 MHz, CDCl_3_) *δ* 8.27 (d, *J* = 9.0 Hz, 1H), 8.03 (d, *J* = 9.0 Hz, 1H), 7.67 (d, *J* = 8.6 Hz, 1H), 7.53 (s, 1H), 7.47 (d, *J* = 8.6 Hz, 1H), 7.41 (s, 1H), 3.11 (s, 6H), 2.51 (s, 3H). ^13^C NMR (101 MHz, CDCl_3_) *δ* 155.1, 152.0, 145.2, 137.6, 134.2, 132.0, 126.7 (2C), 125.9, 114.2, 36.6 (2C), 21.4. HRMS (ESI), m/z: [M + H]^+^ calcd. for C_13_H_15_N_3_O 230.1287; found 230.1290.

**1,1-Dimethyl-3-(7-methylquinolin-2-yl)urea 1o**. Beige powder; 51% yield (117 mg); mp 101–103 °C. ^1^H NMR (400 MHz, CDCl_3_) *δ* 8.23 (s, 1H), 8.05 (s, 1H), 7.80–7.42 (m, 3H), 7.22 (d, *J* = 8.2 Hz, 1H), 3.09 (s, 6H), 2.51 (s, 3H). ^13^C NMR (101 MHz, CDCl_3_) *δ* 155.1, 152.7, 147.0, 140.8, 140.2, 138.0, 127.3, 126.7, 126.1, 113.3, 36.6 (2C), 22.0. HRMS (ESI), m/z: [M + H]^+^ calcd. for C_13_H_15_N_3_O 230.1288; found 230.1290.

**3-(6-Methoxyquinolin-2-yl)-1,1-dimethylurea 1p**. Beige powder; 99% yield (242 mg); mp 56–58 °C. ^1^H NMR (400 MHz, CDCl_3_) δ 8.27 (d, *J* = 9.0 Hz, 1H), 8.03 (d, *J* = 9.1 Hz, 1H), 7.69 (d, *J* = 9.2 Hz, 1H), 7.51 (br s, 1H), 7.30 (dd, *J* = 9.2, 2.9 Hz, 1H), 7.06 (d, *J* = 2.8 Hz, 1H), 3.92 (s, 3H), 3.11 (s, 6H). ^13^C NMR (101 MHz, CDCl_3_) δ 156.5, 155.3, 150.9, 142.3, 137.2, 128.2, 126.5, 122.1, 114.6, 105.8, 55.6, 36.6 (2C). HRMS (ESI), m/z: [M + H]^+^ calcd. for C_13_H_15_N_3_O_2_ 246.1237; found 246.1226.

### 3.3. Synthesis of Quinazoline-2,4-Diones ***3***

*General procedure A*. Urea **1** (0.2 mmol) and ethyl anthranilate **2** (1 mmol) were placed in a vial and the resulting mixture was stirred at 120 °C for 20 h. The reaction mixture was cooled to RT, treated with diethyl ether (5 mL), and the precipitate was separated, then the precipitate was washed with diethyl ether to give compounds **3a–q**.

*General procedure B*. Urea **1** (0.2 mmol), substituted ethyl anthranilate **2** (1 mmol), and DMF (0.1 mL) were placed in a vial and the resulting mixture was stirred at 120 °C for 20 h. The reaction mixture was cooled to RT, DMF was removed by a rotary evaporator, and the residue was treated with diethyl ether (5 mL). The resulting precipitate was separated and washed with diethyl ether to give compounds **3r–z**.

**3-(4-Methylpyridin-2-yl)quinazoline-2,4(1*H*,3*H*)-dione 3a** [58]. White powder; 70% yield (35 mg); mp 266–268 °C. ^1^H NMR (400 MHz, DMSO-*d*_6_) *δ* 11.63 (s, 1H), 8.51–8.41 (m, 1H), 7.99–7.89 (m, 1H), 7.78–7.67 (m, 1H), 7.39–7.31 (m, 2H), 7.31–7.20 (m, 2H), 2.39 (s, 3H). ^13^C NMR (101 MHz, DMSO-*d*_6_) *δ* 162.1, 149.9, 149.7, 149.2, 148.9, 139.9, 135.5, 127.5, 125.0, 124.8, 122.8, 115.4, 114.1, 20.3. HRMS (ESI), m/z: [M + Na]^+^ calcd. for C_14_H_11_N_3_O_2_ 276.0743; found 276.0750.

**3-(Pyridin-2-yl)quinazoline-2,4(1*H*,3*H*)-dione 3b** [58]. White powder; 57% yield (27 mg); mp 266–267 °C. ^1^H NMR (400 MHz, DMSO-*d*_6_) *δ* 11.63 (s, 1H), 8.63–8.58 (m, 1H), 8.00 (td, *J* = 7.7, 1.9 Hz, 1H), 7.95 (dd, *J* = 8.3, 1.6 Hz, 1H), 7.76–7.70 (m, 1H), 7.54–7.48 (m, 2H), 7.28–7.22 (m, 2H). ^13^C NMR (101 MHz, DMSO-*d*_6_) *δ* 162.1, 149.9, 149.3, 149.3, 139.9, 138.7, 135.5, 127.5, 124.4, 124.1, 122.7, 115.4, 114.5. HRMS (ESI), m/z: [M + Na]^+^ calcd. for C_13_H_9_N_3_O_2_ 262.0587; found 262.0587.

**3-(5-Methylpyridin-2-yl)quinazoline-2,4(1*H*,3*H*)-dione 3c** [58]. White powder; 65% yield (33 mg); mp 230–232 °C. ^1^H NMR (400 MHz, DMSO-*d*_6_) *δ* 11.59 (s, 1H), 8.42 (d, *J* = 2.3 Hz, 1H), 7.97–7.91 (m, 1H), 7.80 (dd, *J* = 8.1, 2.4 Hz, 1H), 7.75–7.68 (m, 1H), 7.38 (d, *J* = 8.0 Hz, 1H), 7.27–7.20 (m, 2H), 2.38 (s, 3H). ^13^C NMR (101 MHz, DMSO-*d*_6_) *δ* 162.1, 150.0, 149.3, 146.8, 139.9, 138.9, 135.4, 133.6, 127.5, 123.6, 122.7, 115.4, 114.2, 17.5. HRMS (ESI), m/z: [M + Na]^+^ calcd. for C_14_H_11_N_3_O_2_ 276.0743; found 276.0745.

**3-(6-Methylpyridin-2-yl)quinazoline-2,4(1*H*,3*H*)-dione 3d** [58]. White powder; 48% yield (24 mg); mp 285–287 °C. ^1^H NMR (400 MHz, DMSO-*d*_6_) *δ* 11.60 (s, 1H), 7.94 (dd, *J* = 8.3, 1.6 Hz, 1H), 7.87 (t, *J* = 7.7 Hz, 1H), 7.75–7.68 (m, 1H), 7.36 (d, *J* = 7.6 Hz, 1H), 7.29 (d, *J* = 7.8 Hz, 1H), 7.27–7.21 (m, 2H), 2.49 (s, 3H). ^13^C NMR (101 MHz, DMSO-*d*_6_) *δ* 162.1, 158.1, 149.9, 148.5, 139.9, 138.8, 135.4, 127.4, 123.4, 122.7, 121.3, 115.4, 114.2, 23.6. HRMS (ESI), m/z: [M + Na]^+^ calcd. for C_14_H_11_N_3_O_2_ 276.0743; found 276.0747.

**3-(3,5-Dimethylpyridin-2-yl)quinazoline-2,4(1*H*,3*H*)-dione 3e**. White powder; 86% yield (46 mg); mp 261–263 °C. ^1^H NMR (400 MHz, DMSO-*d*_6_) *δ* 11.66 (s, 1H), 8.24 (d, *J* = 1.5 Hz, 1H), 7.96 (dd, *J* = 8.0, 1.5 Hz, 1H), 7.77–7.70 (m, 1H), 7.69–7.64 (m, 1H), 7.31–7.23 (m, 2H), 2.35 (s, 3H), 2.08 (s, 3H). ^13^C NMR (101 MHz, DMSO-*d*_6_) *δ* 161.7, 149.4, 147.0, 145.8, 140.0 (2C), 135.6, 133.9, 131.0, 127.5, 122.9, 115.5, 113.9, 17.3, 16.2. HRMS (ESI), m/z: [M + Na]^+^ calcd. for C_15_H_13_N_3_O_2_ 290.0900; found 290.0905.

**3-(4-Methoxypyridin-2-yl)quinazoline-2,4(1*H*,3*H*)-dione 3f**. White powder; 79% yield (42 mg); mp 240–242 °C. ^1^H NMR (400 MHz, DMSO-*d*_6_) *δ* 11.60 (s, 1H), 8.40 (d, *J* = 5.8 Hz, 1H), 7.94 (dd, *J* = 8.1, 1.6 Hz, 1H), 7.76–7.69 (m, 1H), 7.28–7.22 (m, 2H), 7.15 (d, *J* = 2.4 Hz, 1H), 7.09 (dd, *J* = 5.8, 2.5 Hz, 1H), 3.86 (s, 3H). ^13^C NMR (101 MHz, DMSO) *δ* 167.1, 162.0, 150.8, 150.1, 149.8, 139.9, 135.5, 127.5, 122.7, 115.4, 114.2, 110.5, 110.4, 55.8. HRMS (ESI), m/z: [M + H]^+^ calcd. for C_14_H_11_N_3_O_3_ 270.0873; found 270.0877.

**3-(6-Phenylpyridin-2-yl)quinazoline-2,4(1*H*,3*H*)-dione 3g**. White powder; 42% yield (26 mg); mp 279–280 °C. ^1^H NMR (400 MHz, DMSO-*d*_6_) *δ* 11.66 (s, 1H), 8.13–8.01 (m, 4H), 7.97 (dd, *J* = 8.0, 1.5 Hz, 1H), 7.78–7.71 (m, 1H), 7.54–7.44 (m, 4H), 7.31–7.23 (m, 2H). ^13^C NMR (101 MHz, DMSO-*d*_6_) *δ* 162.1, 156.3, 149.9, 149.2, 140.0, 139.8, 137.7, 135.5, 129.4, 128.8, 127.5, 126.7, 123.0, 122.8, 120.4, 115.5, 114.2. HRMS (ESI), m/z: [M + Na]^+^ calcd. for C_19_H_13_N_3_O_2_ 338.0900; found 338.0902.

**Methyl 6-(2,4-dioxo-1,4-dihydroquinazolin-3(2*H*)-yl)picolinate 3h**. Light beige powder; 51% yield (30 mg); mp 247–249 °C. ^1^H NMR (400 MHz, DMSO-*d*_6_) *δ* 11.72 (s, 1H), 8.26–8.16 (m, 2H), 7.99–7.93 (m, 1H), 7.82 (dd, *J* = 7.5, 1.3 Hz, 1H), 7.78–7.72 (m, 1H), 7.32–7.24 (m, 2H), 3.90 (s, 3H). ^13^C NMR (101 MHz, DMSO-*d*_6_) *δ* 164.5, 162.1, 149.8, 149.3, 147.3, 140.3, 140.0, 135.6, 128.4, 127.5, 125.2, 122.9, 115.5, 114.1, 52.6. HRMS (ESI), m/z: [M + Na]^+^ calcd. for C_15_H_11_N_3_O_4_ 320.0642; found 320.0638.

**Methyl 2-(2,4-dioxo-1,4-dihydroquinazolin-3(2*H*)-yl)isonicotinate 3i**. Beige powder; 56% yield (33 mg); mp 233–235 °C. ^1^H NMR (400 MHz, DMSO-*d*_6_) *δ* 11.67 (s, 1H), 8.83 (dd, *J* = 5.0, 0.8 Hz, 1H), 8.10–8.01 (m, 1H), 7.99–7.92 (m, 2H), 7.77–7.70 (m, 1H), 7.29–7.23 (m, 2H), 3.93 (s, 3H). ^13^C NMR (101 MHz, DMSO-*d*_6_) *δ* 164.5, 162.2, 150.6, 150.5, 149.9, 140.0, 139.5, 135.5, 127.5, 123.7, 123.0, 122.8, 115.5, 114.2, 53.0. HRMS (ESI), m/z: [M + H]^+^ calcd. for C_15_H_11_N_3_O_4_ 298.0822; found 298.0836.

**6-(2,4-Dioxo-1,4-dihydroquinazolin-3(2*H*)-yl)picolinonitrile 3j**. Light beige powder; 55% yield (29 mg); mp 295–297 °C (dec.). ^1^H NMR (400 MHz, DMSO-*d*_6_) *δ* 11.72 (s, 1H), 8.31 (t, *J* = 7.8 Hz, 1H), 8.20 (d, *J* = 7.6 Hz, 1H), 8.01–7.90 (m, 2H), 7.79–7.72 (m, 1H), 7.32–7.22 (m, 2H). ^13^C NMR (101 MHz, DMSO-*d*_6_) *δ* 162.0, 150.4, 149.6, 141.0, 140.0, 135.7, 132.0, 129.6, 129.4, 127.5, 122.9, 116.7, 115.6, 114.0. HRMS (ESI), m/z: [M + Na]^+^ calcd. for C_14_H_8_N_4_O_2_ 287.0539; found 287.0541.

**2-(2,4-Dioxo-1,4-dihydroquinazolin-3(2*H*)-yl)isonicotinonitrile 3k**. White powder; 30% yield (16 mg); mp 306–308 °C (dec.). ^1^H NMR (400 MHz, DMSO-*d*_6_) *δ* 11.77 (s, 1H), 8.90 (d, *J* = 5.1 Hz, 1H), 8.16–8.09 (m, 1H), 8.03 (dd, *J* = 5.0, 1.5 Hz, 1H), 7.96 (dd, *J* = 8.0, 1.5 Hz, 1H), 7.79–7.73 (m, 1H), 7.31–7.24 (m, 2H). ^13^C NMR (101 MHz, DMSO-*d*_6_) *δ* 162.0, 151.0, 150.2, 149.6, 139.9, 135.8, 127.5, 126.7, 126.2, 123.0, 121.6, 116.1, 115.6, 114.0. HRMS (ESI), m/z: [M + Na]^+^ calcd. for C_14_H_8_N_4_O_2_ 287.0539; found 287.0536.

**3-(Quinolin-2-yl)quinazoline-2,4(1*H*,3*H*)-dione 3l**. White powder; 60% yield (35 mg); mp 256–259 °C. ^1^H NMR (400 MHz, DMSO-*d*_6_) δ 11.71 (s, 1H), 8.57 (d, *J* = 8.5 Hz, 1H), 8.11 (d, *J* = 8.1 Hz, 1H), 8.03 (d, *J* = 8.4 Hz, 1H), 7.98 (d, *J* = 8.0 Hz, 1H), 7.87–7.82 (m, 1H), 7.78–7.70 (m, 2H), 7.66 (d, *J* = 8.5 Hz, 1H), 7.32–7.25 (m, 2H). ^13^C NMR (101 MHz, DMSO-*d*_6_) δ 162.3, 150.0, 149.0, 146.9, 140.0, 138.8, 135.5, 130.1, 128.6, 127.9, 127.6, 127.5 (2C), 122.8, 122.1, 115.5, 114.3. HRMS (ESI), m/z: [M + Na]^+^ calcd. for C_17_H_11_N_3_O_2_ 312.0743; found 312.0747.

**3-(4-Methoxyquinolin-2-yl)quinazoline-2,4(1*H*,3*H*)-dione 3m**. White powder; 78% yield (50 mg); mp 284–285 °C. ^1^H NMR (400 MHz, DMSO-*d*_6_) δ 11.69 (s, 1H), 8.21 (dd, *J* = 8.5, 1.5 Hz, 1H), 8.00–7.93 (m, 2H), 7.85–7.79 (m, 1H), 7.78–7.72 (m, 1H), 7.70–7.64 (m, 1H), 7.33–7.22 (m, 3H), 4.05 (s, 3H). ^13^C NMR (101 MHz, DMSO-*d*_6_) δ 163.5, 162.1, 150.4, 149.9, 147.4, 140.0, 135.5, 130.4, 128.4, 127.4, 126.6, 122.8, 121.6, 120.4, 115.5, 114.2, 101.7, 56.6. HRMS (ESI), m/z: [M + H]^+^ calcd. for C_18_H_13_N_3_O_3_ 320.1030; found 320.1033.

**3-(6-Methylquinolin-2-yl)quinazoline-2,4(1*H*,3*H*)-dione 3n**. White powder; 66% yield (40 mg); mp 310–311 °C. ^1^H NMR (400 MHz, DMSO-*d*_6_) *δ* 11.69 (s, 1H), 8.45 (d, *J* = 8.5 Hz, 1H), 8.09–7.88 (m, 2H), 7.87 (s, 1H), 7.74 (t, *J* = 7.8 Hz, 1H), 7.68 (d, *J* = 8.6 Hz, 1H), 7.59 (d, *J* = 8.5 Hz, 1H), 7.40–7.14 (m, 2H), 2.56 (s, 3H). ^13^C NMR (101 MHz, DMSO-*d*_6_) *δ* 162.3, 150.0, 148.2, 145.5, 140.0, 138.0, 137.0, 135.5, 132.2, 128.3, 127.7, 127.5, 126.6, 122.8, 122.1, 115.5, 114.3, 21.2. HRMS (ESI), m/z: [M + H]^+^ calcd. for C_18_H_13_N_3_O_2_ 304.1081; found 304.1089.

**3-(7-Methylquinolin-2-yl)quinazoline-2,4(1*H*,3*H*)-dione 3o**. White powder; 48% yield (29 mg); mp 279–281 °C. ^1^H NMR (400 MHz, DMSO-*d*_6_) *δ* 11.68 (s, 1H), 8.49 (d, *J* = 8.5 Hz, 1H), 7.98 (t, *J* = 8.5 Hz, 2H), 7.81 (s, 1H), 7.78–7.71 (m, 1H), 7.56 (d, *J* = 8.4 Hz, 2H), 7.34–7.22 (m, 2H), 2.56 (s, 3H). ^13^C NMR (101 MHz, DMSO-*d*_6_) *δ* 162.3, 150.0, 149.0, 147.2, 140.1, 140.0, 138.4, 135.5, 129.6, 127.6, 127.5, 127.4, 125.7, 122.8, 121.2, 115.5, 114.3, 21.4. HRMS (ESI), m/z: [M + H]^+^ calcd. for C_18_H_13_N_3_O_2_ 304.1081; found 304.1078.

**3-(6-Methoxyquinolin-2-yl)quinazoline-2,4(1*H*,3*H*)-dione 3p**. White powder; 58% yield (37 mg); mp 303–305 °C. ^1^H NMR (400 MHz, DMSO-*d*_6_) *δ* 11.68 (s, 1H), 8.43 (d, *J* = 8.5 Hz, 1H), 7.97 (dd, *J* = 8.0, 1.5 Hz, 1H), 7.92 (d, *J* = 8.9 Hz, 1H), 7.79–7.71 (m, 1H), 7.58 (d, *J* = 8.5 Hz, 1H), 7.53–7.44 (m, 2H), 7.32–7.22 (m, 2H), 3.94 (s, 3H). ^13^C NMR (101 MHz, DMSO-*d*_6_) *δ* 162.3, 157.9, 150.1, 146.7, 142.8, 140.0, 137.5, 135.5, 130.0, 128.9, 127.5, 122.7, 122.5, 122.3, 115.5, 114.3, 105.8, 55.6. HRMS (ESI), m/z: [M + H]^+^ calcd. for C_18_H_13_N_3_O_3_ 320.1030; found 320.1029.

**3-(Isoquinolin-1-yl)quinazoline-2,4(1*H*,3*H*)-dione 3q**. Light beige powder; 47% yield (27 mg); mp 269–271 °C. ^1^H NMR (400 MHz, DMSO-*d*_6_) *δ* 11.80 (s, 1H), 8.53 (d, *J* = 5.6 Hz, 1H), 8.12 (d, *J* = 8.3 Hz, 1H), 8.03 (s, 1H), 8.02–7.99 (m, 1H), 7.97 (dd, *J* = 7.9, 1.5 Hz, 1H), 7.88–7.83 (m, 1H), 7.81–7.75 (m, 1H), 7.69–7.64 (m, 1H), 7.35–7.27 (m, 2H). ^13^C NMR (101 MHz, DMSO-*d*_6_) *δ* 162.4, 149.9, 148.6, 141.6, 140.3, 137.4, 135.7, 131.0, 128.7, 127.5, 127.0, 125.7, 124.5, 122.9, 122.3, 115.7, 114.1. HRMS (ESI), m/z: [M + Na]^+^ calcd. for C_17_H_11_N_3_O_2_ 312.0743; found 312.0746.

**6-**M**ethyl-3-(4-methylpyridin-2-yl)quinazoline-2,4(1*H*,3*H*)-dione 3r**. White powder; 77% yield (41 mg); mp 304–305 °C. ^1^H NMR (400 MHz, DMSO-*d*_6_) *δ* 11.53 (s, 1H), 8.44 (dd, *J* = 4.9, 0.8 Hz, 1H), 7.79–7.70 (m, 1H), 7.55 (dd, *J* = 8.4, 2.1 Hz, 1H), 7.37–7.28 (m, 2H), 7.16 (d, *J* = 8.2 Hz, 1H), 2.39 (s, 3H), 2.35 (s, 3H). ^13^C NMR (101 MHz, DMSO-*d*_6_) *δ* 162.4, 149.8, 149.6, 149.3, 148.9, 137.7, 136.5, 132.0, 126.8, 124.9, 124.8, 115.4, 113.9, 20.3, 20.2. HRMS (ESI), m/z: [M + H]^+^ calcd. for C_15_H_13_N_3_O_2_ 268.1081; found 268.1082.

**8-Methyl-3-(4-methylpyridin-2-yl)quinazoline-2,4(1*H*,3*H*)-dione 3s**. White powder; 74% yield (40 mg); mp 268–270 °C. ^1^H NMR (400 MHz, DMSO-*d*_6_) *δ* 10.85 (s, 1H), 8.51–8.41 (m, 1H), 7.82 (dd, *J* = 7.8, 0.8 Hz, 1H), 7.57 (d, *J* = 7.3 Hz, 1H), 7.40–7.29 (m, 2H), 7.16 (t, *J* = 7.6 Hz, 1H), 2.41 (s, 3H), 2.40 (s, 3H). ^13^C NMR (101 MHz, DMSO-*d*_6_) *δ* 162.2, 150.1, 149.7, 149.3, 148.9, 138.3, 136.5, 125.3, 125.0, 124.7, 124.3, 122.5, 114.3, 20.3, 17.2. HRMS (ESI), m/z: [M + H]^+^ calcd. for C_15_H_13_N_3_O_2_ 268.1081; found 268.1083.

**1-Methyl-3-(4-methylpyridin-2-yl)quinazoline-2,4(1*H*,3*H*)-dione 3t**. White powder; 32% yield (17 mg); mp 224–225 °C. ^1^H NMR (400 MHz, DMSO-*d*_6_) *δ* 8.45 (d, *J* = 5.1 Hz, 1H), 8.06 (dd, *J* = 7.8, 1.6 Hz, 1H), 7.89–7.82 (m, 1H), 7.54 (d, *J* = 8.4 Hz, 1H), 7.39–7.30 (m, 3H), 3.54 (s, 3H), 2.40 (s, 3H). ^13^C NMR (101 MHz, DMSO-*d*_6_) *δ* 161.2, 150.1, 149.7, 149.6, 148.9, 140.8, 135.8, 127.8, 125.0, 124.6, 122.9, 115.2, 114.9, 30.4, 20.3. HRMS (ESI), m/z: [M + H]^+^ calcd. for C_15_H_13_N_3_O_2_ 268.1081; found 268.1087.

**6,7-Dimethoxy-3-(4-methoxypyridin-2-yl)quinazoline-2,4(1*H*,3*H*)-dione 3u**. White powder; 74% yield (49 mg); mp 238–240 °C. ^1^H NMR (400 MHz, DMSO-*d*_6_) δ 11.39 (s, 1H), 8.39 (d, *J* = 5.7 Hz, 1H), 7.28 (s, 1H), 7.14–7.05 (m, 2H), 6.74 (s, 1H), 3.87 (s, 3H), 3.86 (s, 3H), 3.80 (s, 3H). ^13^C NMR (126 MHz, DMSO) δ 167.1, 161.5, 155.3, 151.0, 150.1, 149.9, 145.3, 135.7, 110.4 (2C), 107.5, 106.0, 97.7, 55.9, 55.8 (2C). HRMS (ESI), m/z: [M + H]^+^ calcd. for C_16_H_15_N_3_O_5_ 330.1084; found 330.1090.

**6-Fluoro-3-(4-methylpyridin-2-yl)quinazoline-2,4(1*H*,3*H*)-dione 3v**. White powder; 89% yield (48 mg); mp 293–294 °C. ^1^H NMR (400 MHz, DMSO-*d*_6_) *δ* 11.70 (s, 1H), 8.49–8.41 (m, 1H), 7.70–7.59 (m, 2H), 7.34 (d, *J* = 3.9 Hz, 2H), 7.32–7.26 (m, 1H), 2.39 (s, 3H). ^13^C NMR (101 MHz, DMSO-*d*_6_) *δ* 161.8 (d, *J* = 3.1 Hz), 157.9 (d, *J* = 240.4 Hz), 150.2, 150.1, 149.5, 149.4, 137.1 (d, *J* = 1.3 Hz), 125.5, 125.2, 124.0 (d, *J* = 24.4 Hz), 118.3 (d, *J* = 7.9 Hz), 115.7 (d, *J* = 8.0 Hz), 112.9 (d, *J* = 24.0 Hz), 20.8. ^19^F NMR (376 MHz, DMSO-*d*_6_) *δ* –119.63. HRMS (ESI), m/z: [M + H]^+^ calcd. for C_14_H_10_FN_3_O_2_ 272.0830; found 272.0829.

**6-Chloro-3-(4-methylpyridin-2-yl)quinazoline-2,4(1*H*,3*H*)-dione 3w**. White powder; 66% yield (38 mg); mp 301–302 °C. ^1^H NMR (400 MHz, DMSO-*d*_6_) *δ* 11.77 (s, 1H), 8.45 (dd, *J* = 4.8, 1.0 Hz, 1H), 7.87 (d, *J* = 2.5 Hz, 1H), 7.78 (dd, *J* = 8.7, 2.5 Hz, 1H), 7.37–7.31 (m, 2H), 7.27 (d, *J* = 8.7 Hz, 1H), 2.39 (s, 3H). ^13^C NMR (101 MHz, DMSO-*d*_6_) *δ* 161.1, 149.7, 149.6, 149.0, 148.9, 138.8, 135.4, 126.7, 126.3, 125.1, 124.7, 117.7, 115.6, 20.3. HRMS (ESI), m/z: [M + Na]^+^ calcd. for C_14_H_10_ClN_3_O_2_ 310.0354; found 310.0356.

**7-Chloro-3-(4-methylpyridin-2-yl)quinazoline-2,4(1*H*,3*H*)-dione 3x**. White powder; 47% yield (27 mg); mp 254–255 °C. ^1^H NMR (400 MHz, DMSO-*d*_6_) *δ* 11.74 (s, 1H), 8.54–8.38 (m, 1H), 7.94 (d, *J* = 8.4 Hz, 1H), 7.38–7.31 (m, 2H), 7.29 (dd, *J* = 8.4, 2.0 Hz, 1H), 7.26 (d, *J* = 1.9 Hz, 1H), 2.39 (s, 3H). ^13^C NMR (126 MHz, DMSO-*d*_6_) *δ* 161.4, 149.8, 149.7, 148.9, 141.0, 139.8, 129.6, 125.1, 124.7, 123.0, 114.8, 113.2, 20.3. HRMS (ESI), m/z: [M + Na]^+^ calcd. for C_14_H_10_ClN_3_O_2_ 310.0354; found 310.0351.

**6-Bromo-3-(4-methylpyridin-2-yl)quinazoline-2,4(1*H*,3*H*)-dione 3y**. Light beige powder; 62% yield (41 mg); mp 293–295 °C. ^1^H NMR (400 MHz, DMSO-*d*_6_) *δ* 11.77 (s, 1H), 8.45 (dd, *J* = 4.9, 0.9 Hz, 1H), 8.00 (d, *J* = 2.3 Hz, 1H), 7.89 (dd, *J* = 8.7, 2.3 Hz, 1H), 7.38–7.31 (m, 2H), 7.21 (d, *J* = 8.7 Hz, 1H), 2.39 (s, 3H). ^13^C NMR (101 MHz, DMSO-*d*_6_) *δ* 160.5, 149.2, 149.1, 148.5 (2C), 138.7, 137.5, 128.8, 124.6, 124.2, 117.4, 115.5, 113.7, 19.8. HRMS (ESI), m/z: [M + Na]^+^ calcd. for C_14_H_10_BrN_3_O_2_ 353.9849; found 353.9843.

**Methyl 3-(4-methylpyridin-2-yl)-2,4-dioxo-1,2,3,4-tetrahydroquinazoline- 7-carboxylate 3z**. White powder; 65% yield (41 mg); mp 266–267 °C (dec.). ^1^H NMR (400 MHz, DMSO-*d*_6_) *δ* 11.82 (s, 1H), 8.45 (d, *J* = 5.4 Hz, 1H), 8.07 (d, *J* = 8.2 Hz, 1H), 7.83 (d, *J* = 1.5 Hz, 1H), 7.75 (dd, *J* = 8.2, 1.6 Hz, 1H), 7.39–7.30 (m, 2H), 3.92 (s, 3H), 2.40 (s, 3H). ^13^C NMR (101 MHz, DMSO-*d*_6_) *δ* 165.1, 161.5, 149.8, 149.8, 149.0 (2C), 140.0, 135.3, 128.2, 125.1, 124.7, 122.5, 117.4, 116.3, 52.8, 20.3. HRMS (ESI), m/z: [M + H]^+^ calcd. for C_16_H_13_BrN_3_O_4_ 312.0979; found 312.0972.

**Ethyl 2-(3-(5-cyanopyridin-2-yl)ureido)benzoate 4b**. Compound **4b** was obtained according to the *general procedure A* from urea **1r** (0.15 mmol) and ethyl anthranilate (0.75 mmol). Beige powder; 40% yield (19 mg); mp 193–195 °C. ^1^H NMR (400 MHz, DMSO-*d*_6_) *δ* 11.11 (s, 1H), 10.69 (s, 1H), 8.73 (d, *J* = 2.3 Hz, 1H), 8.29–8.22 (m, 1H), 8.17 (dd, *J* = 8.8, 2.3 Hz, 1H), 7.93 (dd, *J* = 7.9, 1.7 Hz, 1H), 7.68 (d, *J* = 8.8 Hz, 1H), 7.63–7.55 (m, 1H), 7.20–7.13 (m, 1H), 4.35 (q, *J* = 7.1 Hz, 2H), 1.33 (t, *J* = 7.1 Hz, 3H). ^13^C NMR (101 MHz, DMSO-*d*_6_) *δ* 166.4, 155.2, 151.8, 151.6, 141.5, 139.6, 133.5, 130.5, 122.6, 121.9, 118.1, 117.4, 111.8, 101.7, 61.0, 14.0. HRMS (ESI), m/z: [M + H]^+^ calcd. for C_16_H_14_N_4_O_3_ 311.1139; found 311.1149.

### 3.4. Synthesis of Thienopyrimidine-2,4-Diones ***8***

*General procedure C*. Urea **1** (0.2 mmol), amino ester **6** (0.3 mmol), and DMF (0.1 mL) were placed in a vial; the resulting mixture was stirred at 120 °C for 20 h. Then, the reaction mixture was cooled to RT and sodium *tert*-butoxide (0.2 mmol) and DMF (0.6 mL) were added. The reaction mixture was stirred for another 2 h at 120 °C. After completion of the reaction, the reaction mixture was cooled to RT, and DMF was removed by a rotary evaporator. The residue was purified by column chromatography (gradient from *n*–hexane/ethyl acetate to hexane/ethyl acetate/methanol) to give compounds **8a**–**f**.

**3-(4-Methylpyridin-2-yl)-5,6,7,8-tetrahydrobenzo [4,5]thieno [2,3-*d*]pyrimidine-2,4(1*H*,3*H*)-dione 8a**. White powder; 54% yield (34 mg); mp 281–282 °C. ^1^H NMR (400 MHz, DMSO-*d*_6_) *δ* 12.24 (br s, 1H), 8.41 (d, *J* = 5.0 Hz, 1H), 7.29 (d, *J* = 5.0 Hz, 1H), 7.23 (s, 1H), 2.67 (dt, *J* = 29.1, 6.4 Hz, 4H), 2.37 (s, 3H), 1.82–1.65 (m, 4H). ^13^C NMR (101 MHz, DMSO-*d*_6_) *δ* 158.8, 150.5, 150.2, 149.5, 149.5, 148.9, 131.0, 125.7, 124.9, 124.8, 112.4, 24.9, 23.9, 22.7, 21.6, 20.3. HRMS (ESI), m/z: [M + H]^+^ calcd. for C_16_H_15_N_3_O_2_S 314.0958; found 314.0960.

**3-(Pyridin-2-yl)-5,6,7,8-tetrahydrobenzo [4,5]thieno [2,3-*d*]pyrimidine-2,4(1*H*,3*H*)-dione 8b**. White powder; 50% yield (30 mg); mp 276–278 °C. ^1^H NMR (400 MHz, DMSO-*d*_6_) *δ* 12.26 (s, 1H), 8.58 (dd, *J* = 5.0, 1.9 Hz, 1H), 7.97 (td, *J* = 7.7, 2.0 Hz, 1H), 7.51–7.41 (m, 2H), 2.76–2.61 (m, 4H), 1.83–1.67 (m, 4H). ^13^C NMR (101 MHz, DMSO-*d*_6_) *δ* 158.7, 150.1 (2C), 149.4, 149.3, 138.6, 131.1, 125.9, 124.5, 123.9, 112.5, 24.9, 23.9, 22.7, 21.6. HRMS (ESI), m/z: [M + H]^+^ calcd. for C_15_H_13_N_3_O_2_S 300.0801; found 300.0804.

**3-(4-Methoxypyridin-2-yl)-5,6,7,8-tetrahydrobenzo [4,5]thieno [2,3-*d*]pyrimidine-2,4(1*H*,3*H*)-dione 8c**. White powder; 45% yield (29 mg); mp 257–260 °C.^1^H NMR (400 MHz, DMSO-*d*_6_) *δ* 12.23 (s, 1H), 8.40–8.34 (m, 1H), 7.10–7.01 (m, 2H), 3.85 (s, 3H), 2.68 (dt, *J* = 29.4, 6.3 Hz, 4H), 1.83–1.66 (m, 4H). ^13^C NMR (101 MHz, DMSO-*d*_6_) *δ* 167.0, 158.6, 150.9, 150.1, 150.1, 150.0, 131.0, 125.8, 112.5, 110.4, 110.3, 55.7, 24.9, 23.8, 22.7, 21.6. HRMS (ESI), m/z: [M + H]^+^ calcd. for C_16_H_15_N_3_O_3_S 330.0907; found 330.0910.

**3-(6-Phenylpyridin-2-yl)-5,6,7,8-tetrahydrobenzo [4,5]thieno [2,3-*d*]pyrimidine-2,4(1*H*,3*H*)-dione 8d**. Light yellow powder; 48% yield (36 mg); mp 293–294 °C. ^1^H NMR (400 MHz, DMSO-*d*_6_) *δ* 12.30 (s, 1H), 8.13–7.93 (m, 4H), 7.56–7.38 (m, 4H), 2.70 (dt, *J* = 29.8, 6.3 Hz, 4H), 1.88–1.64 (m, 4H). ^13^C NMR (101 MHz, DMSO-*d*_6_) *δ* 158.7, 156.3, 150.1, 150.1, 149.3, 139.7, 137.7, 131.0, 129.4, 128.8, 126.6, 125.9, 123.1, 120.2, 112.6, 24.9, 23.9, 22.7, 21.6. HRMS (ESI), m/z: [M + Na]^+^ calcd. for C_21_H_17_N_3_O_3_S 398.0934; found 398.0939.

**3-(Quinolin-2-yl)-5,6,7,8-tetrahydrobenzo [4,5]thieno [2,3-*d*]pyrimidine-2,4(1*H*,3*H*)-dione 8e**. Light yellow powder; 56% yield (39 mg); mp 308–310 °C (dec.). ^1^H NMR (400 MHz, DMSO-*d*_6_) *δ* 12.35 (s, 1H), 8.53 (d, *J* = 8.5 Hz, 1H), 8.09 (dd, *J* = 8.4, 0.8 Hz, 1H), 8.01 (dd, *J* = 8.4, 1.0 Hz, 1H), 7.87–7.80 (m, 1H), 7.74–7.68 (m, 1H), 7.58 (d, *J* = 8.5 Hz, 1H), 2.70 (dt, *J* = 26.9, 6.3 Hz, 4H), 1.84–1.66 (m, 4H). ^13^C NMR (101 MHz, DMSO-*d*_6_) *δ* 158.9, 150.3, 150.2, 149.2, 147.0, 138.7, 131.1, 130.0, 128.6, 127.9, 127.6, 127.4, 125.9, 122.3, 112.6, 24.9, 23.9, 22.7, 21.6. HRMS (ESI), m/z: [M + H]^+^ calcd. for C_19_H_15_N_3_O_3_S 350.0958; found 350.0957.

**3-(4-Methylpyridin-2-yl)-1,5,6,7-tetrahydro-2*H*-cyclopenta [4,5]thieno [2,3-*d*]pyrimidine-2,4(3*H*)-dione 8f**. Brown powder; 35% yield (21 mg); mp 103–104 °C. ^1^H NMR (400 MHz, DMSO-*d*_6_) *δ* 12.31 (s, 1H), 8.42 (d, *J* = 5.0 Hz, 1H), 7.31 (d, *J* = 5.2 Hz, 1H), 7.27 (s, 1H), 2.86–2.75 (m, 4H), 2.41–2.32 (m, 5H). ^13^C NMR (101 MHz, DMSO-*d*_6_) *δ* 158.3, 154.6, 150.2, 149.6, 149.3, 148.9, 140.1, 130.7, 124.9, 109.8, 28.4, 28.3, 27.5, 20.3. HRMS (ESI), m/z: [M + Na]^+^ calcd. for C_15_H_13_N_3_O_2_S 322.0621; found 322.0618.

***Preparation of ethyl 2-(3-(4-methylpyridin-2-yl)ureido)-4,5,6,7-tetrahydrobenzo[b]thiophene-3-carboxylate 7a*.** Urea **1** (0.2 mmol), ethyl 2-amino-4,5,6,7-tetrahydrobenzo[*b*]thiophene-3-carboxylate **6a** (0.3 mmol) and DMF (0.1 mL) were placed in a vial, the resulting mixture was stirred at 120 °C for 20 h. After completion of the reaction, the reaction mixture was cooled to RT, DMF was removed by a rotary evaporator. The residue was washed with diethyl ether to give compound **7a** in 56% yield (40 mg) as a white powder; mp 237–238 °C. ^1^H NMR (400 MHz, DMSO-*d*_6_) *δ* 13.21 (br s, 1H), 10.27 (s, 1H), 8.17 (d, *J* = 5.2 Hz, 1H), 7.02 (s, 1H), 6.90 (dd, *J* = 5.2, 1.6 Hz, 1H), 4.32 (q, *J* = 7.1 Hz, 2H), 2.76–2.66 (m, 2H), 2.64–2.54 (m, 2H), 2.29 (s, 3H), 1.77–1.67 (m, 4H), 1.30 (t, *J* = 7.1 Hz, 3H). ^13^C NMR (101 MHz, DMSO-*d*_6_) *δ* 164.2, 152.3, 151.5, 149.6, 147.4, 146.0, 130.4, 125.2, 119.1, 111.9, 110.6, 59.7, 26.0, 23.7, 22.5, 22.4, 20.8, 14.3. HRMS (ESI), m/z: [M + H]^+^ calcd. for C_18_H_21_N_3_O_3_S 360.1376; found 360.1374.

### 3.5. Gram-Scale Synthesis of ***3a*** and ***8a***

**3a**. Urea **1a** (5.6 mmol, 1 g) and ethyl anthranilate **2a** (27.9 mmol, 4.61 g) were placed in a 100 mL round-bottom flask and the resulting mixture was stirred at 120 °C for 20 h. The reaction mixture was cooled to RT, treated with diethyl ether (20 mL), and the precipitate was separated. Then, the precipitate was washed with diethyl ether and dried at 50 °C in air to give quinazoline-2,4-dione **3a** in 71% (1.01 g) yield.

**8a**. Urea **1a** (5.6 mmol, 1 g), ester **6a** (8.4 mmol, 1.89 g), and DMF (4 mL) were placed in a 100 mL round-bottom flask, the resulting mixture was stirred at 120 °C for 20 h. Then, the reaction mixture was cooled to RT and sodium *tert*-butoxide (5.6 mmol, 0.54 g) and DMF (17 mL) were added. The reaction mixture was stirred for another 2 h at 120 °C. After completion of the reaction, the reaction mixture was cooled to RT, and DMF was removed by a rotary evaporator. The residue was dissolved in isopropyl alcohol (150 mL) and the resultant solution was diluted with water (350 mL). The precipitate formed was filtered off and dried at 50 °C in air to give thienopyrimidine-2,4-dione **8a** in 61% (1.06 g) yield.

## 4. Conclusions

Thus, we developed a new route to 3-pyridyl-substituted quinazolin-2,4(1*H,*3*H*)-diones and thieno [2,3-*d*]pyrimidine-2,4(1*H*,3*H*)-diones via the annulation of anthranilic esters with *N*-pyridyl ureas, which act as masked isocyanates. The process consists of the formation of *N*-aryl-*N*′-pyridyl ureas followed by their cyclocondensation into the corresponding diones. The reaction does not require the use of metal catalysts and proceeds with moderate to good yields. The synthetic route we propose will successfully complement the method developed by Ravi et al. [58] for the preparation of quinazolin-2,4(1*H*,3*H*)-diones based on aminopyridines in cases where the corresponding aminopyridines or quinolines are not commercially available.

Although the nature of the substituent in the pyridine ring has little effect on the product yield, strong electron-withdrawing functionalities such as cyano-group decrease the yield of the desired products or even prevent the cyclocondensation step. The proposed method is characterized by uncomplicated workup and easy gram-scalability.

## Figures and Tables

**Table 1:** Optimization of the reaction conditions.

Entry	Equiv. of 2a	Solvent	Temperature, °C	Time, h	Yield, %
1	1.2	DMF	120	24	52
2	1.2	DMF	140	24	50
3	1.5	DMF	120	20	58
4	2.0	DMF	120	20	62
5	5.0	DMF	120	20	63
6	5.0	neat	120	20	70
7	10.0	neat	120	20	59
8 *	1.5	DMF	120	20	46

* Anthranilic acid was used instead ester **2a**.

## Data Availability

Not applicable.

## Supplementary Materials

The supporting information can be downloaded at: https://www.mdpi.com/article/10.3390/ijms24087633/s1.
