# Supplementary material for: Synthesis of 3-(Pyridin-2-yl)quinazolin-2,4(1H,3H)-diones via Annulation of Anthranilic Esters with N-pyridyl Ureas
Source: Int J Mol Sci. 2023 Apr 21;24(8):7633. doi: 10.3390/ijms24087633 (PMC10142796; doi:10.3390/ijms24087633)
Supplement: Supplementary file 1 [file ijms-24-07633-s001.zip › ijms-2344096-supplementary.pdf]

**Supporting information**
**for**

# Synthesis of 3-(pyridin-2-yl)quinazolin-2,4(1*H*,3*H*)-diones via annulation of anthranilic esters with *N*-pyridyl ureas

Svetlana O. Baykova, Kirill K. Geyl, Sergey V. Baykov, Vadim P. Boyarskiy *

*Institute of Chemistry, Saint Petersburg State University, Universitetskaya Nab., 7/9, 199034 Saint Petersburg, Russia*

*Corresponding authors: v.boyarskii@spbu.ru.

**Table of Content**

Copies of $^{1}H$, $^{13}C$, and $^{19}F$ NMR spectra ........................................................ 3
Table S1 Crystal data and structure refinement for **3a**. ........................................ 42

## Copies of $^{1}H$, $^{13}C$, and $^{19}F$ NMR spectra

$^{1}H$ and $^{13}C$ spectra of methyl 6-(3,3-dimethylureido)picolinate **1h**

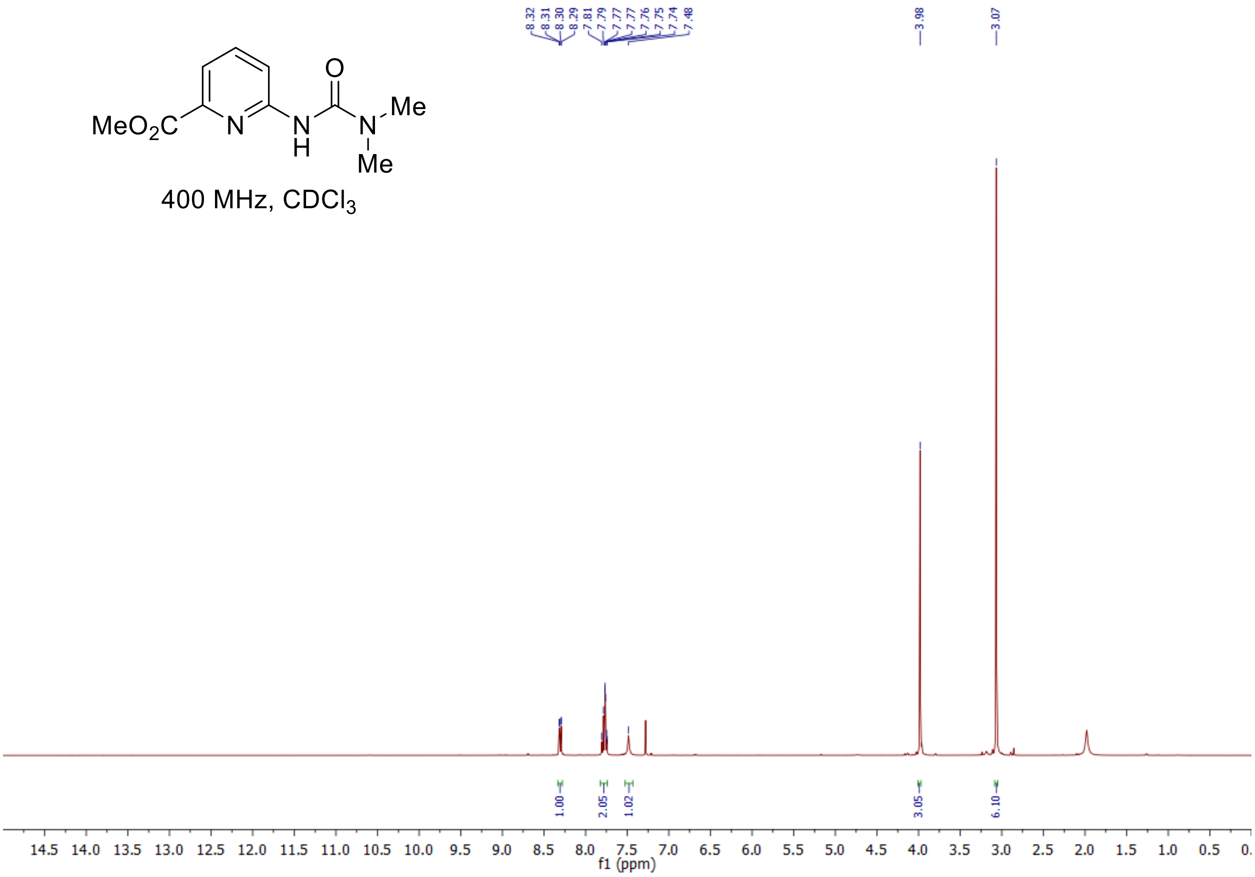


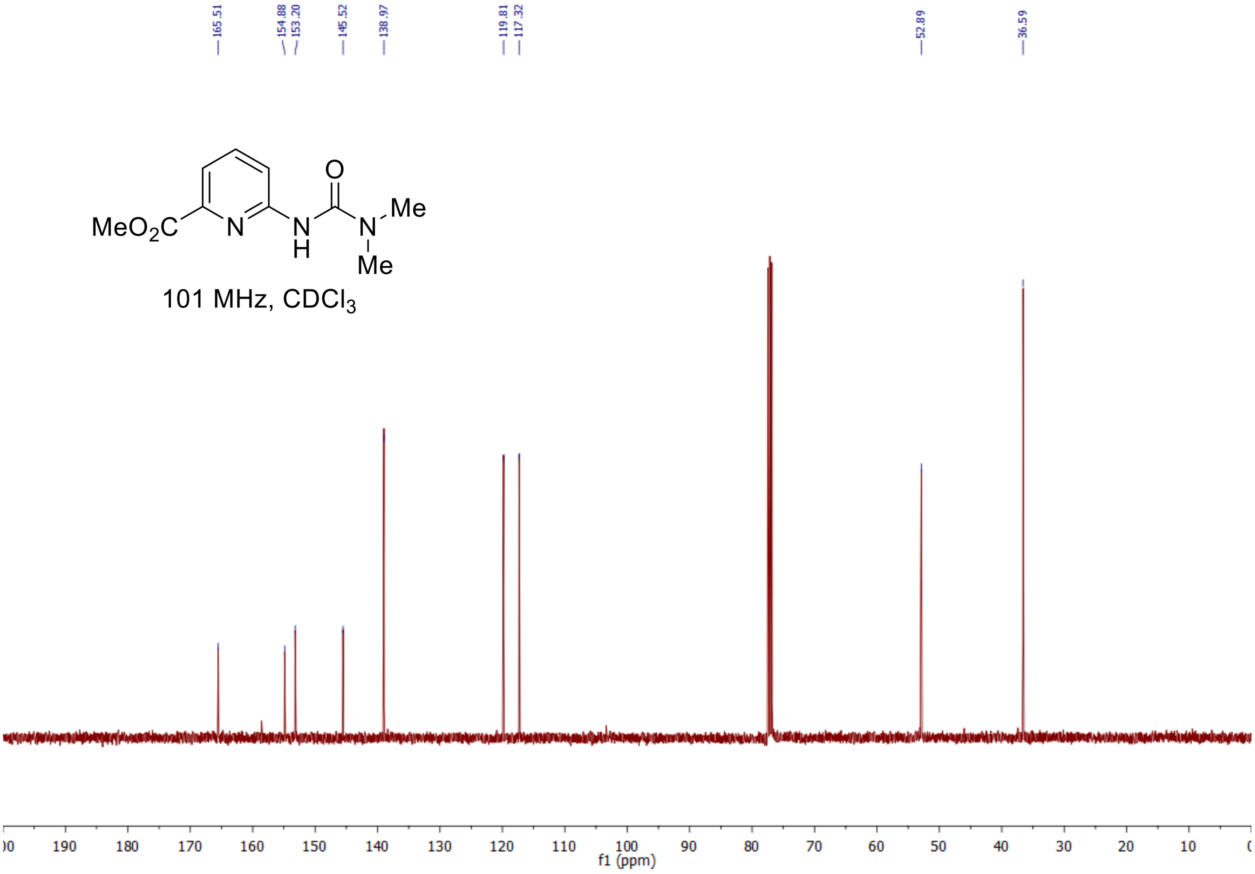

$^{1}H$ and $^{13}C$ spectra of 1,1-dimethyl-3-(6-methylquinolin-2-yl)urea **1n**

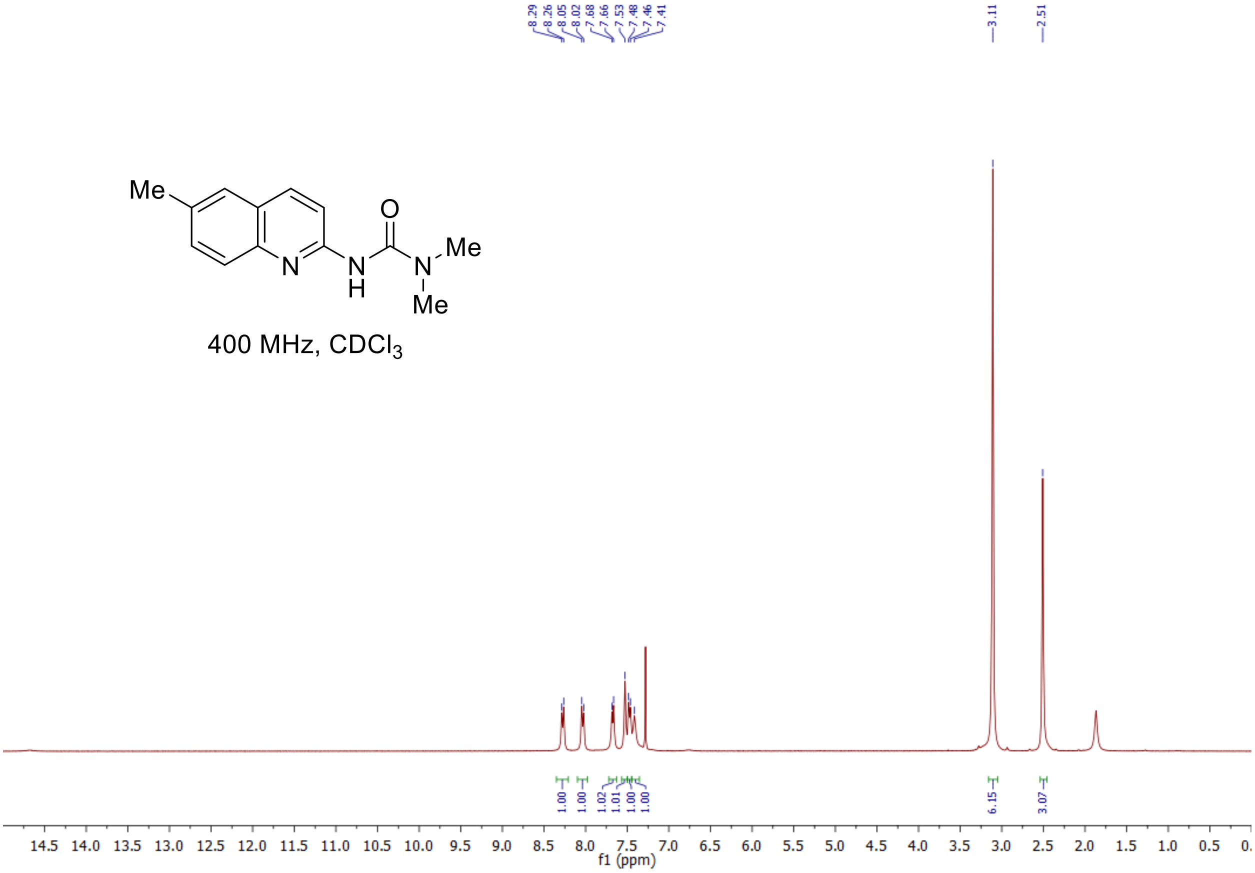


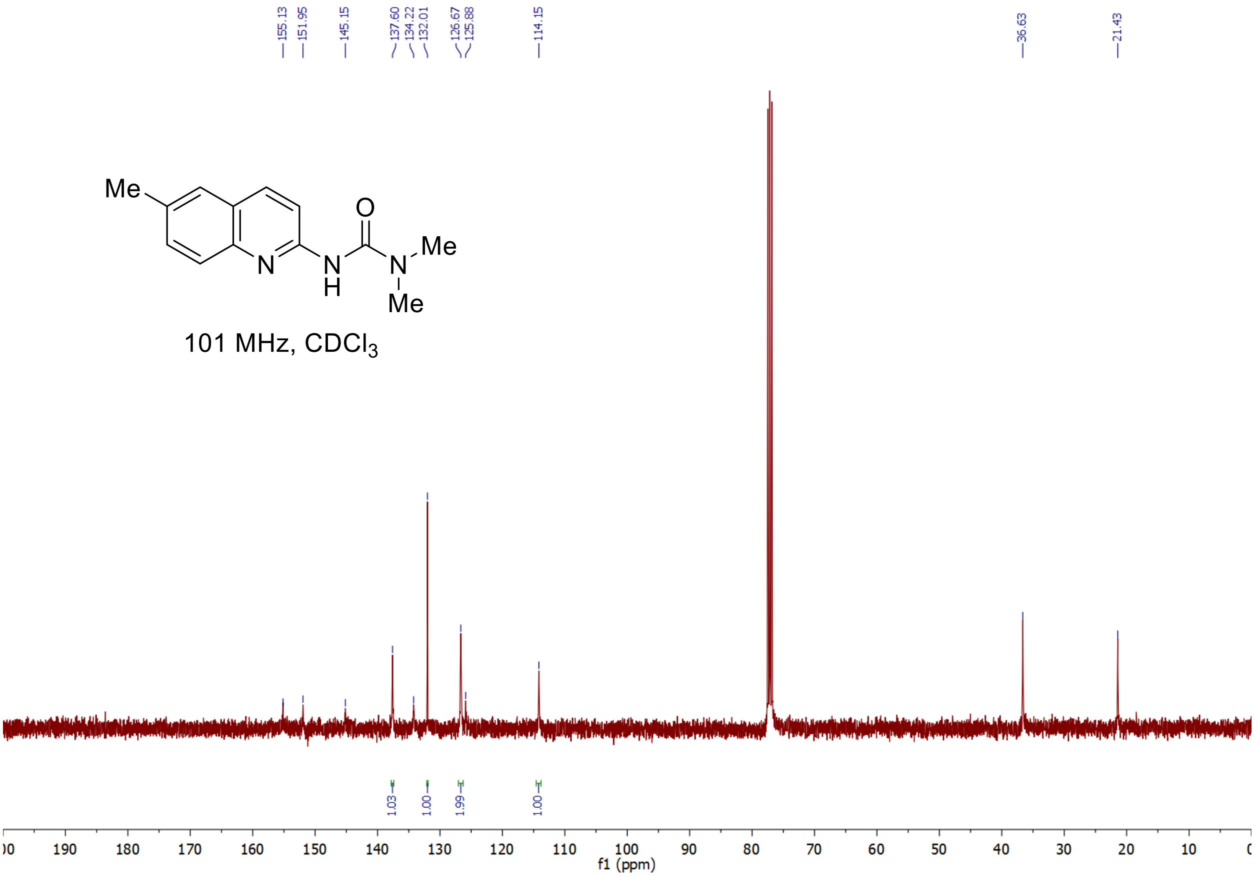

$^{1}H$ and $^{13}C$ spectra of 1,1-dimethyl-3-(7-methylquinolin-2-yl)urea **1o**

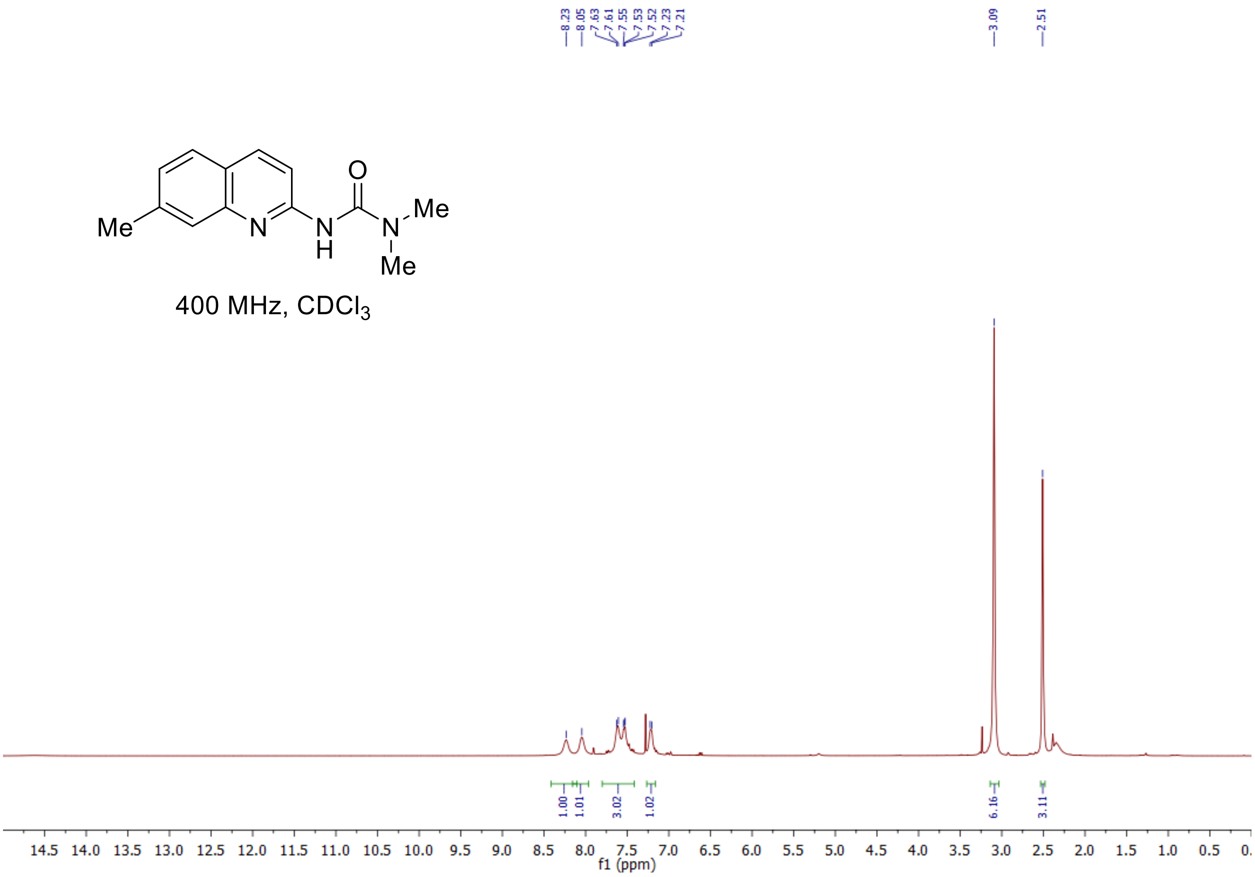


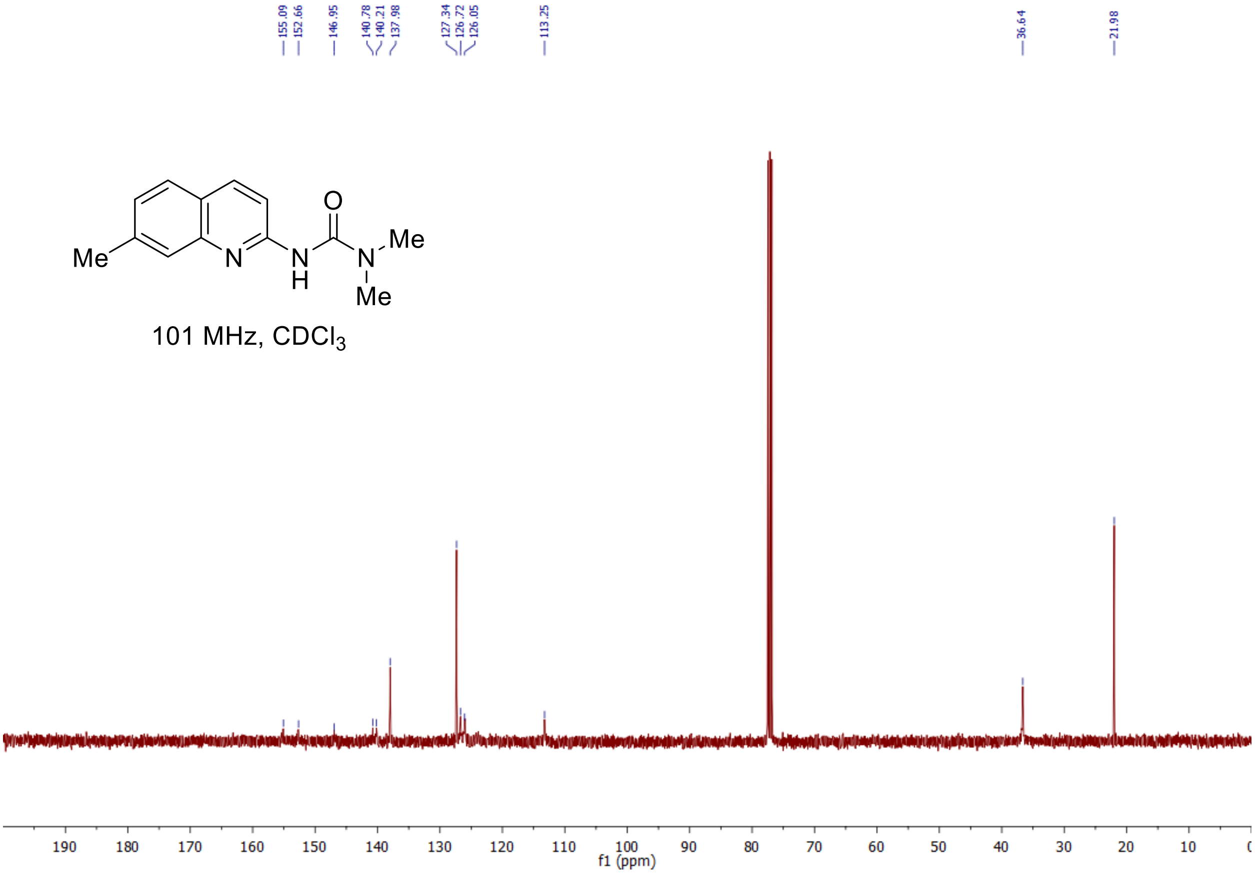

$^{1}$H and $^{13}$C spectra of 3-(6-methoxyquinolin-2-yl)-1,1-dimethylurea **1p**

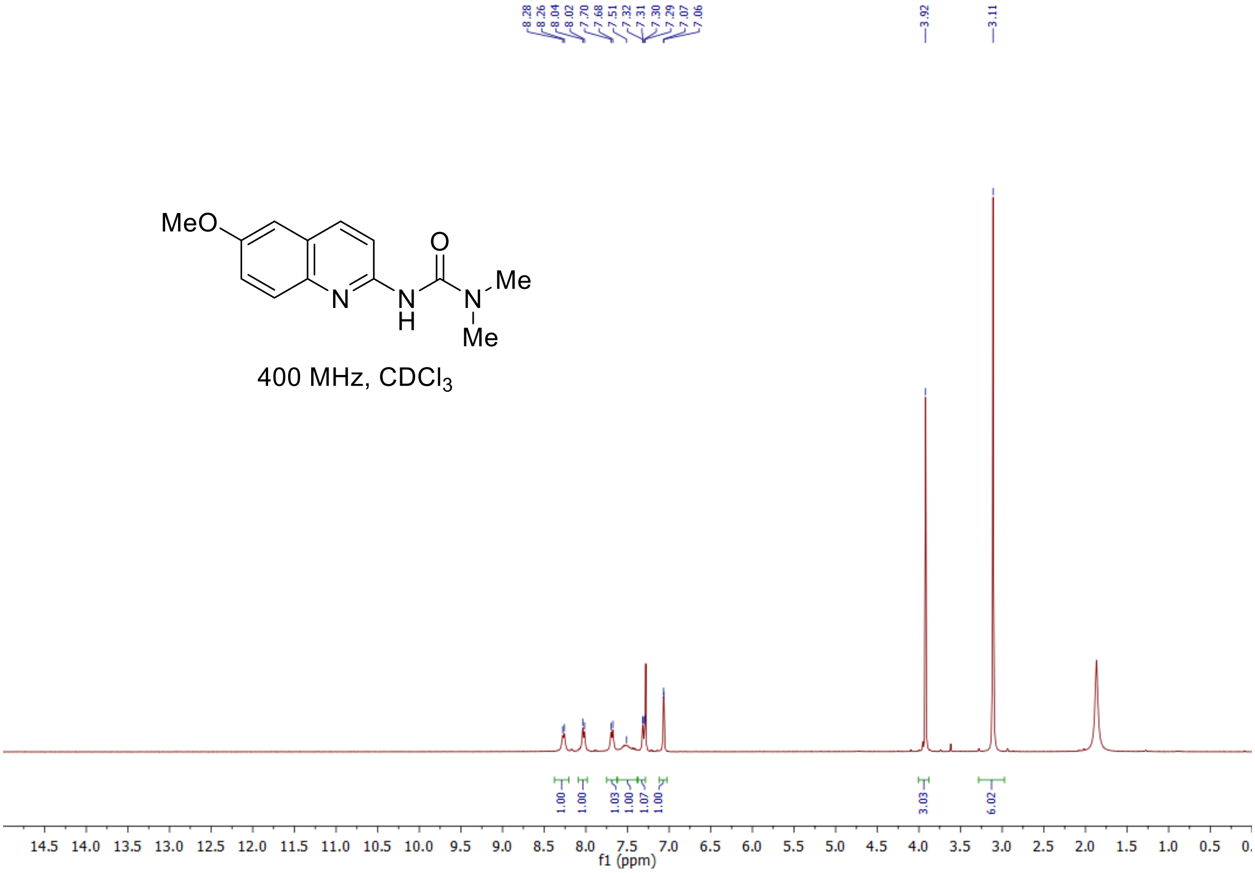


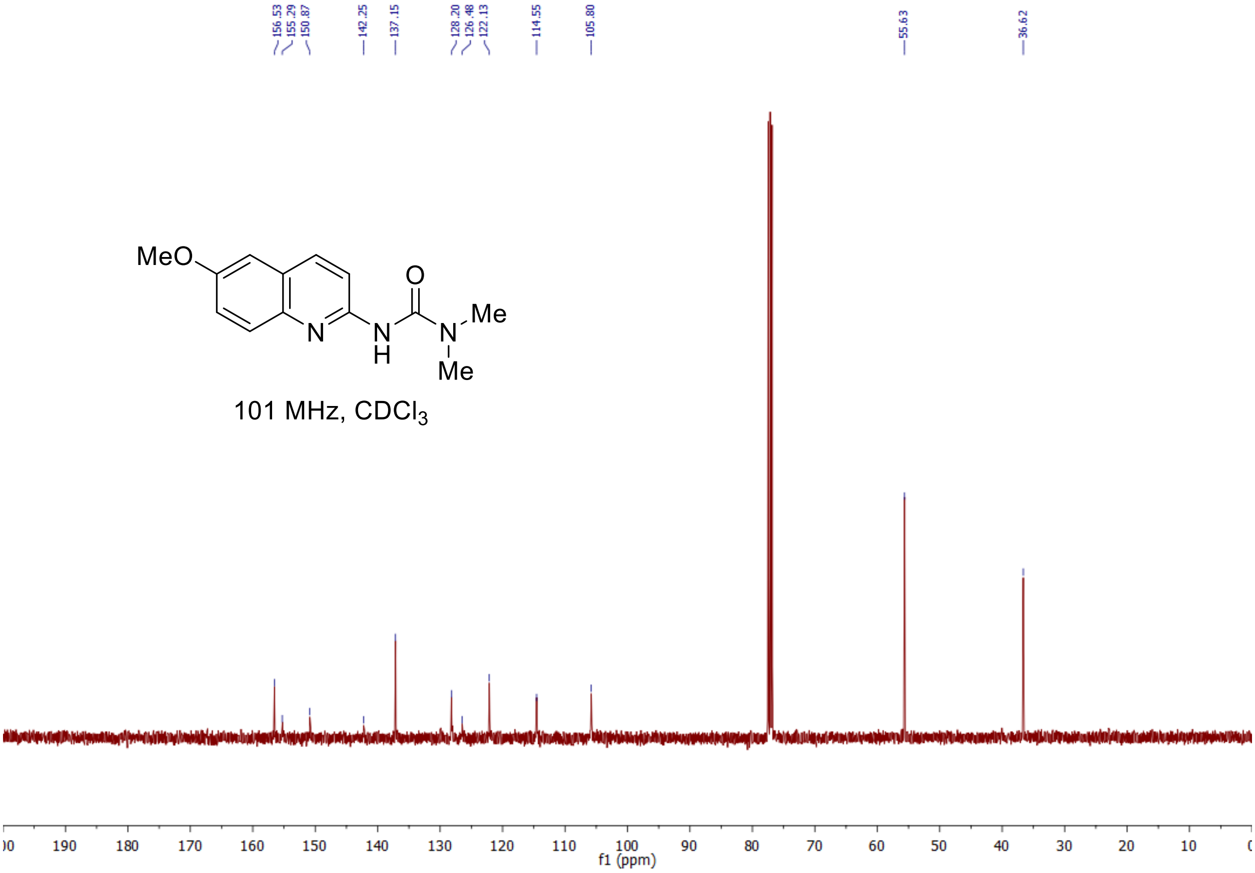

$^{1}$H and $^{13}$C spectra of 3-(4-methylpyridin-2-yl)quinazoline-2,4(1*H*,3*H*)-dione **3a**

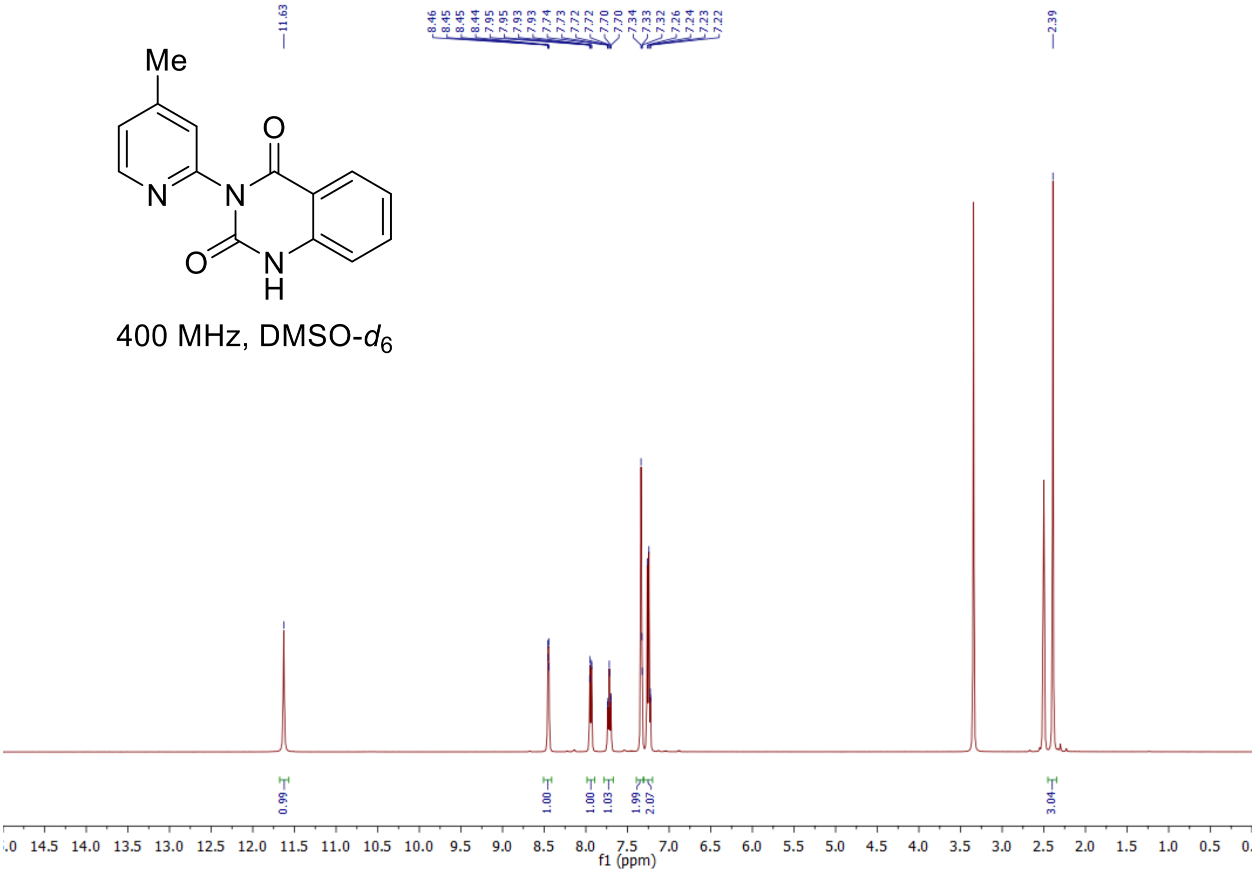


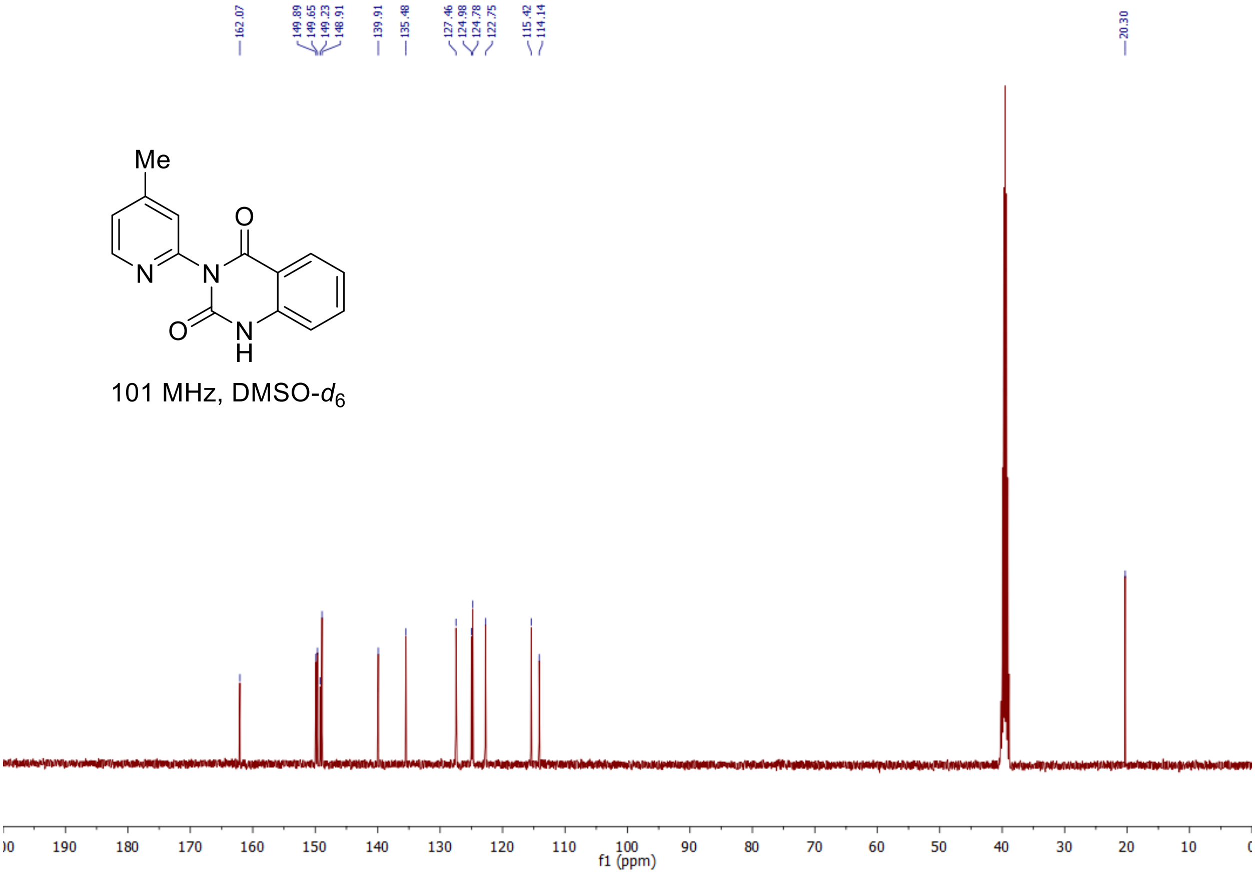

$^{1}$H and $^{13}$C spectra of 3-(pyridin-2-yl)quinazoline-2,4(1*H*,3*H*)-dione **3b**

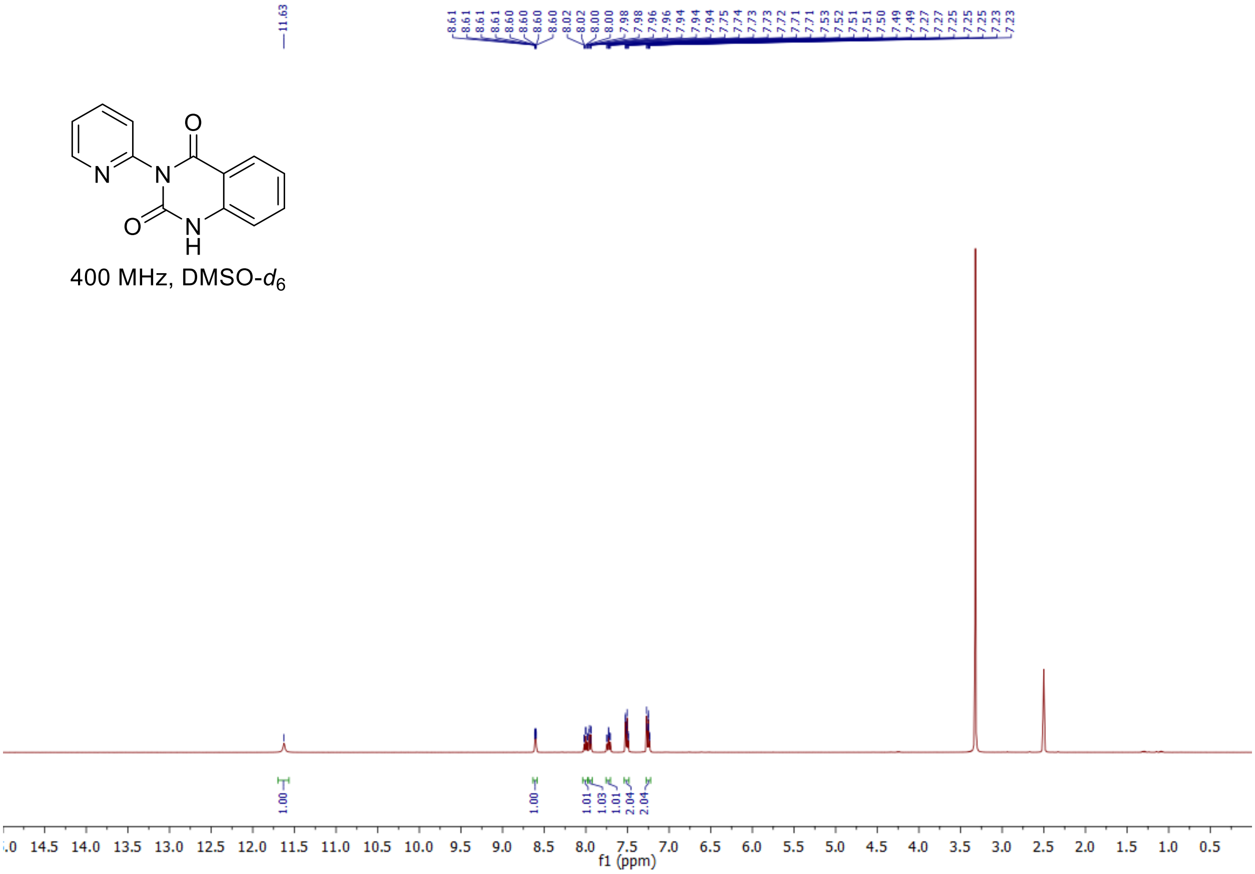


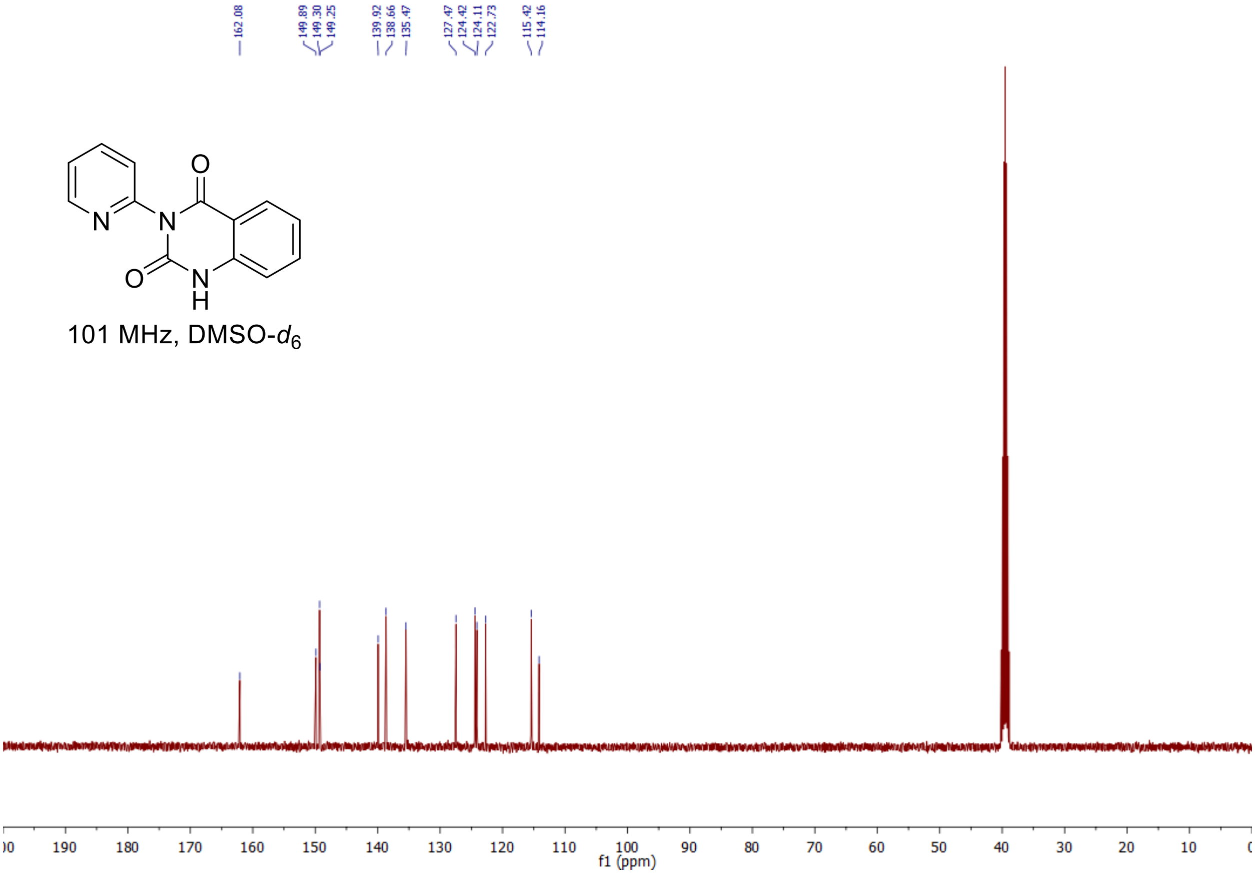

$^{1}H$ and $^{13}C$ spectra of 3-(5-methylpyridin-2-yl)quinazoline-2,4(1*H*,3*H*)-dione **3c**

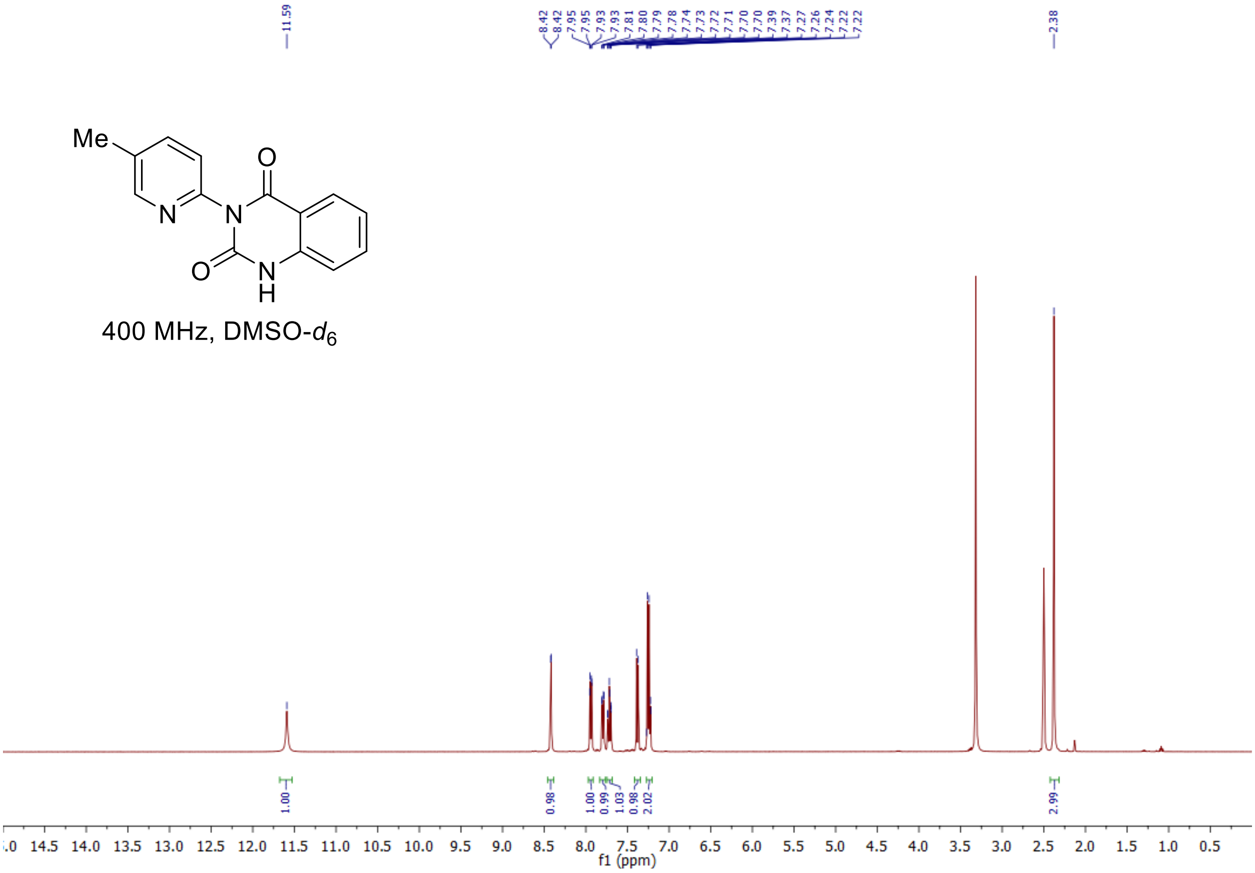


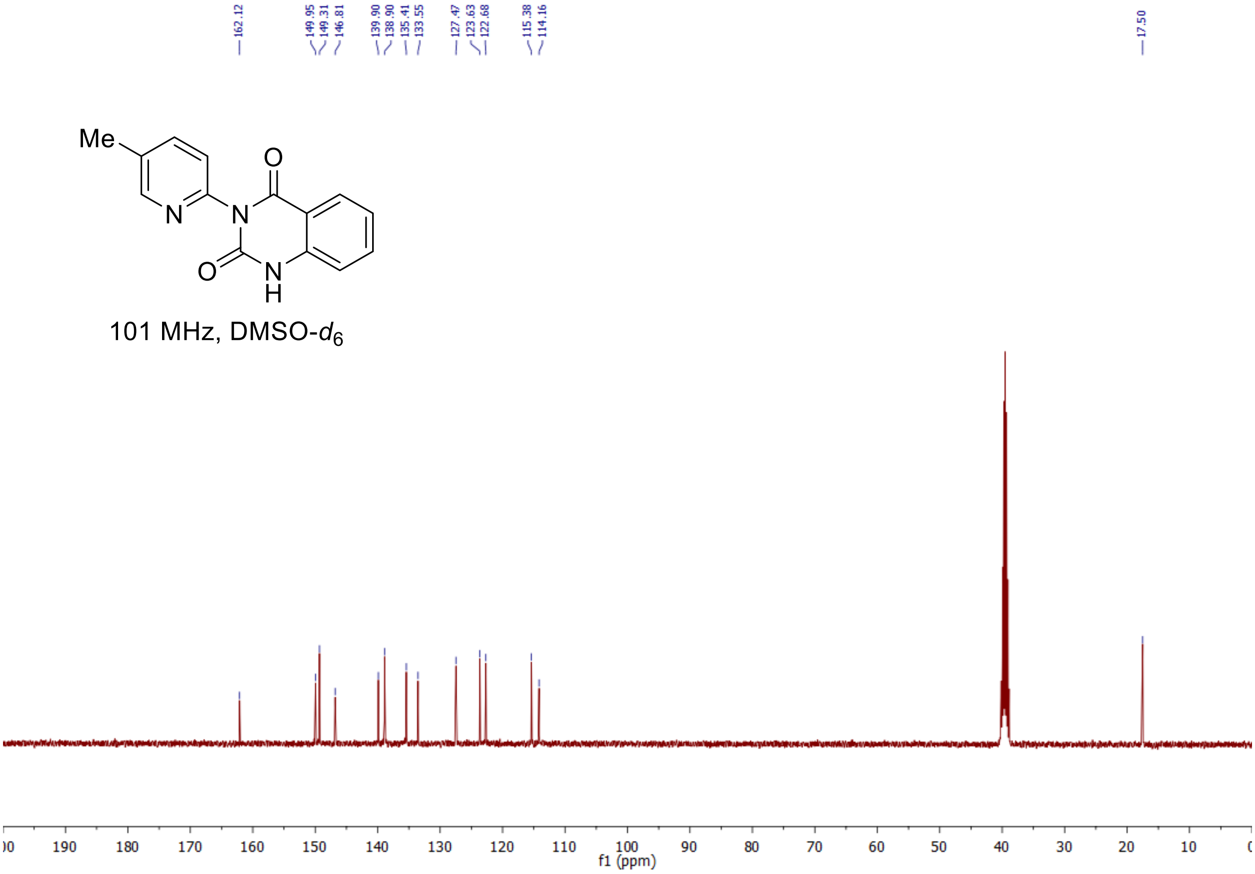

$^{1}$H and $^{13}$C spectra of 3-(6-methylpyridin-2-yl)quinazoline-2,4(1*H*,3*H*)-dione **3d**

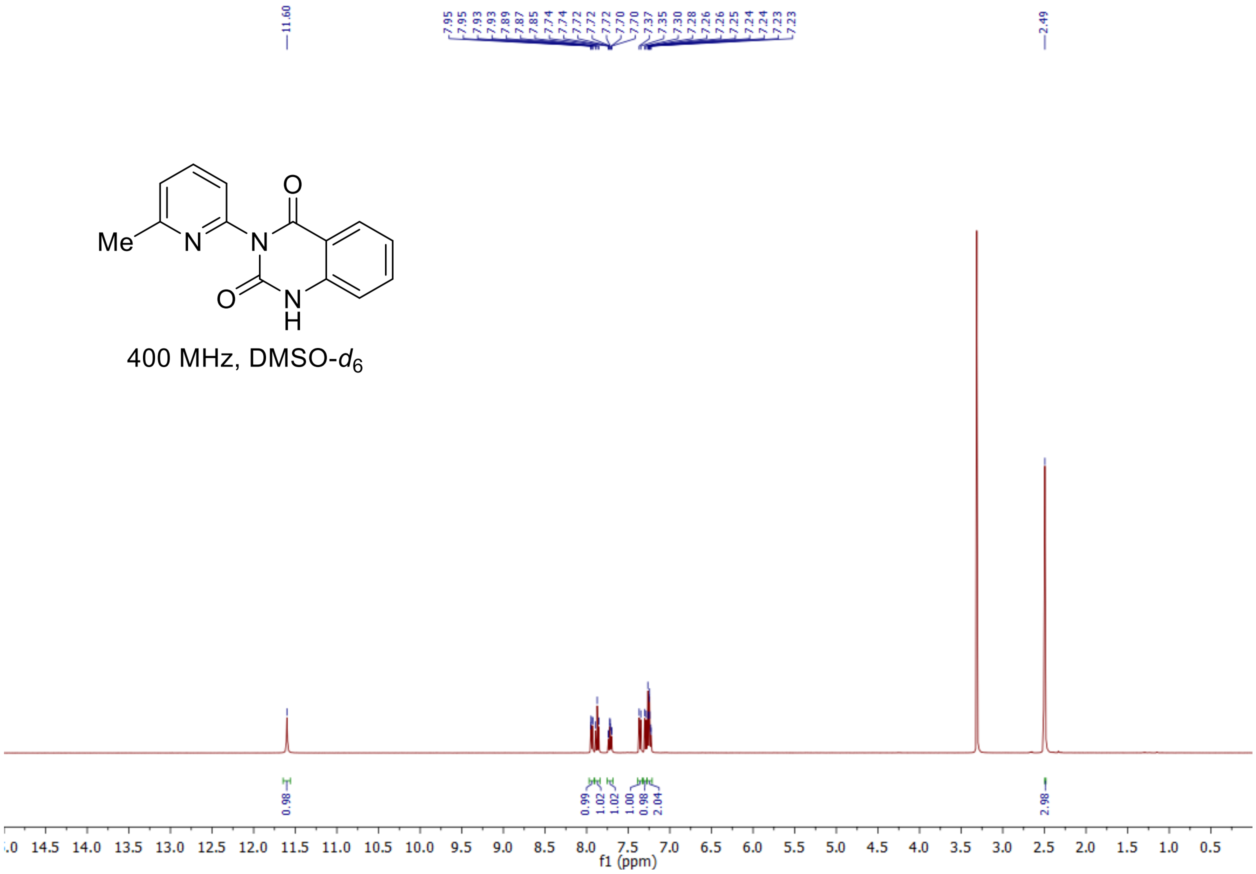


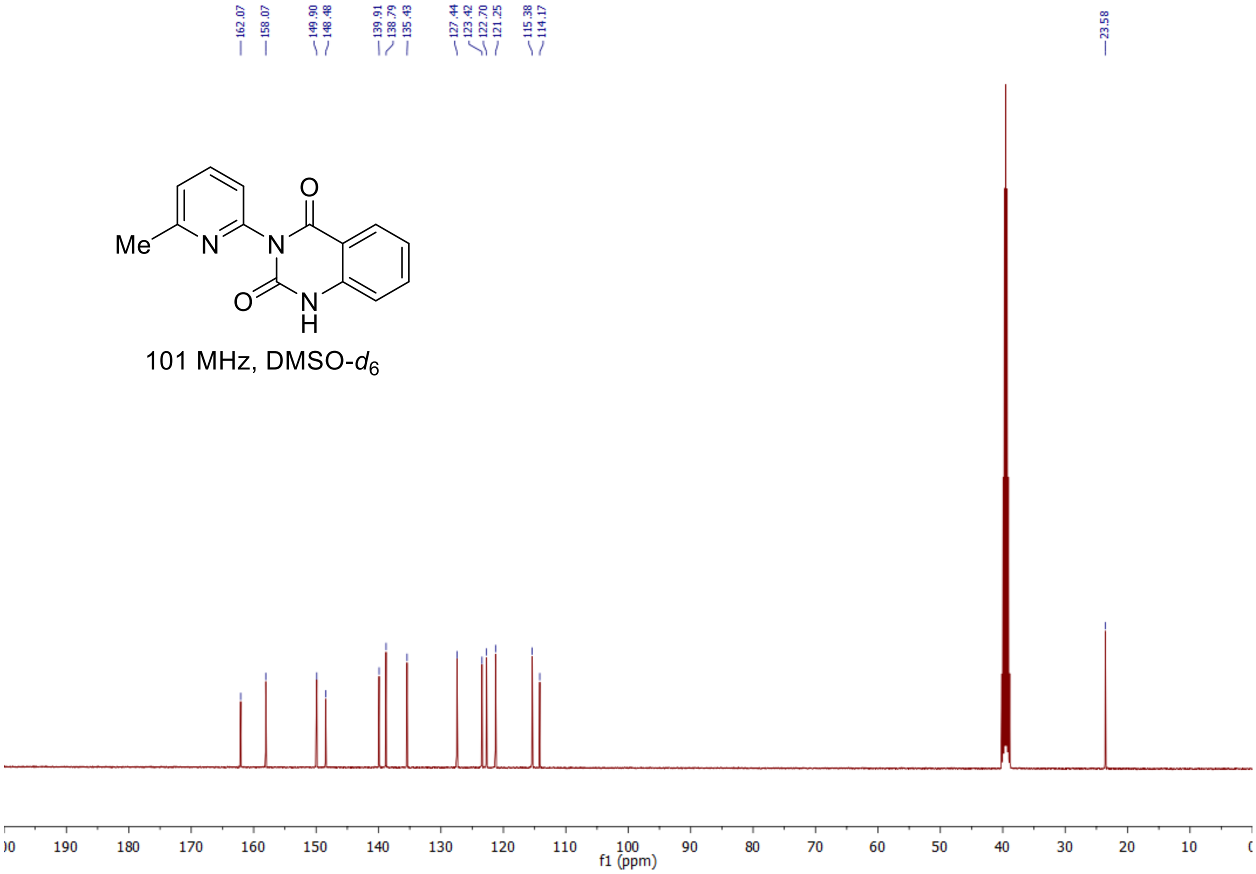

<sup></sup>

$^{1}$H and $^{13}$C spectra of 3-(3,5-dimethylpyridin-2-yl)quinazoline-2,4(1*H*,3*H*)-dione **3e**

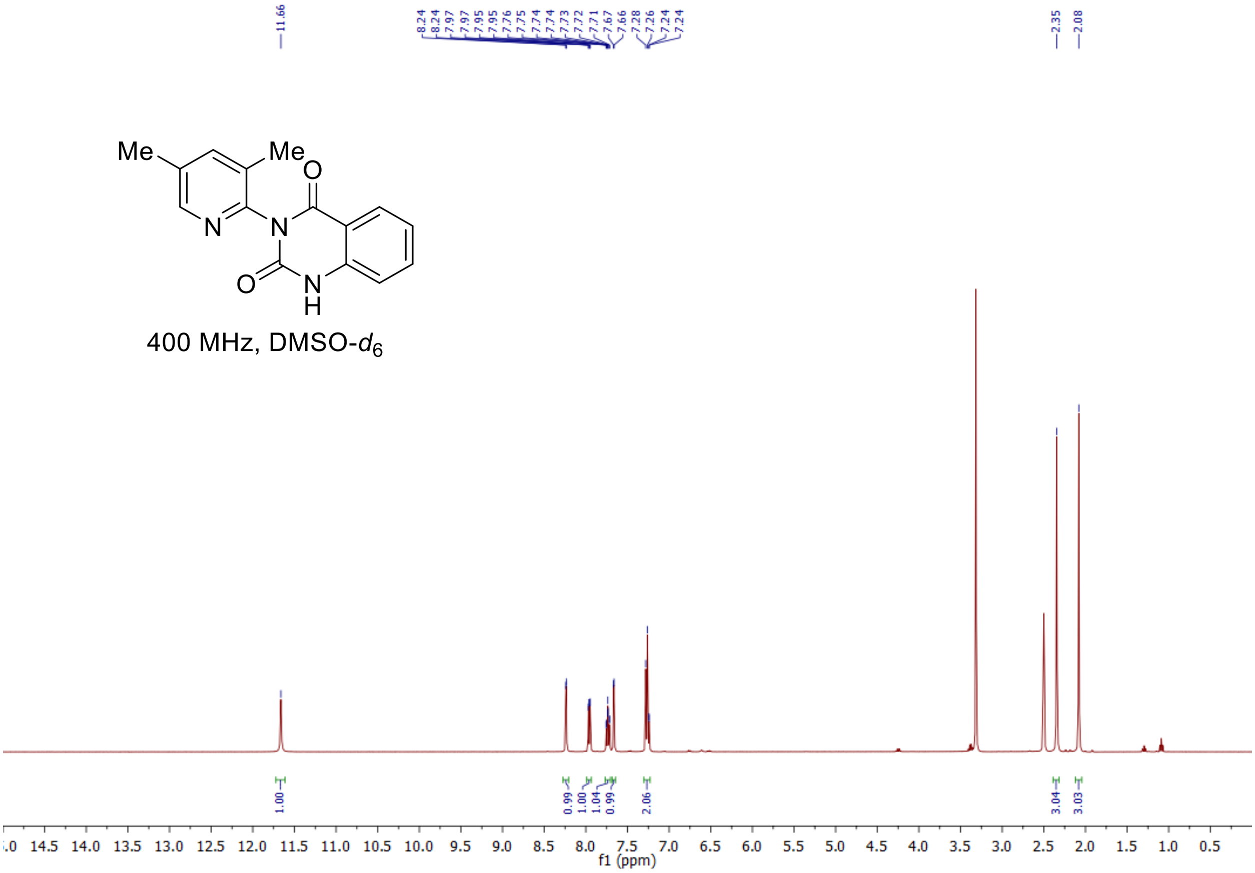


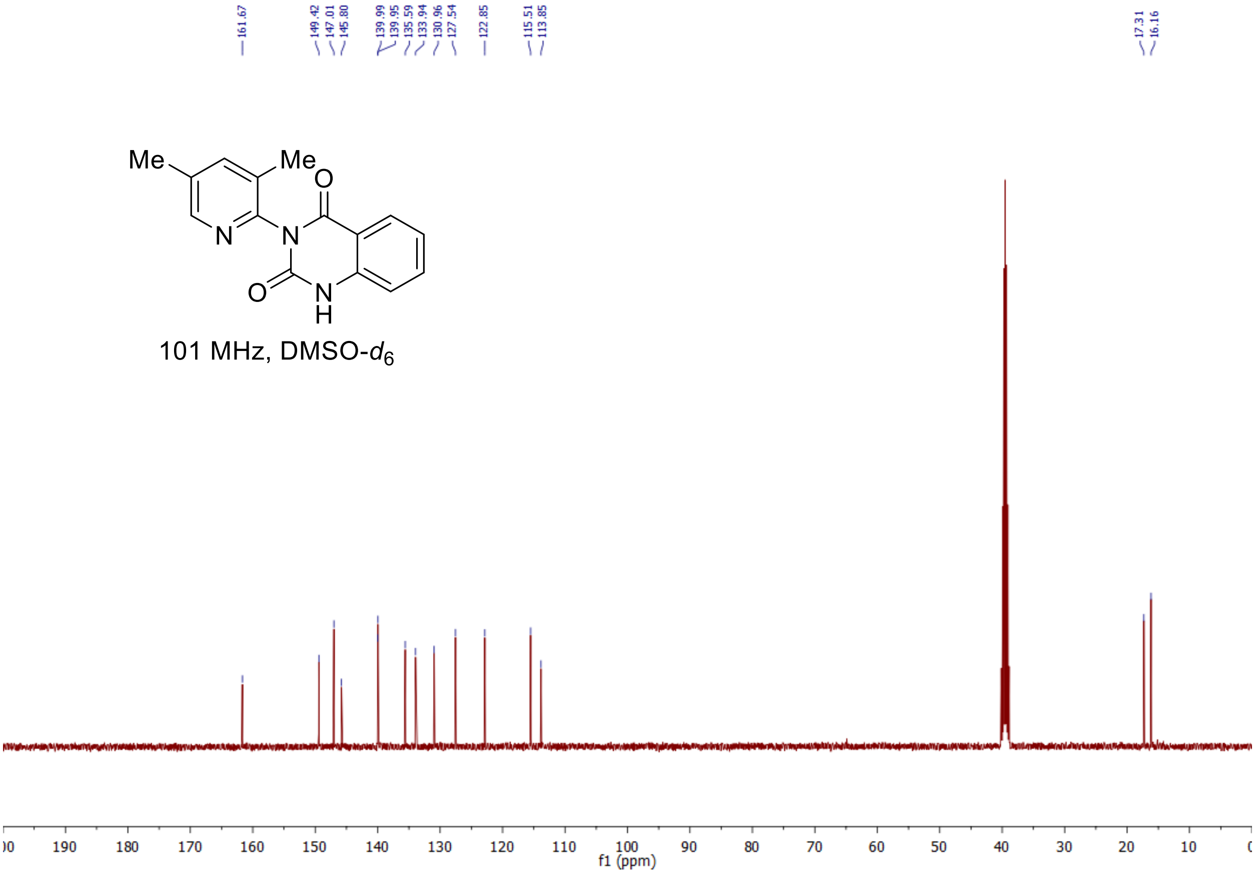

$^{1}H$ and $^{13}C$ spectra of 3-(4-methoxypyridin-2-yl)quinazoline-2,4(1*H*,3*H*)-dione **3f**

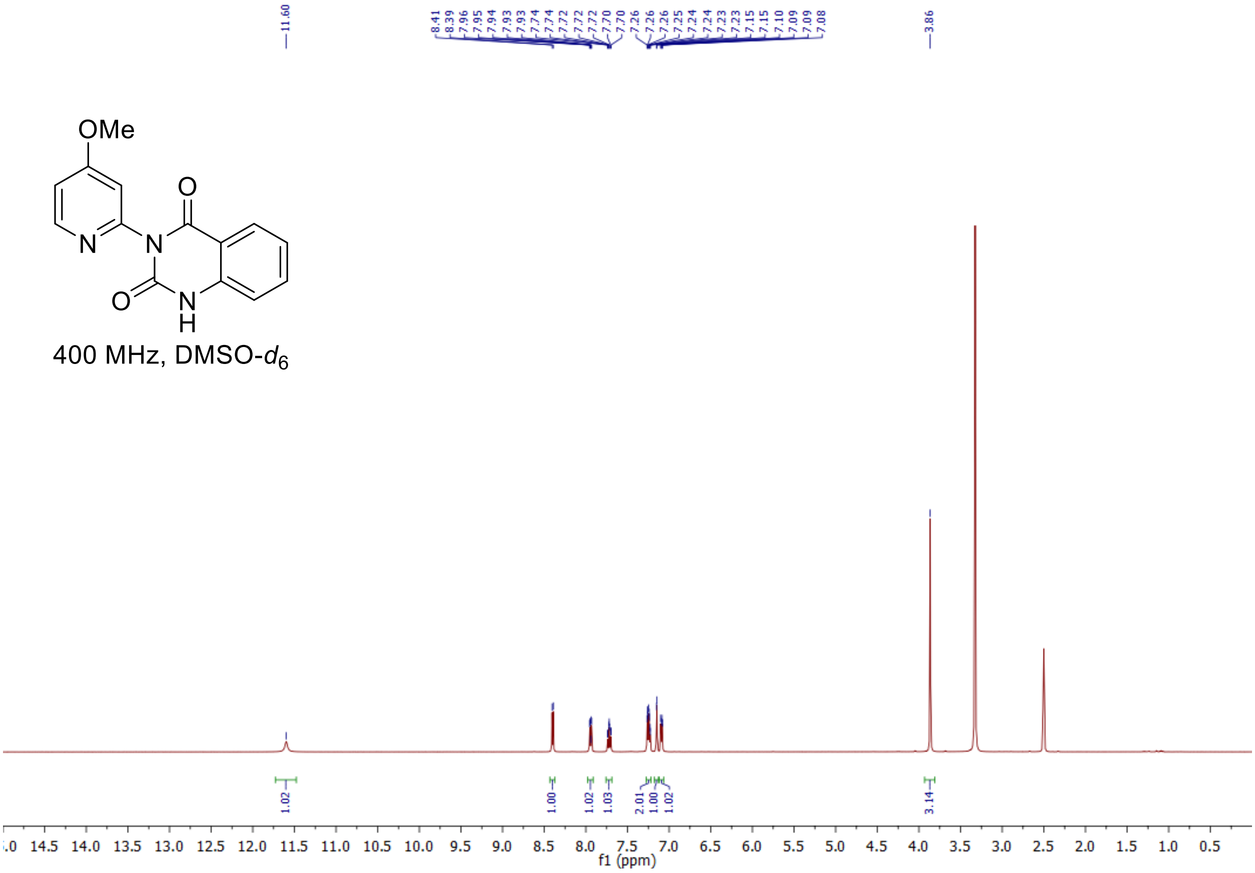


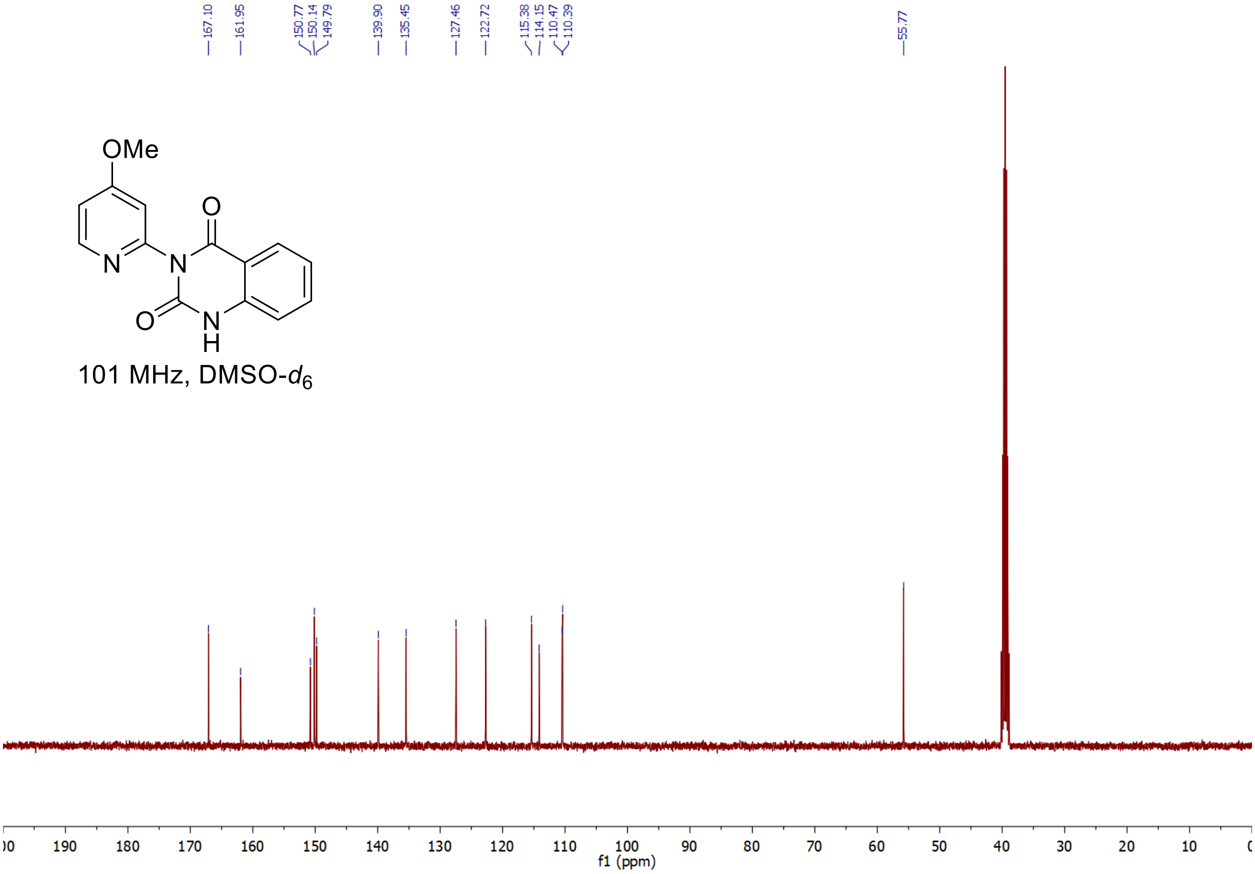

$^{1}$H and $^{13}$C spectra of 3-(6-phenylpyridin-2-yl)quinazoline-2,4(1*H*,3*H*)-dione **3g**

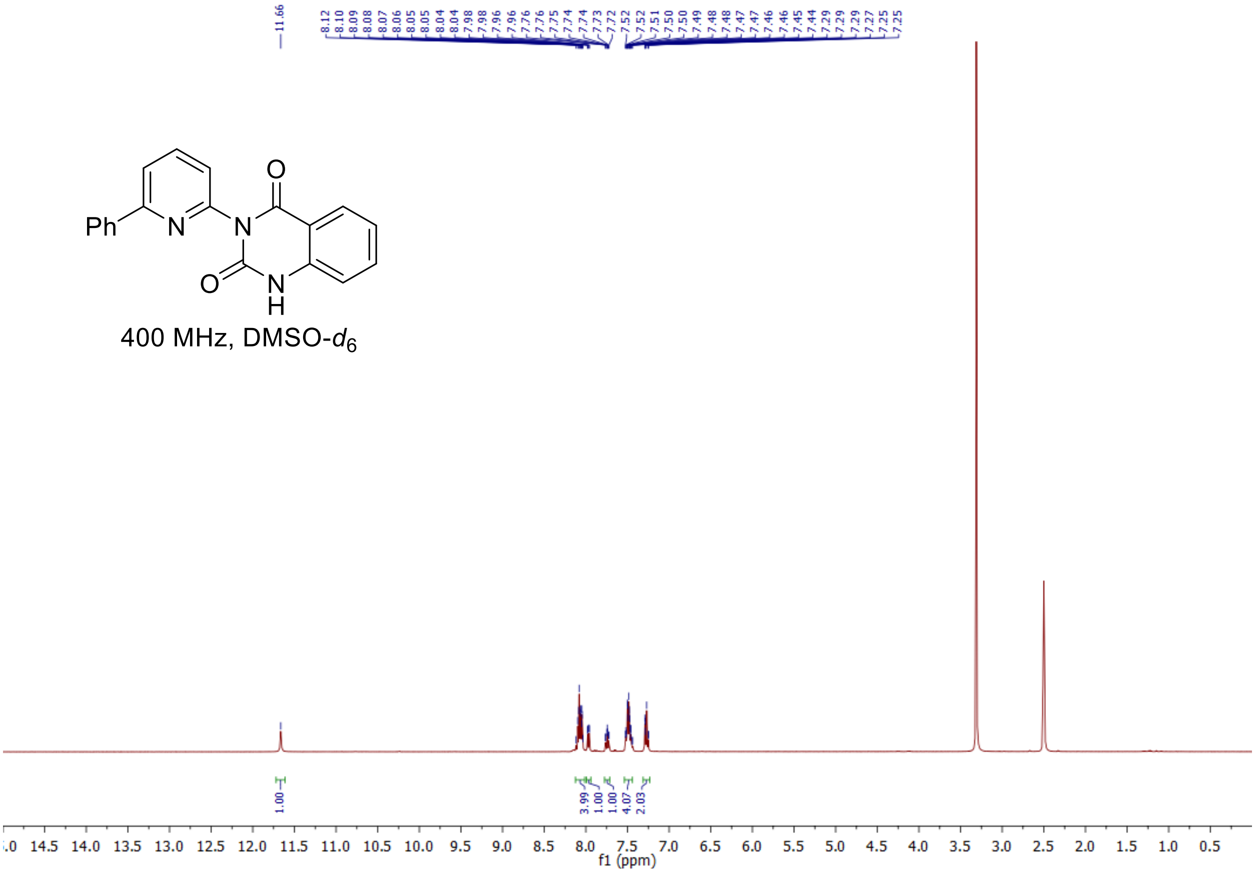


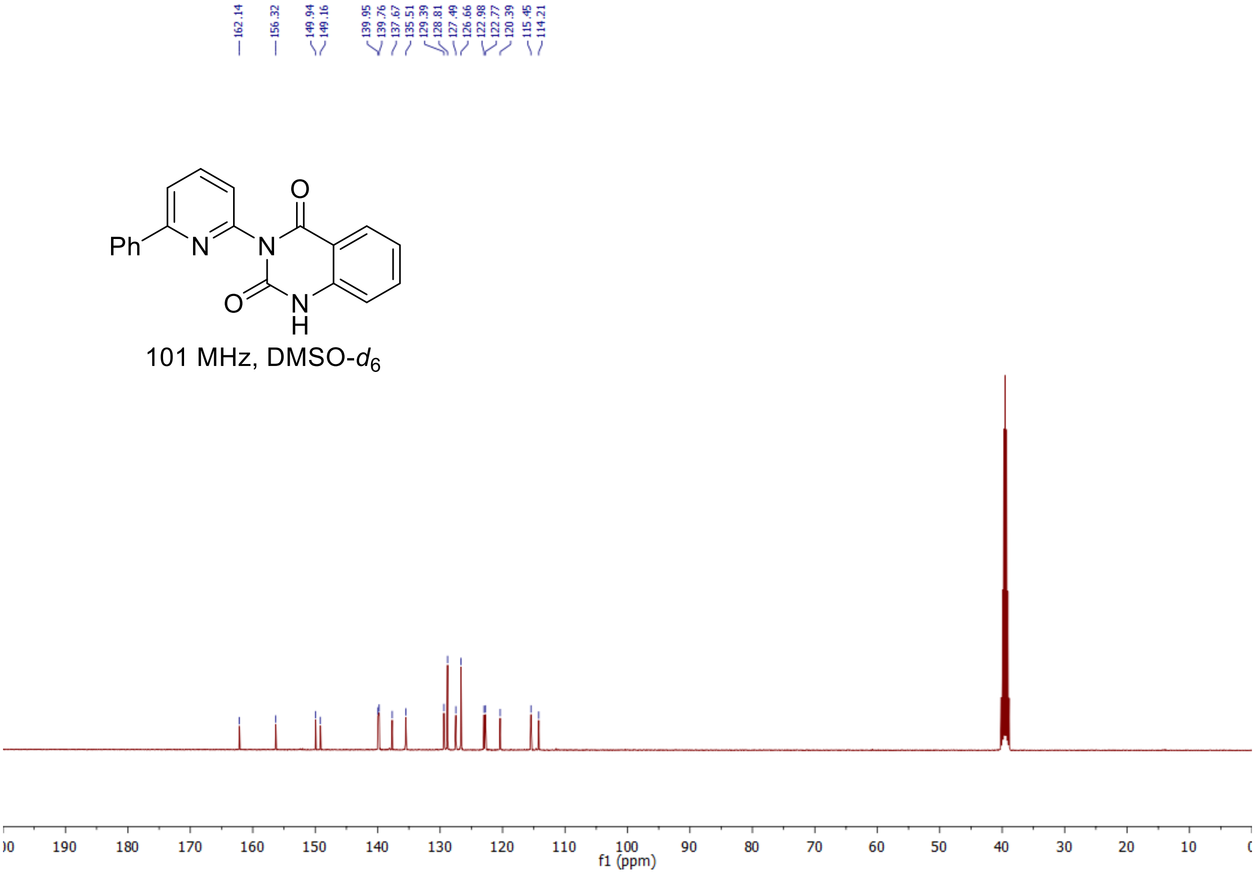

$^{1}$H and $^{13}$C spectra of methyl 6-(2,4-dioxo-1,4-dihydroquinazolin-3(2*H*)-yl)picolinate **3h**

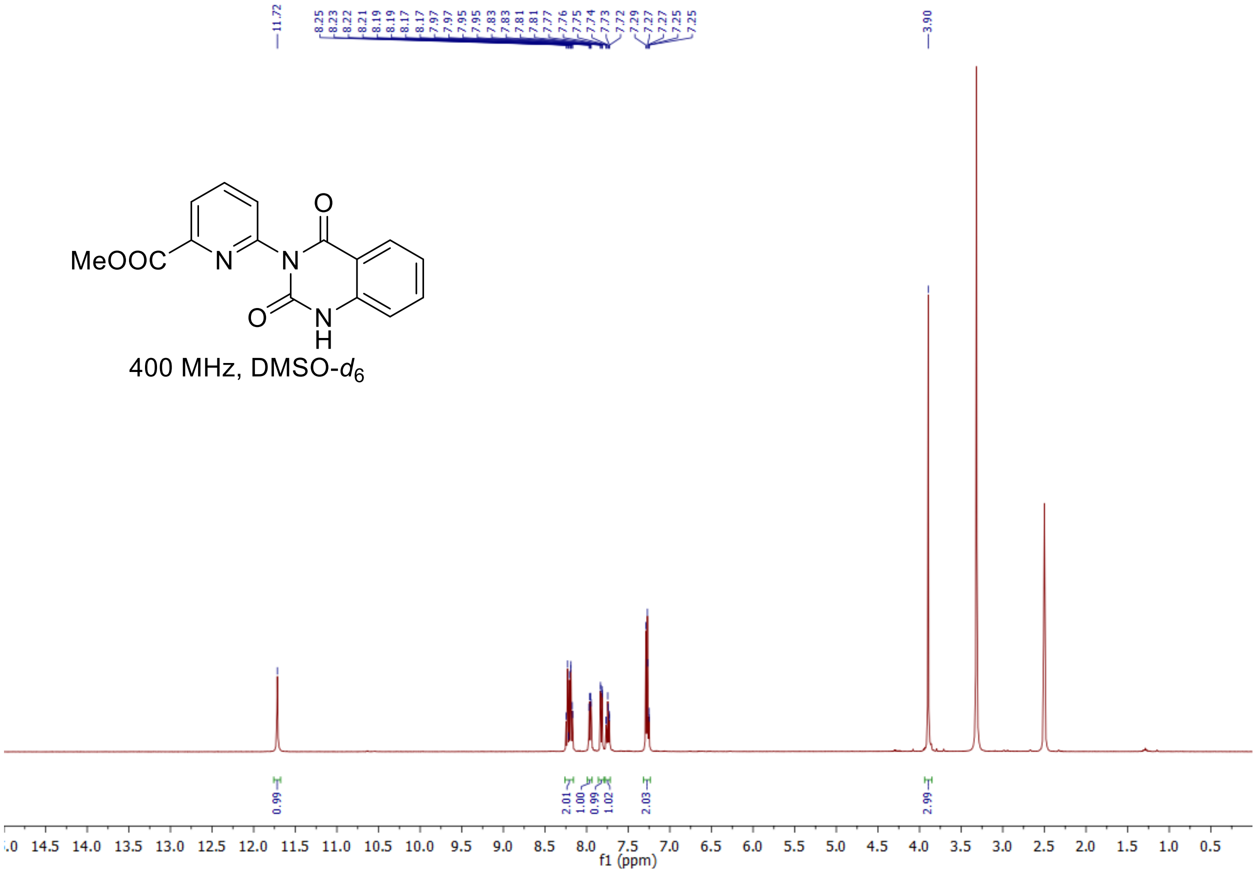


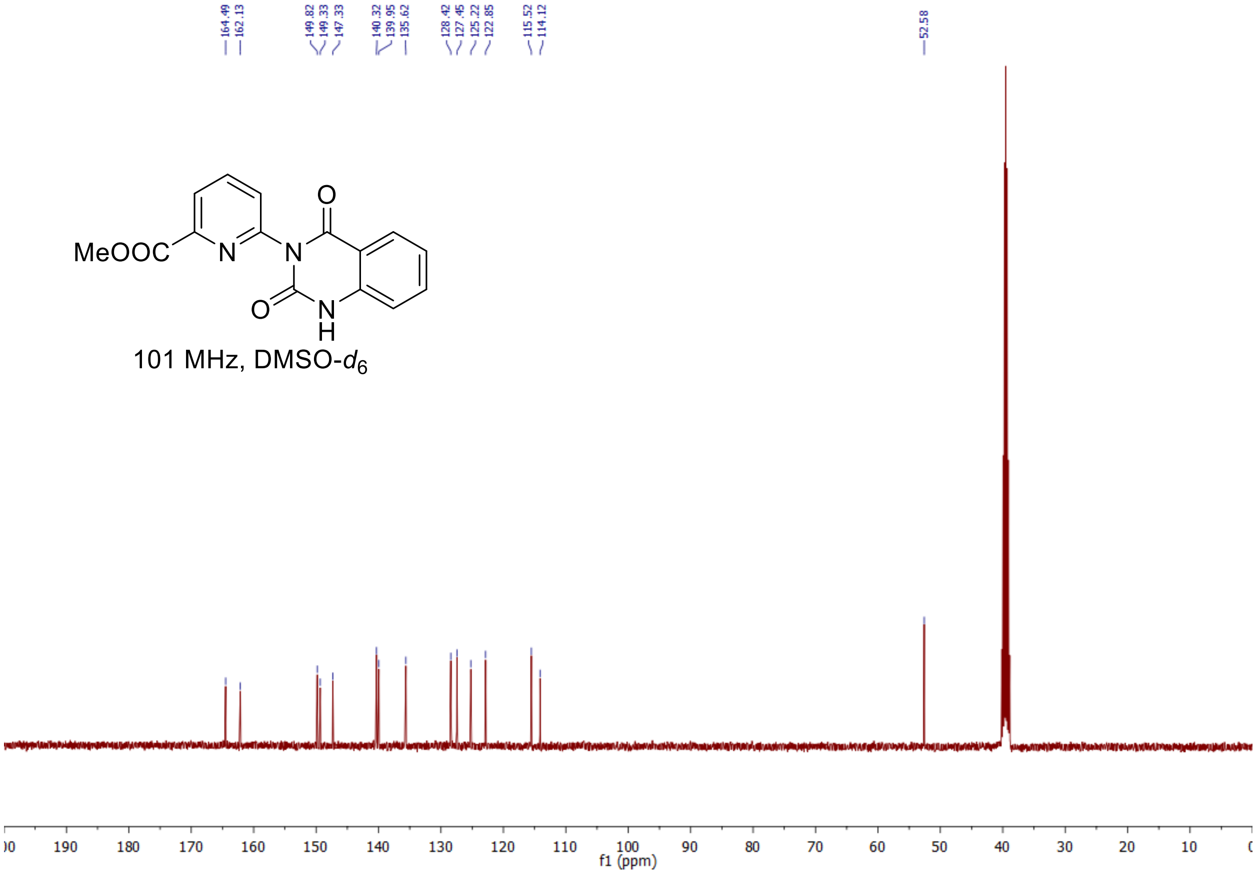

$^{1}$H and $^{13}$C spectra of methyl 2-(2,4-dioxo-1,4-dihydroquinazolin-3(2*H*)-yl)isonicotinate **3i**

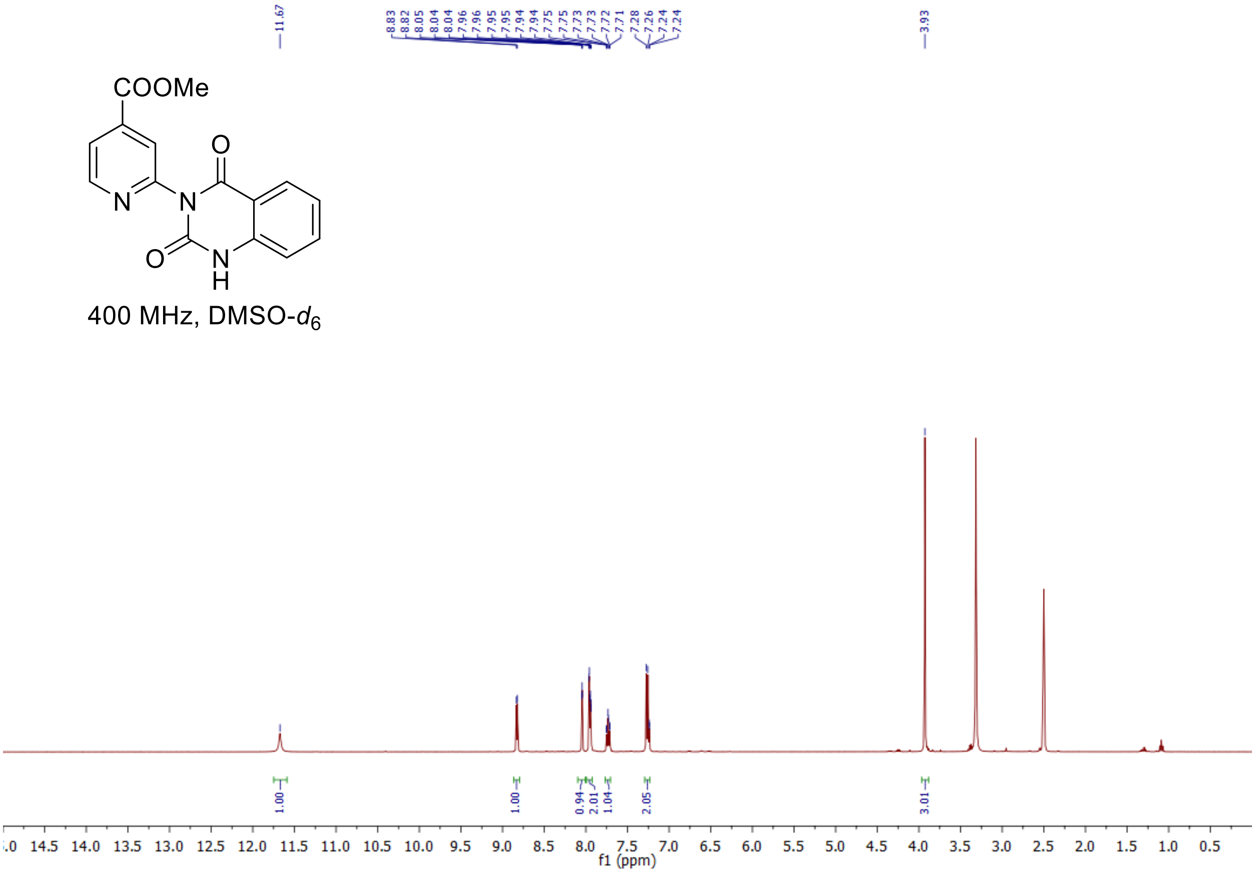


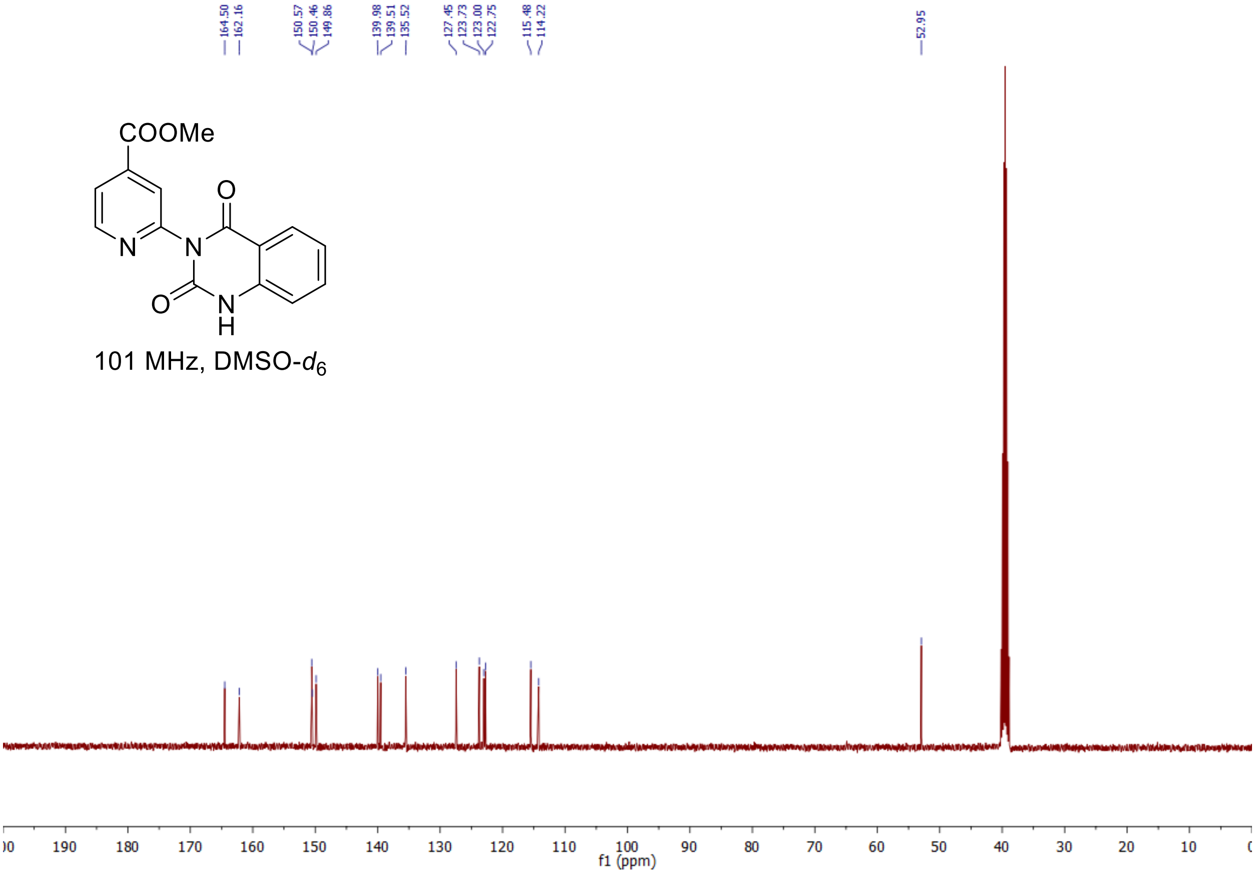

$^{1}$H and $^{13}$C spectra of 6-(2,4-dioxo-1,4-dihydroquinazolin-3(2*H*)-yl)picolinonitrile **3j**

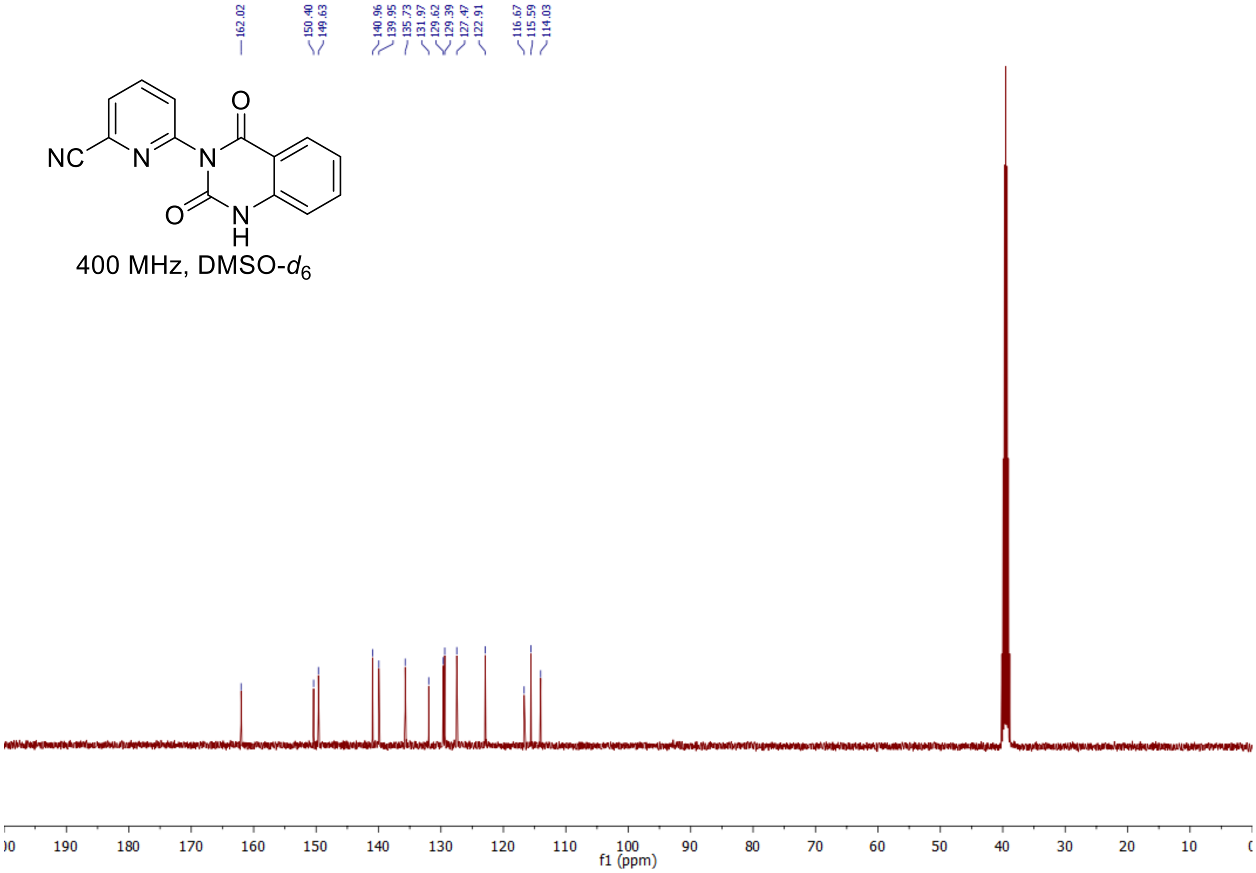


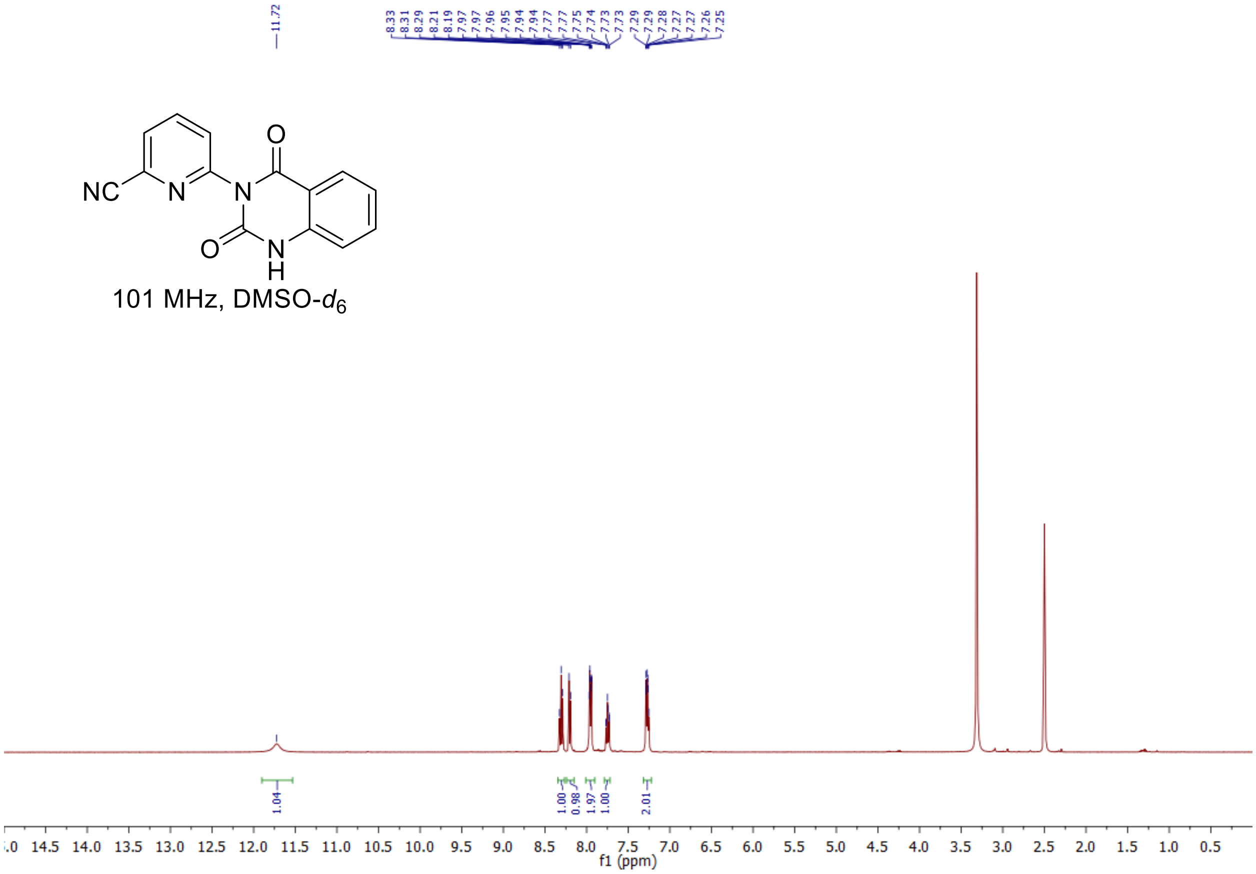

$^{1}$H and $^{13}$C spectra of 2-(2,4-dioxo-1,4-dihydroquinazolin-3(2*H*)-yl)isonicotinonitrile **3k**

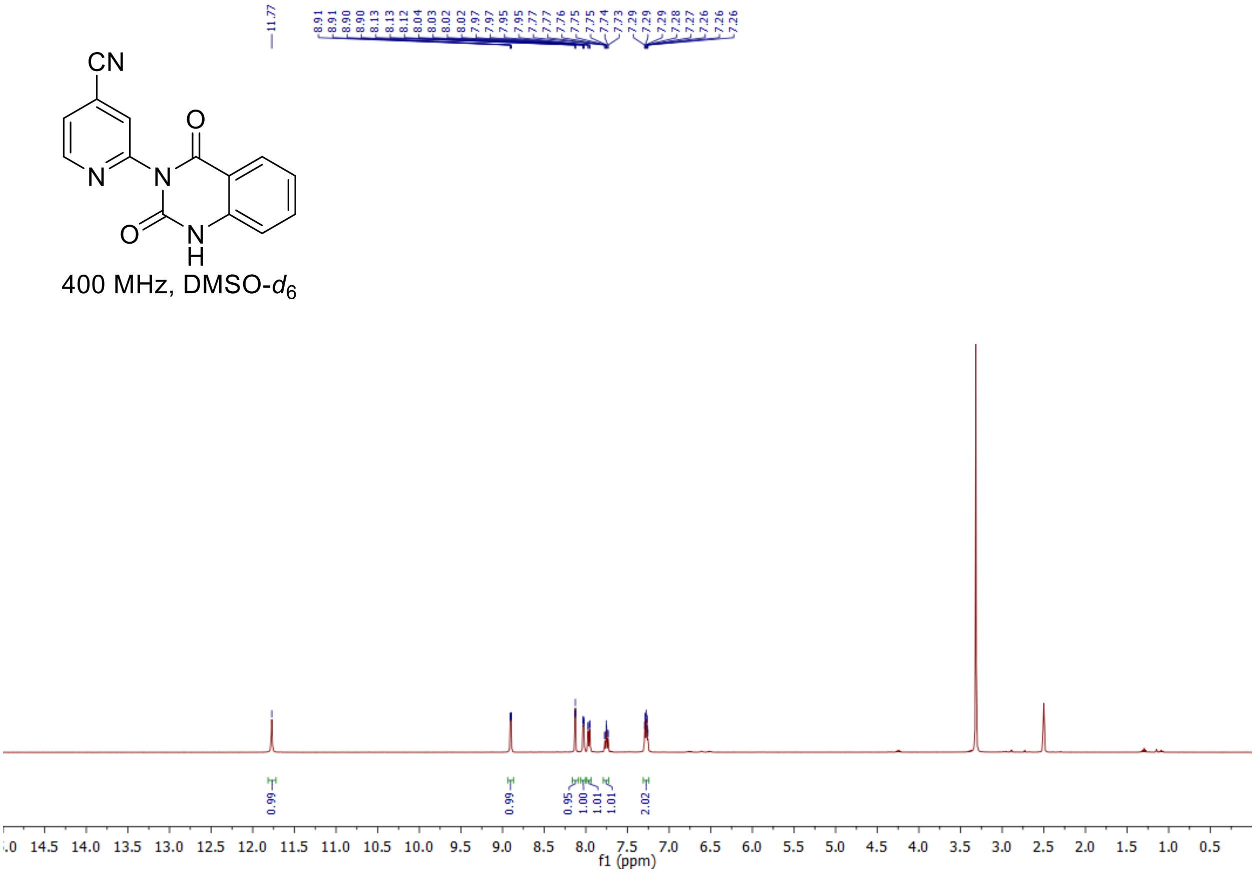


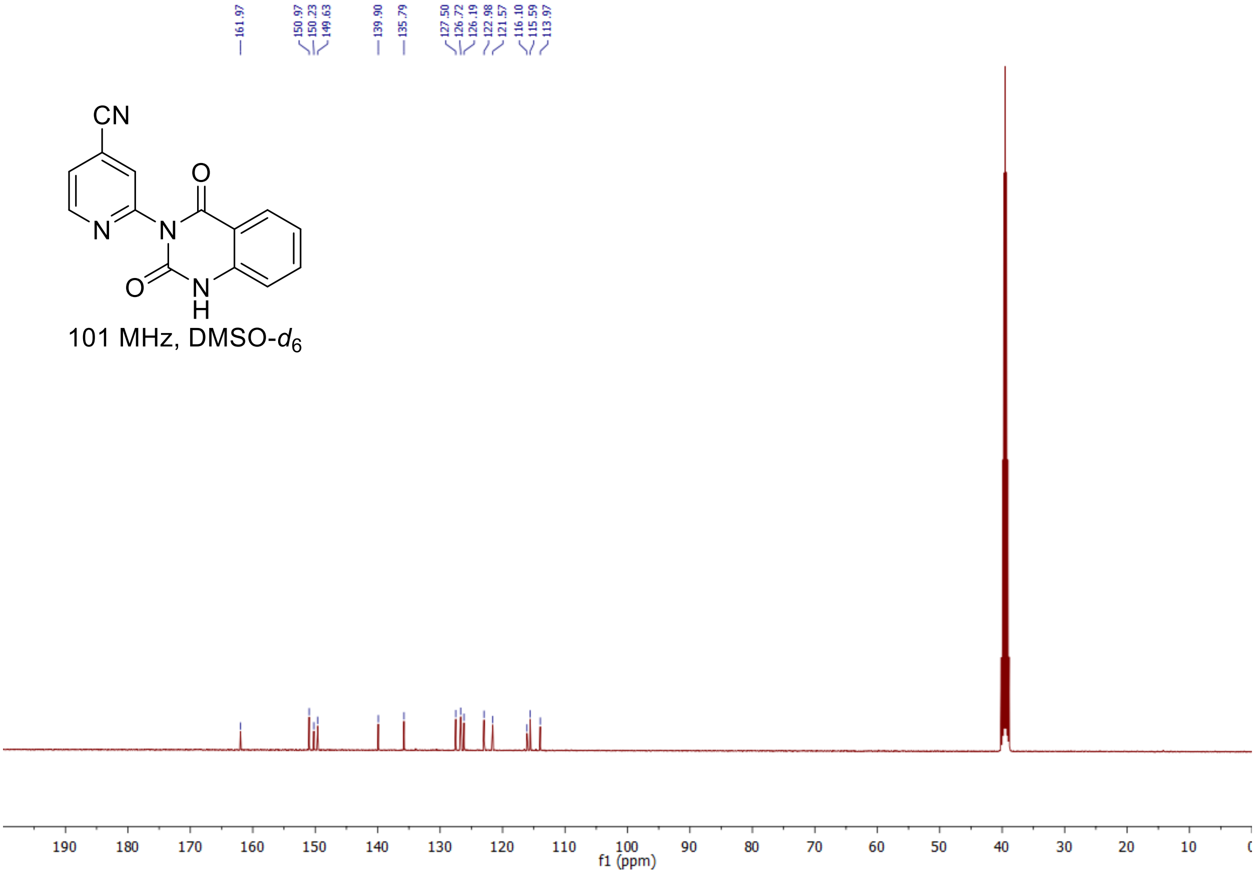

$^{1}H$ and $^{13}C$ spectra of 3-(quinolin-2-yl)quinazoline-2,4(1*H*,3*H*)-dione **3l**

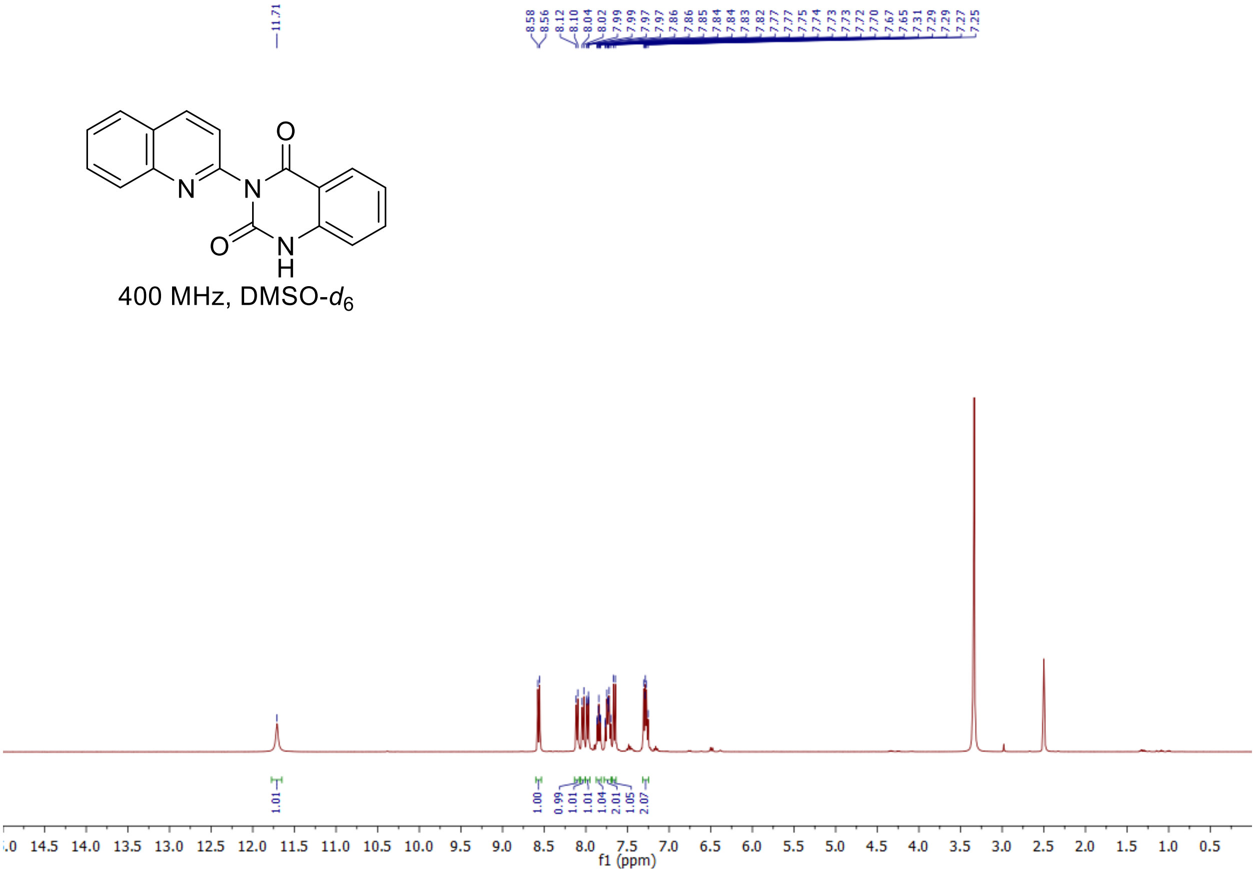


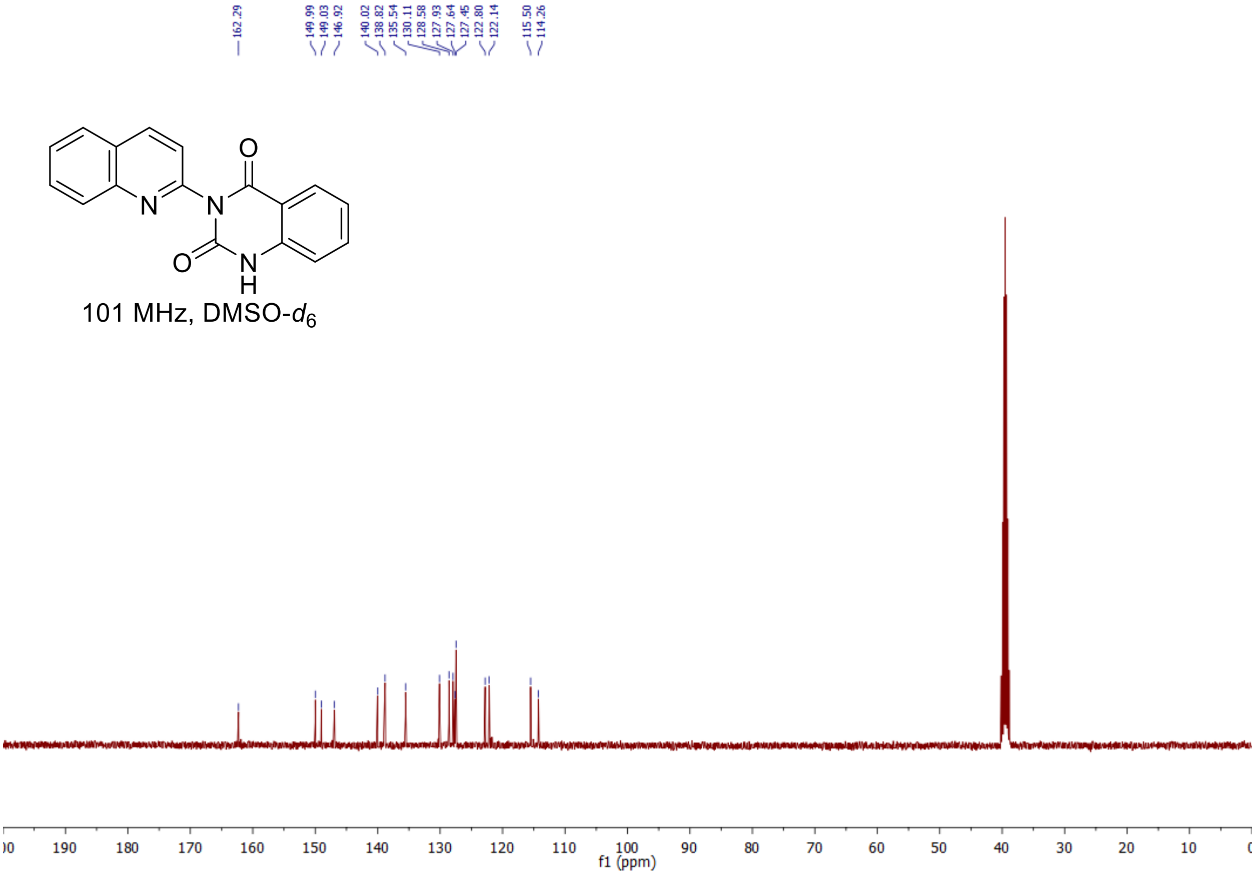

$^{1}$H and $^{13}$C spectra of 3-(4-methoxyquinolin-2-yl)quinazoline-2,4(1*H*,3*H*)-dione **3m**

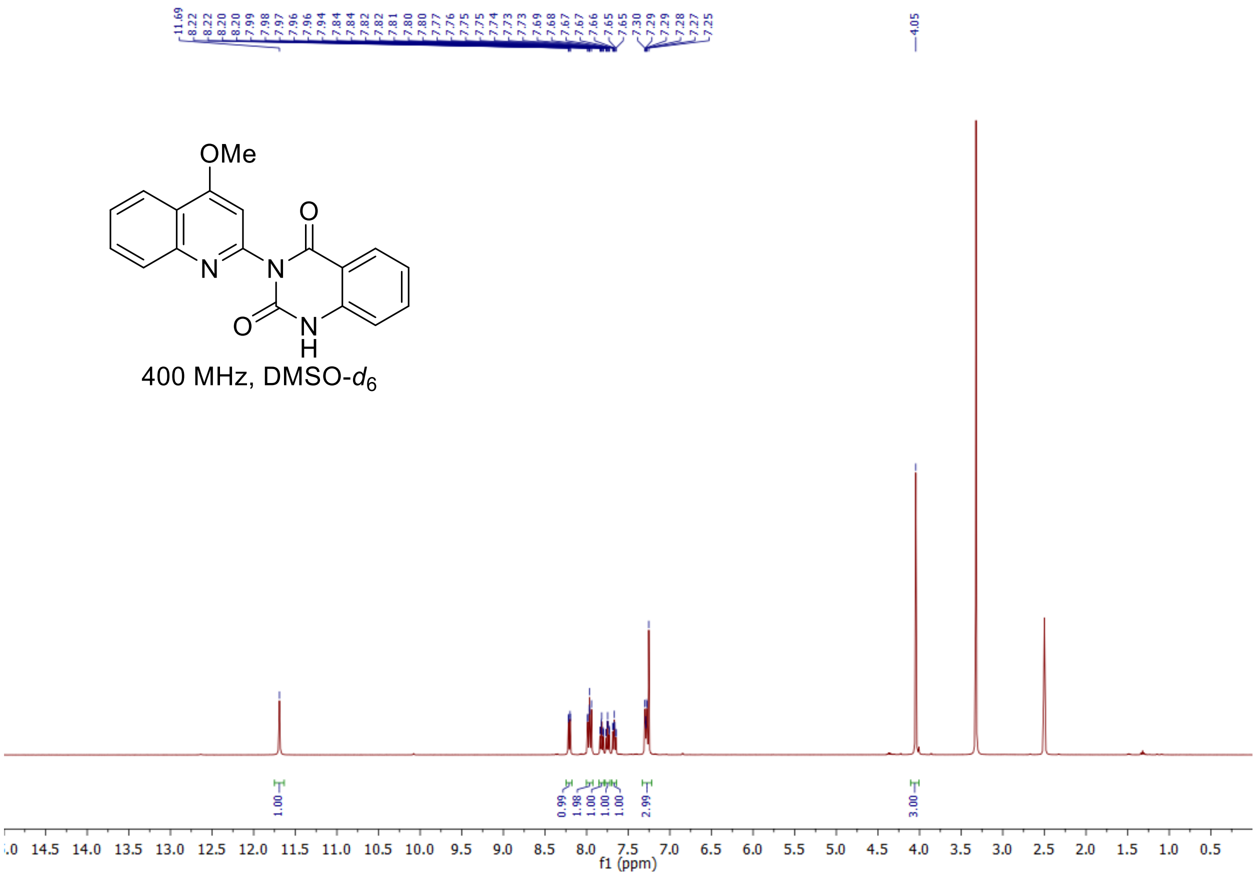


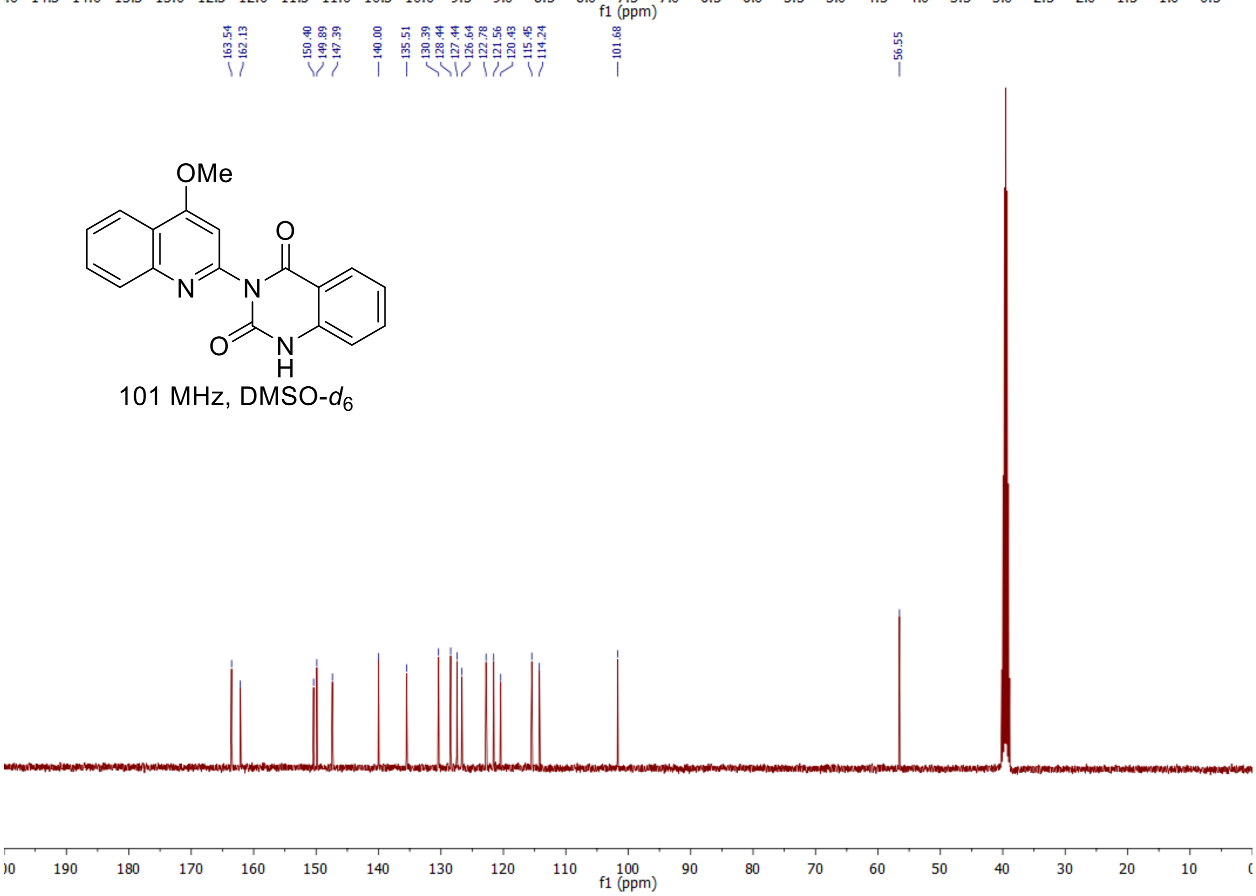

$^{1}$H and $^{13}$C spectra of 3-(6-methylquinolin-2-yl)quinazoline-2,4(1*H*,3*H*)-dione **3n**

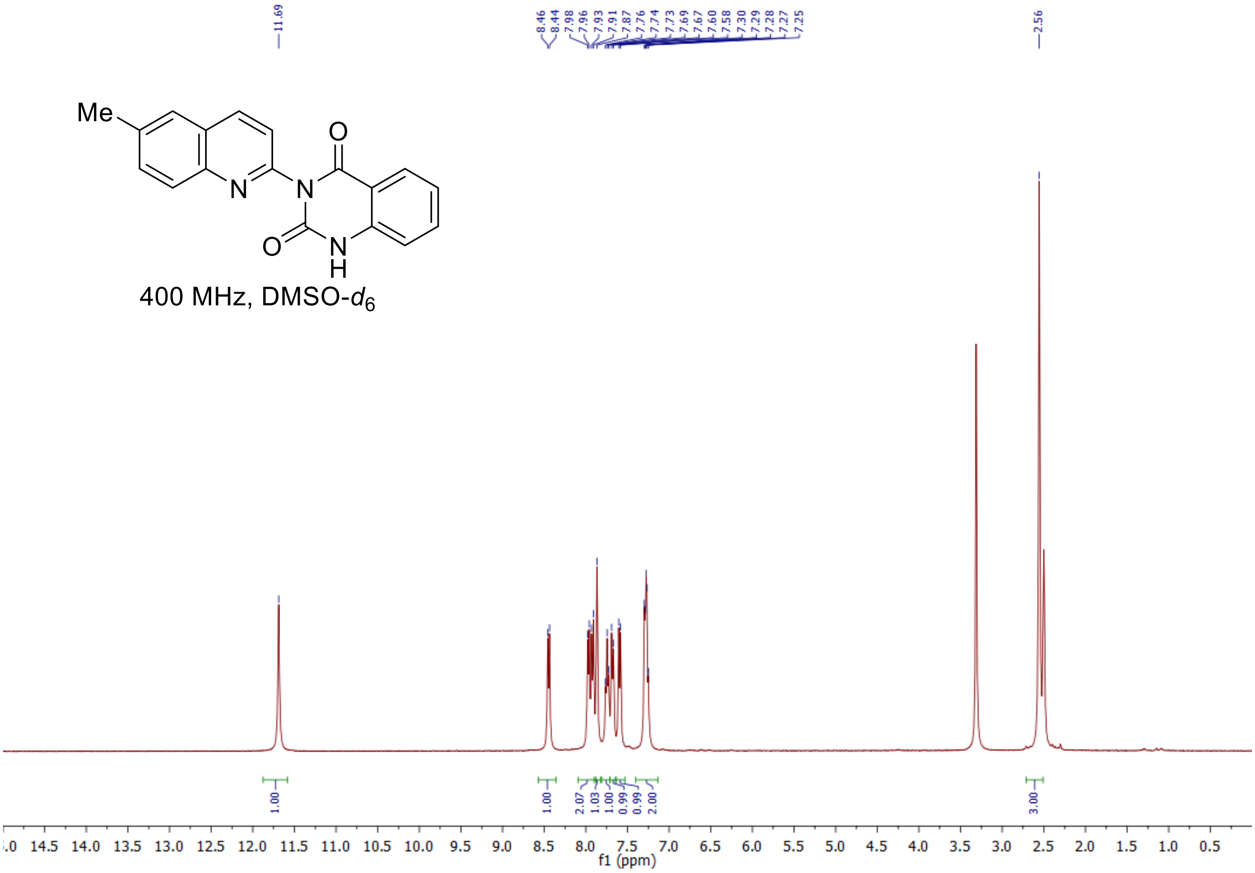


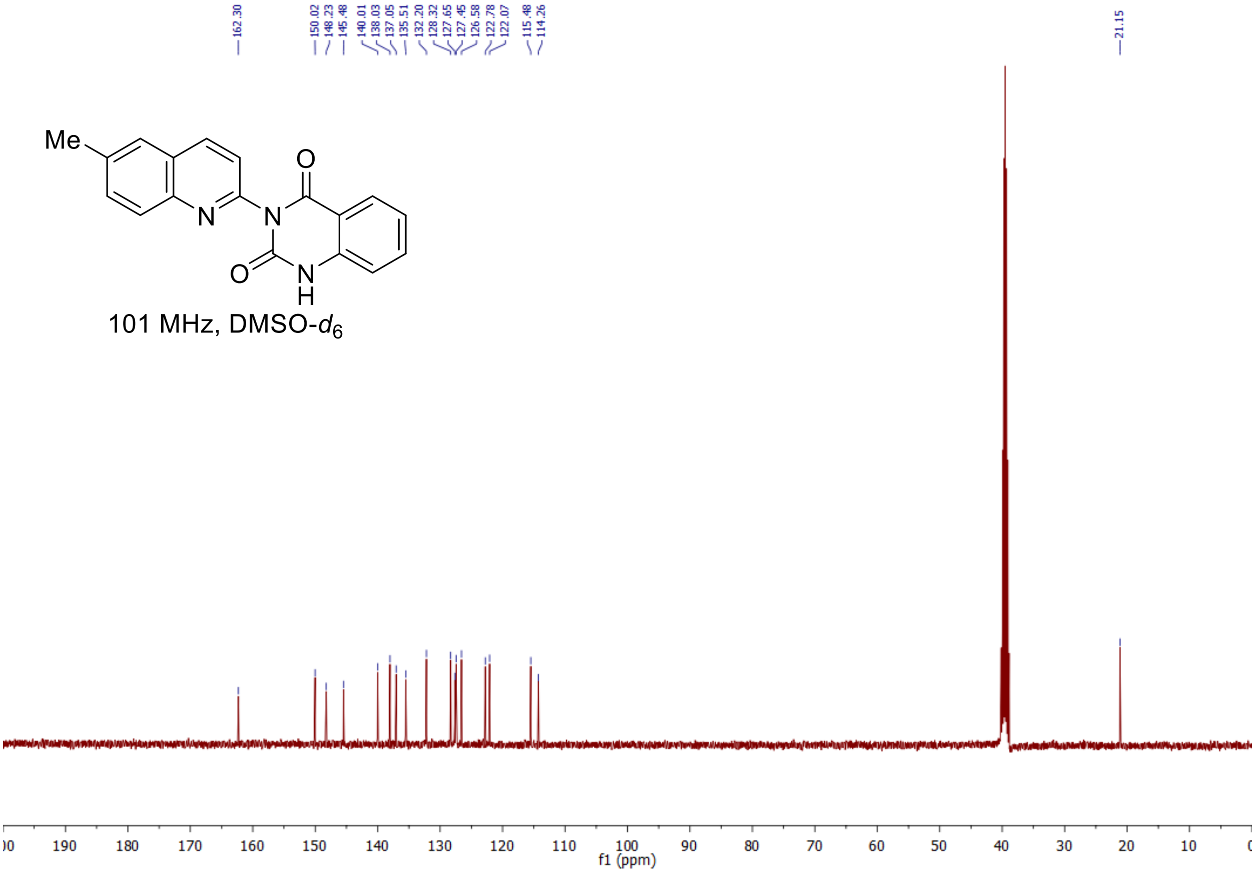

$^{1}H$ and $^{13}C$ spectra of 3-(7-methylquinolin-2-yl)quinazoline-2,4(1*H*,3*H*)-dione **3o**

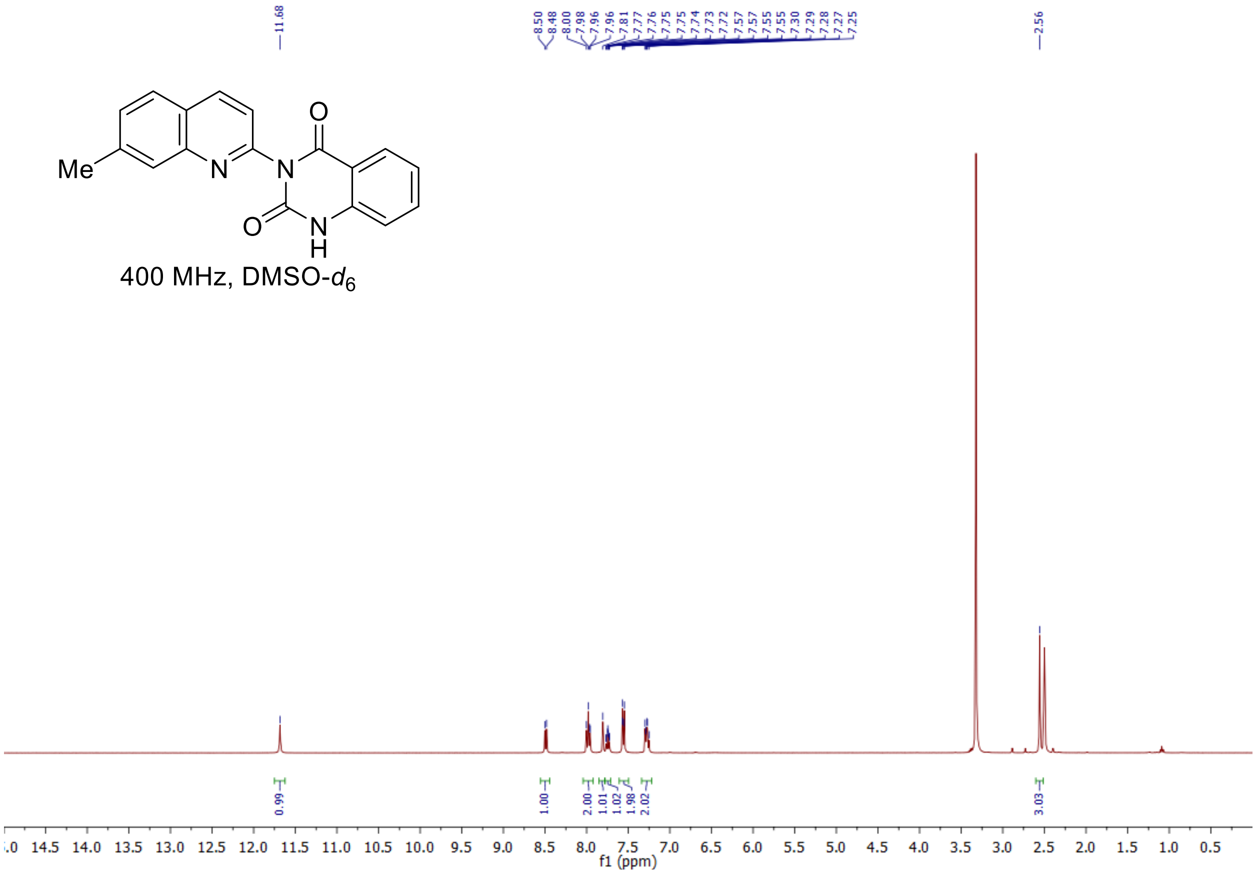


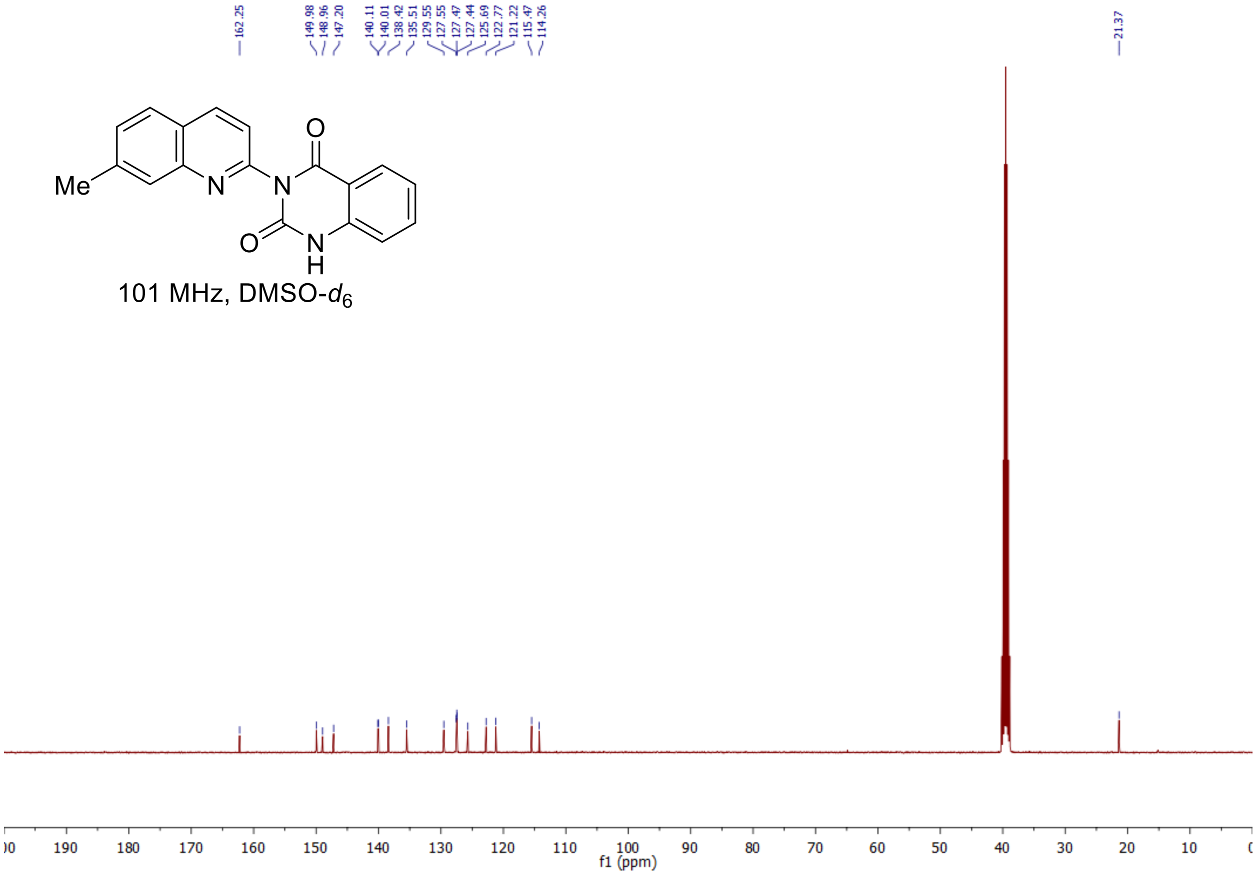

$^{1}H$ and $^{13}C$ spectra of 3-(6-methoxyquinolin-2-yl)quinazoline-2,4(1*H*,3*H*)-dione **3p**

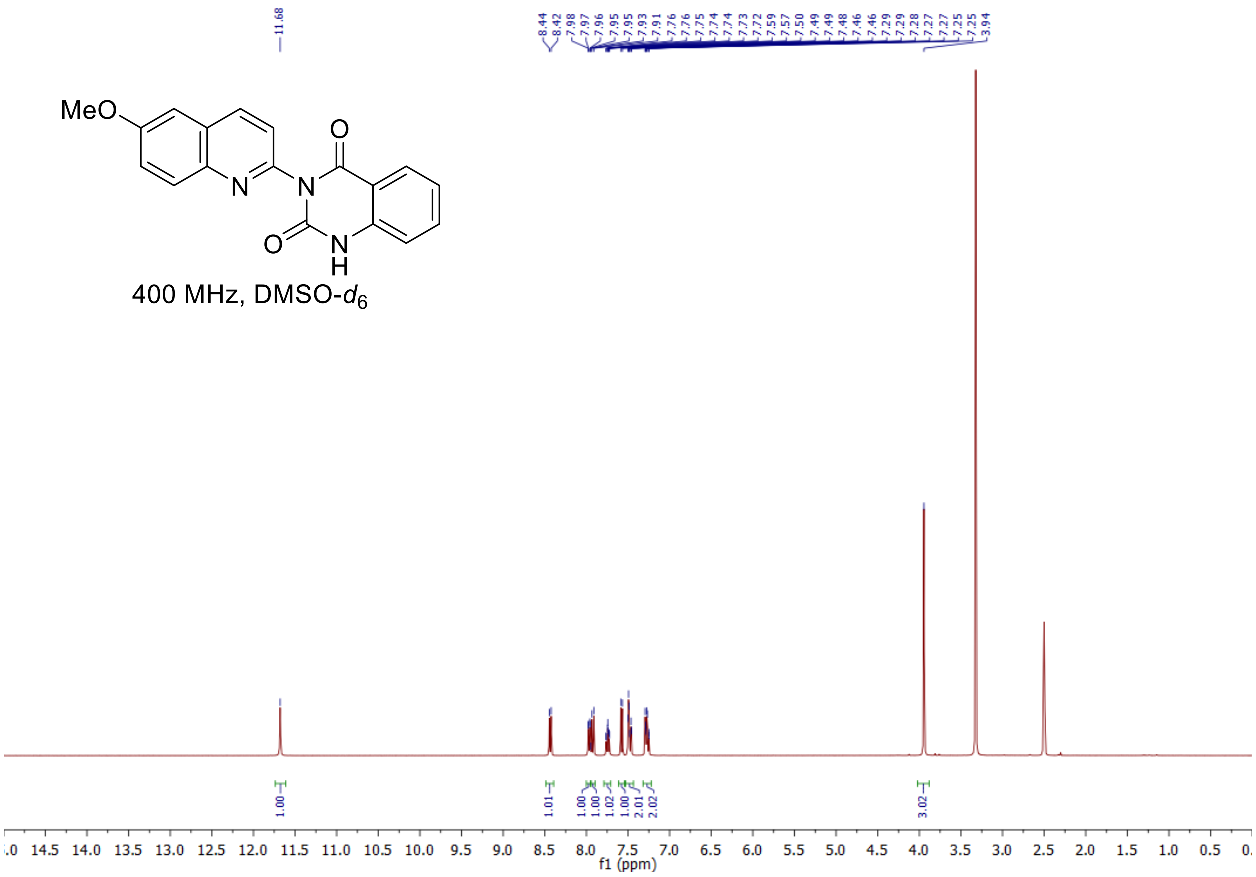


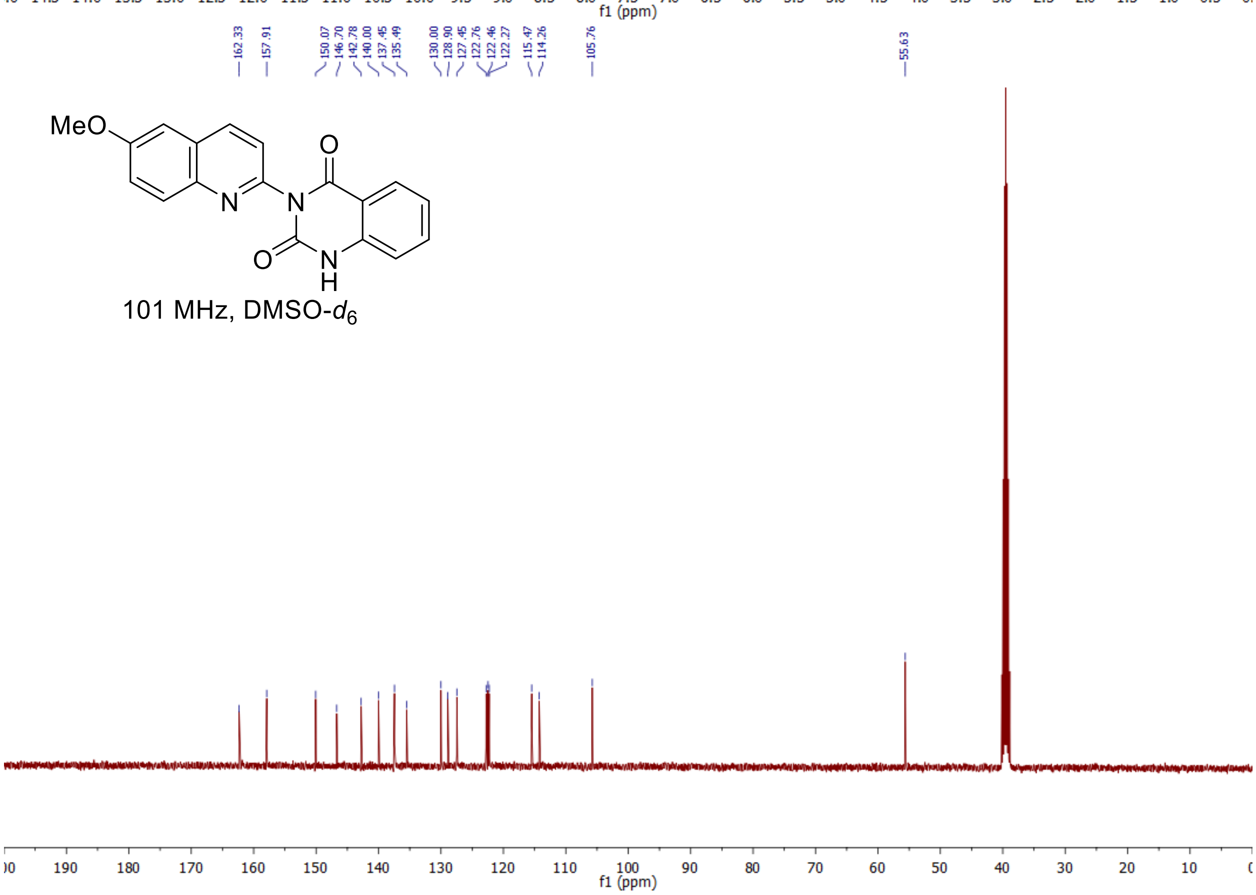

$^{1}$H and $^{13}$C spectra of 3-(isoquinolin-1-yl)quinazoline-2,4(1*H*,3*H*)-dione **3q**

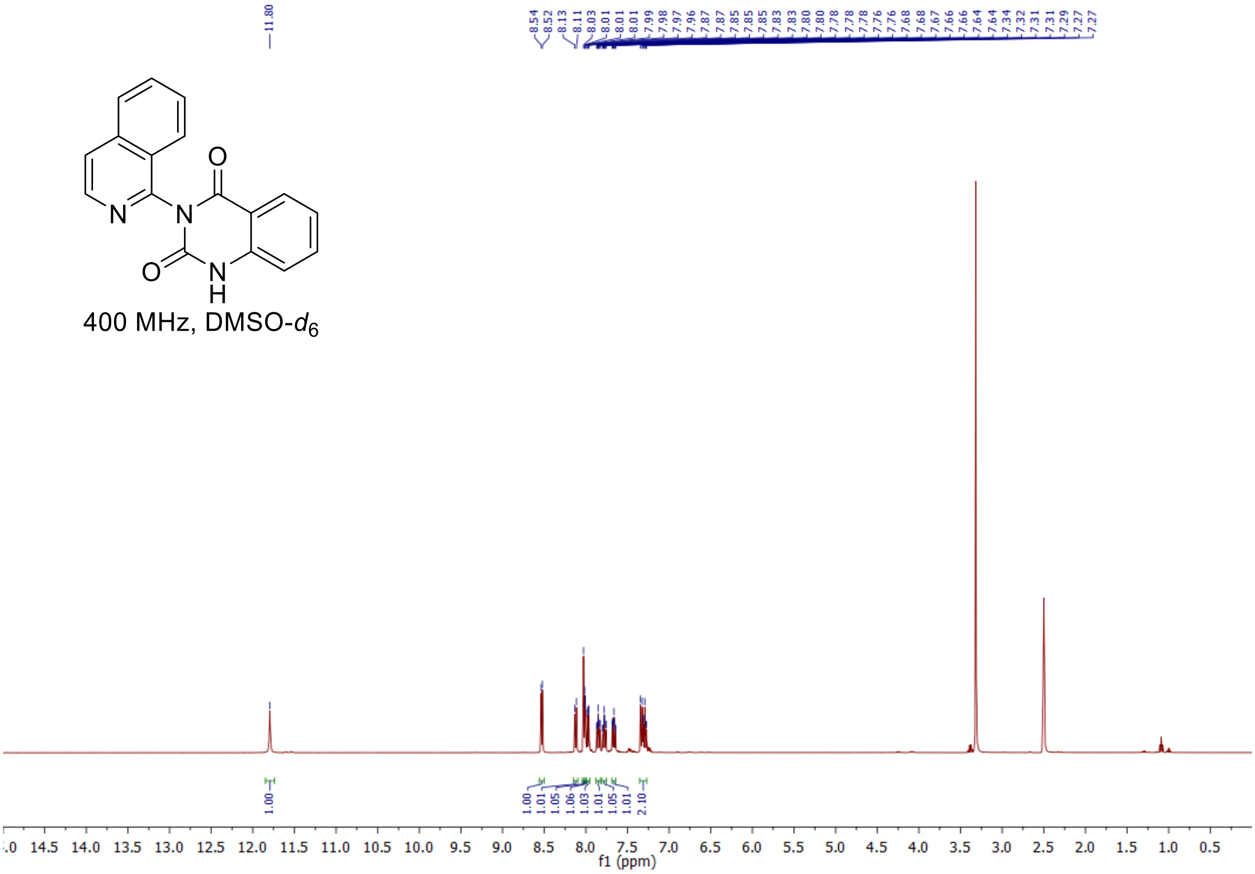


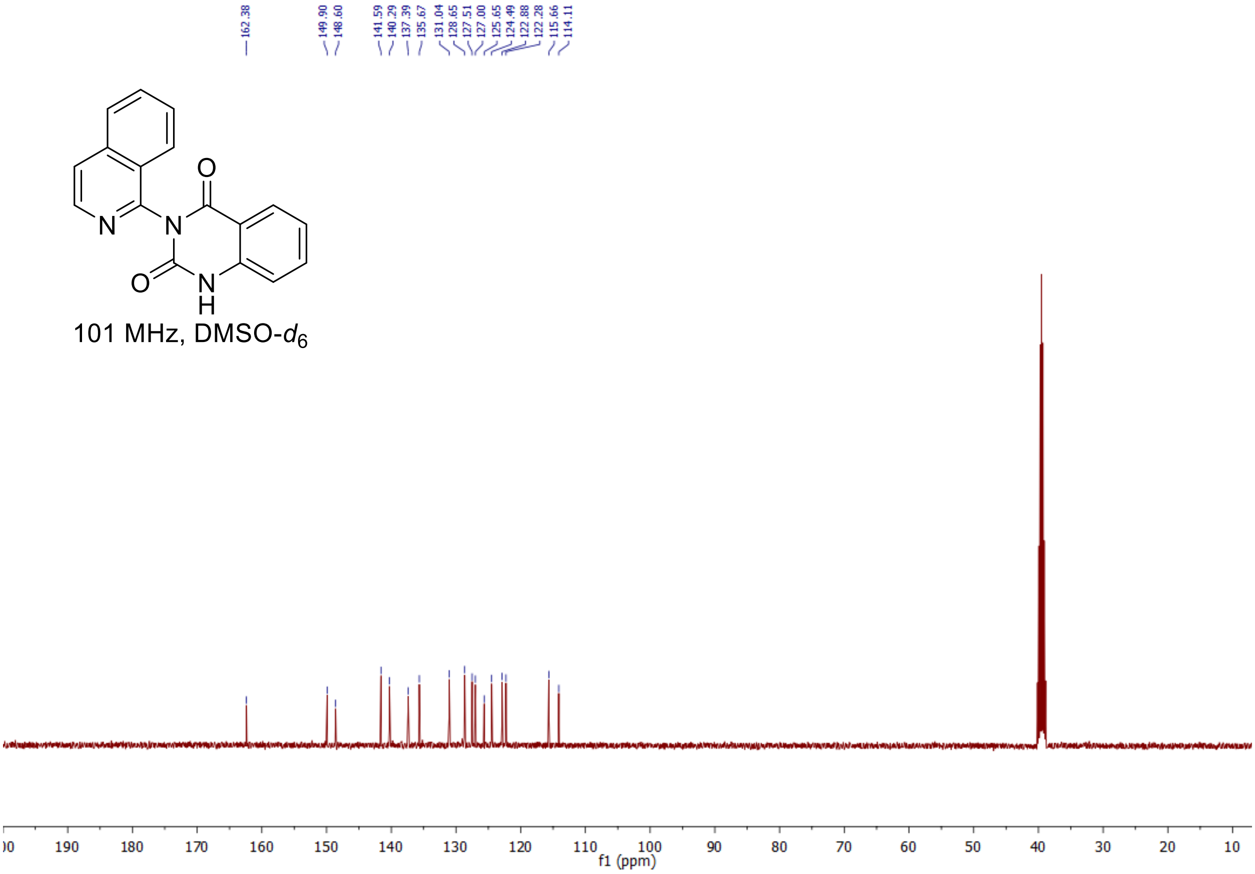

$^1H$ and $^{13}C$ spectra of 6-methyl-3-(4-methylpyridin-2-yl)quinazoline-2,4(1*H*,3*H*)-dione **3r**

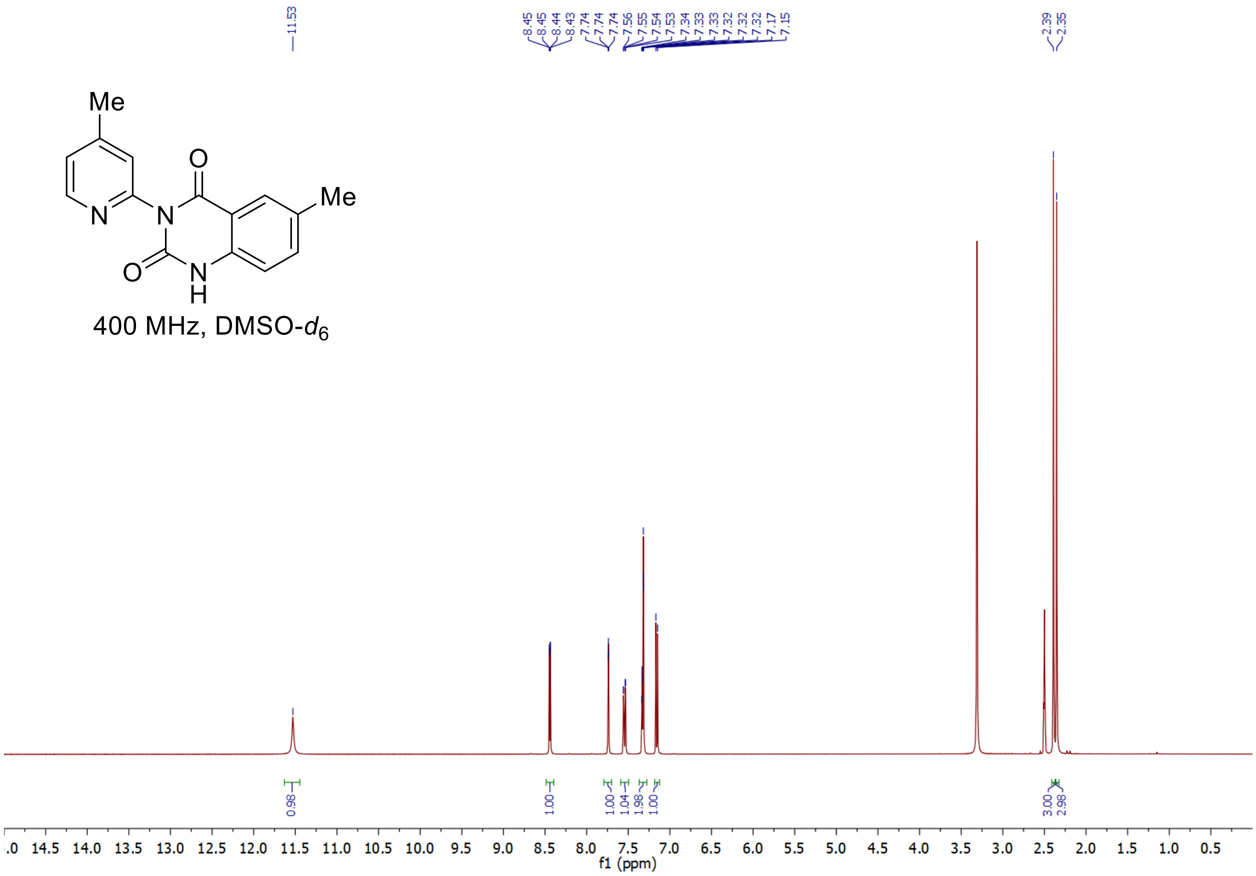


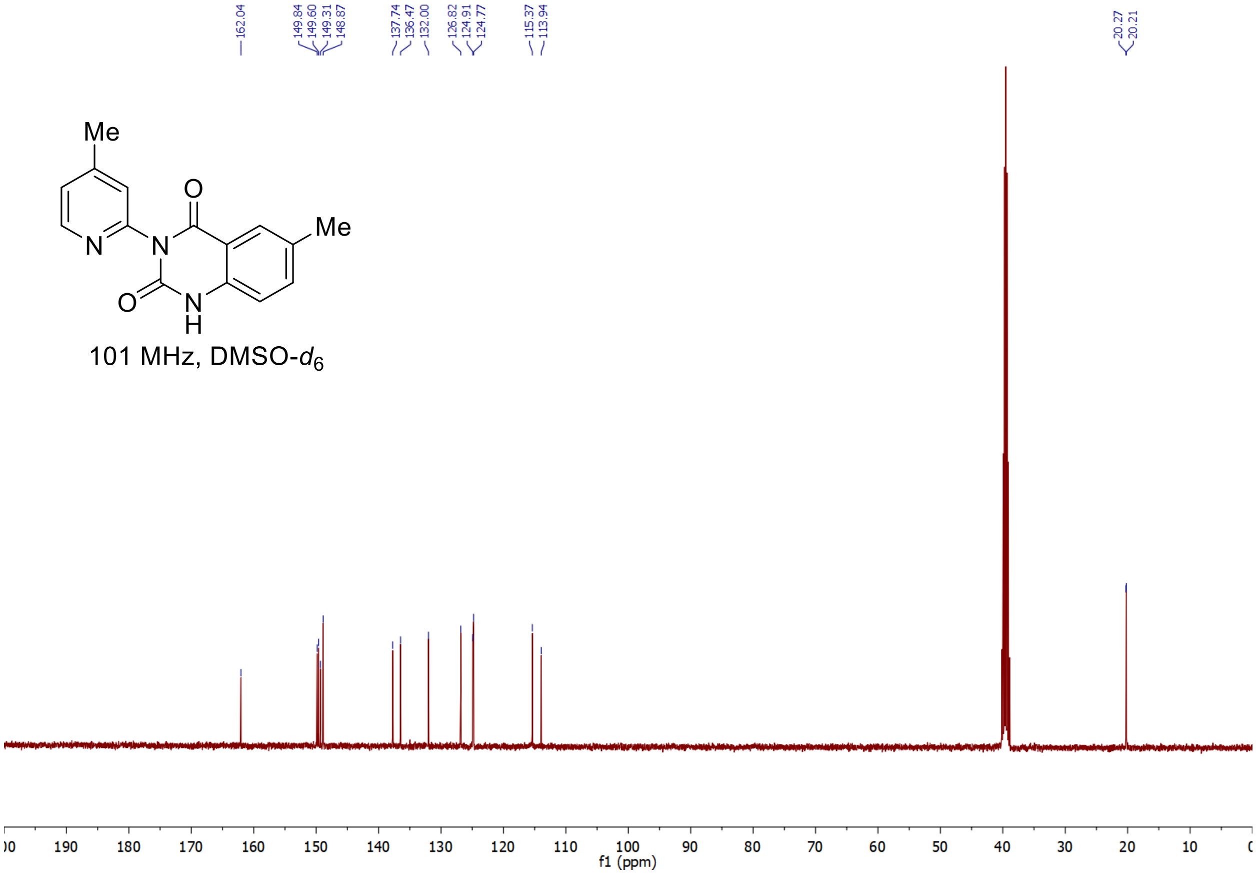

$^{1}$H and $^{13}$C spectra of 8-methyl-3-(4-methylpyridin-2-yl)quinazoline-2,4(1*H*,3*H*)-dione **3s**

.

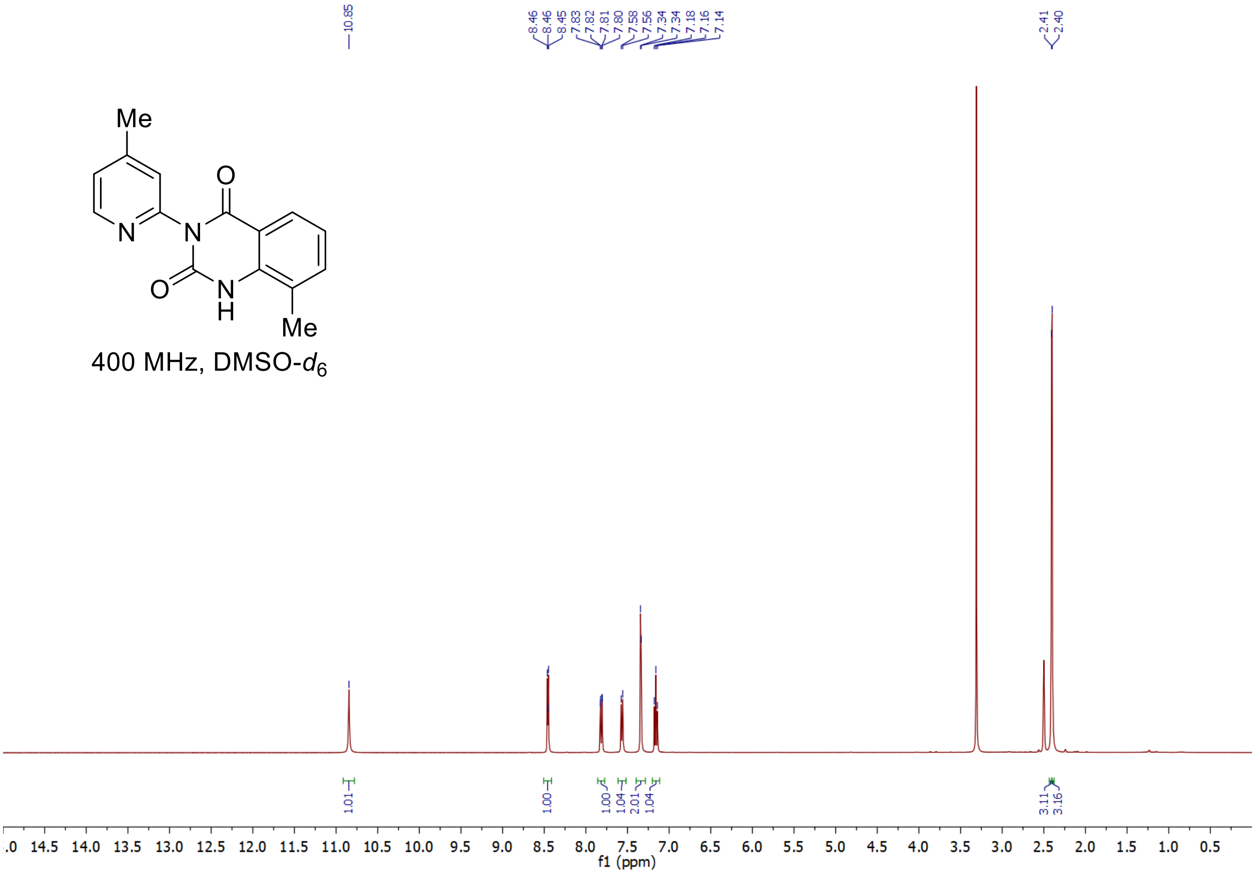


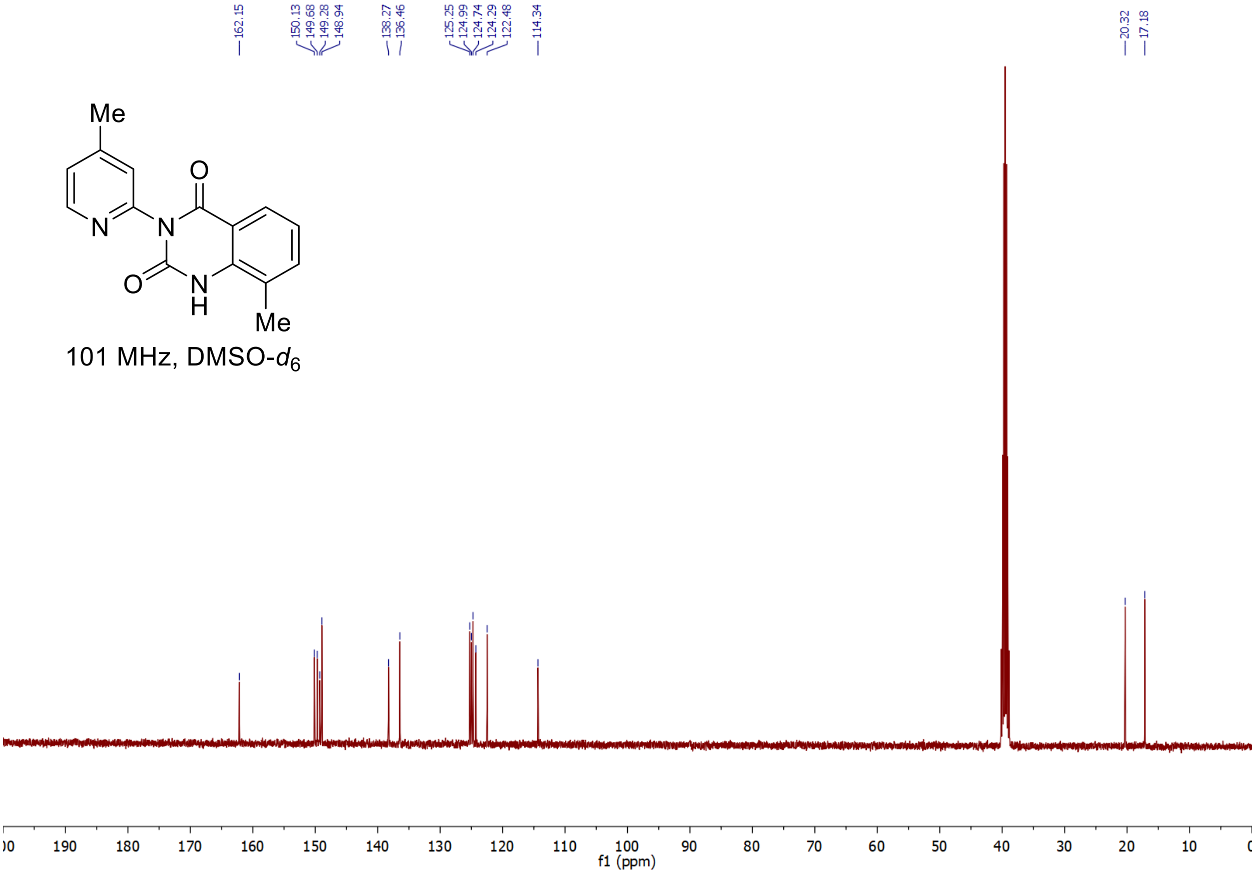

$^{1}H$,$^{13}C$, and $^{19}F$spectra of 6-fluoro-3-(4-methylpyridin-2-yl)quinazoline-2,4(1*H*,3*H*)-dione **3t**

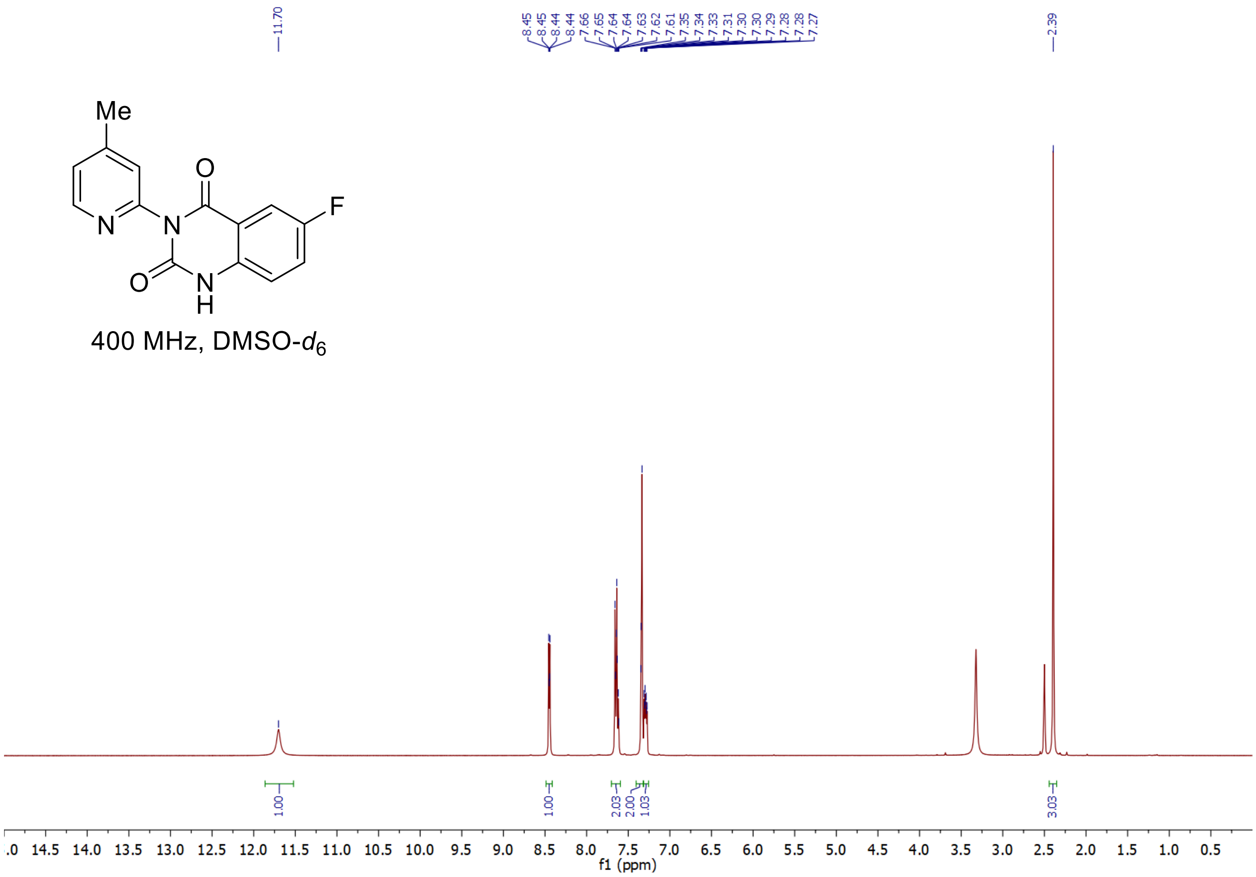


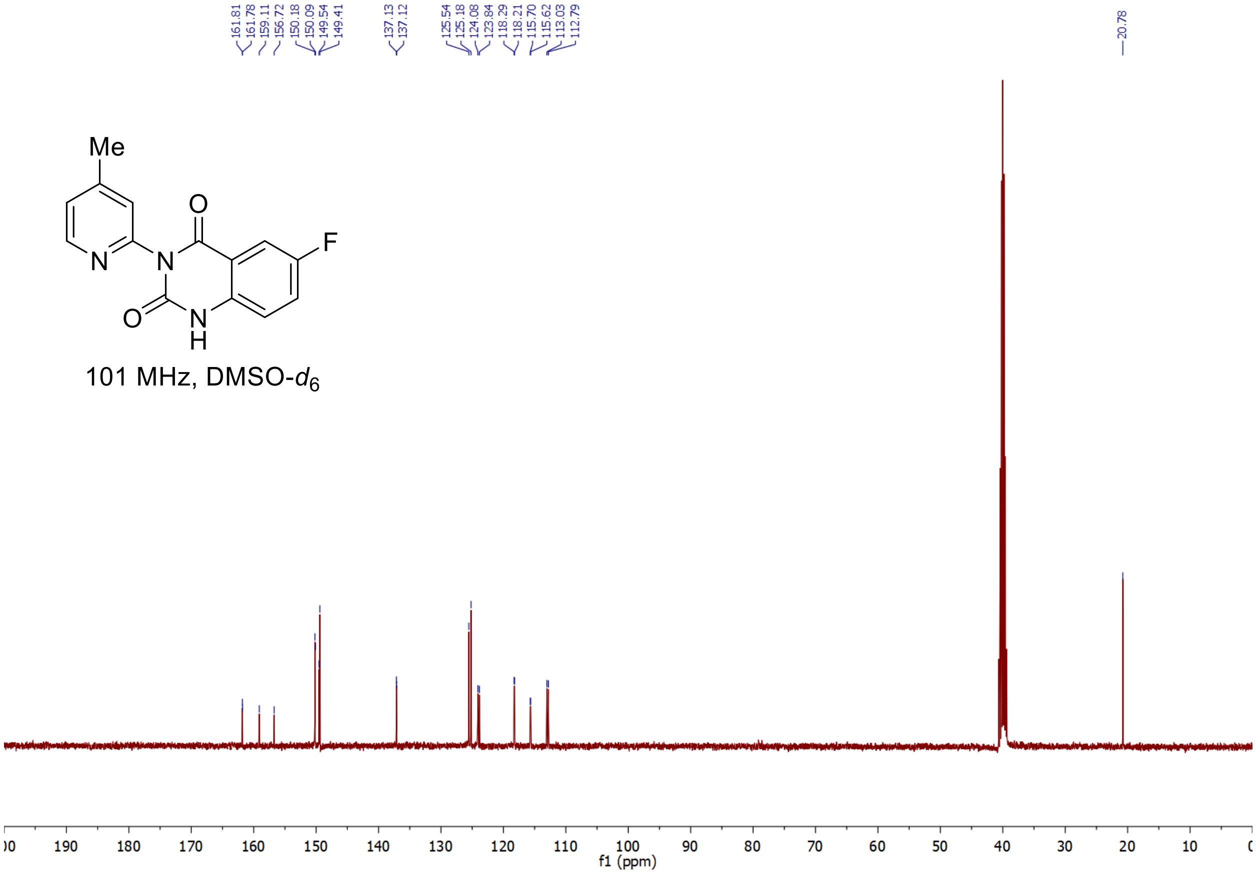

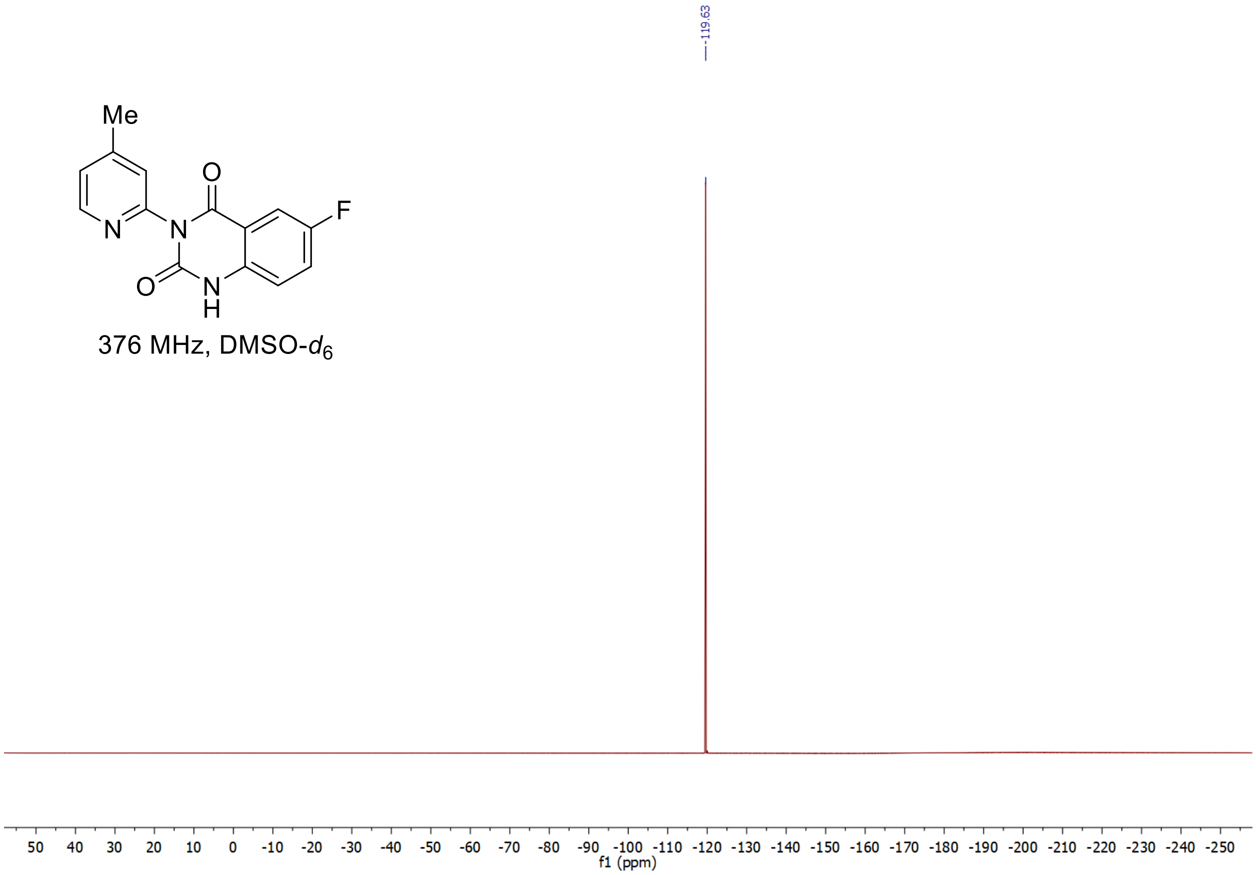
-119.63
Me
O
N
N
F
O
N
H
376 MHz, DMSO-$d_6$
50 40 30 20 10 0 -10 -20 -30 -40 -50 -60 -70 -80 -90 -100 -110 -120 -130 -140 -150 -160 -170 -180 -190 -200 -210 -220 -230 -240 -250
f1 (ppm)

$^{1}$H and $^{13}$C spectra of 6-chloro-3-(4-methylpyridin-2-yl)quinazoline-2,4(1*H*,3*H*)-dione **3u**

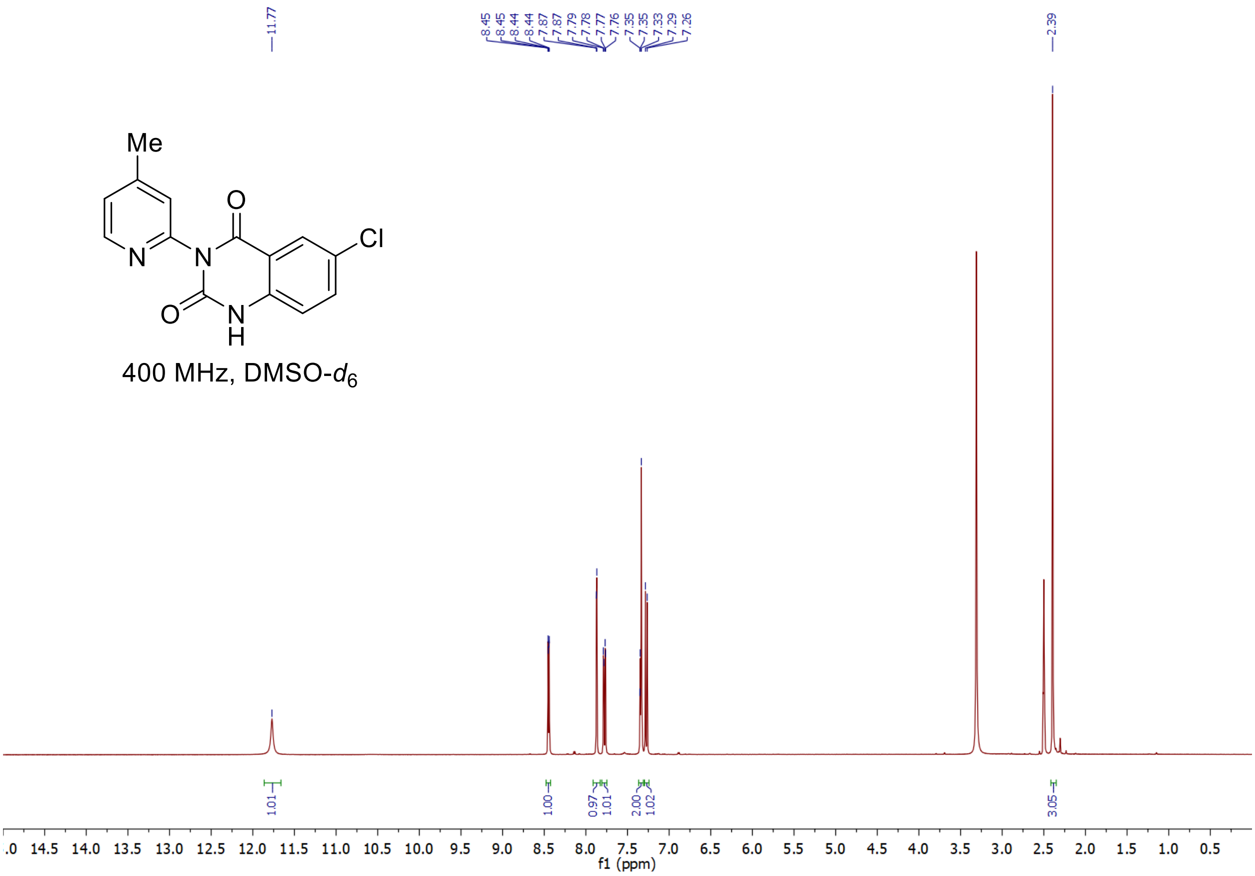


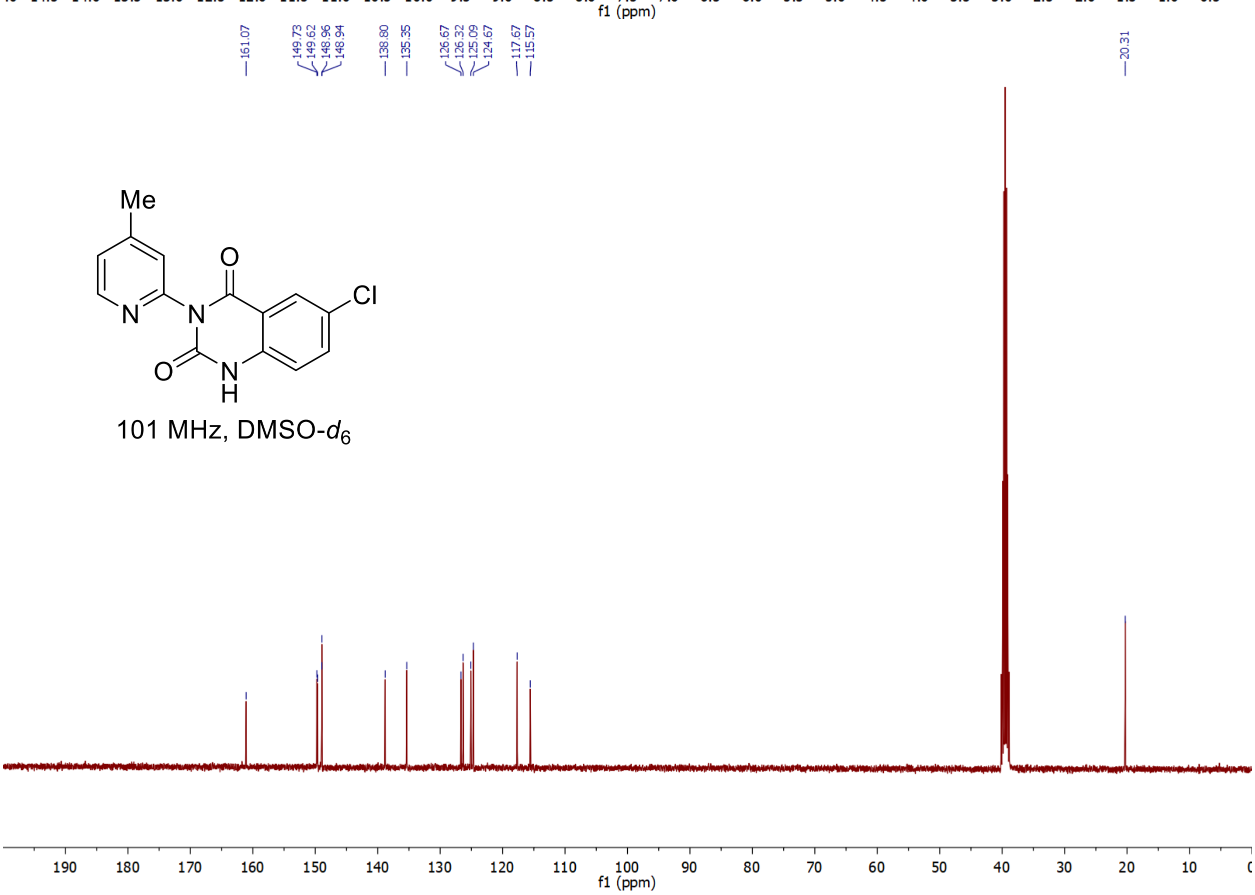

$^{1}H$ and $^{13}C$ spectra of 7-chloro-3-(4-methylpyridin-2-yl)quinazoline-2,4(1*H*,3*H*)-dione **3v**

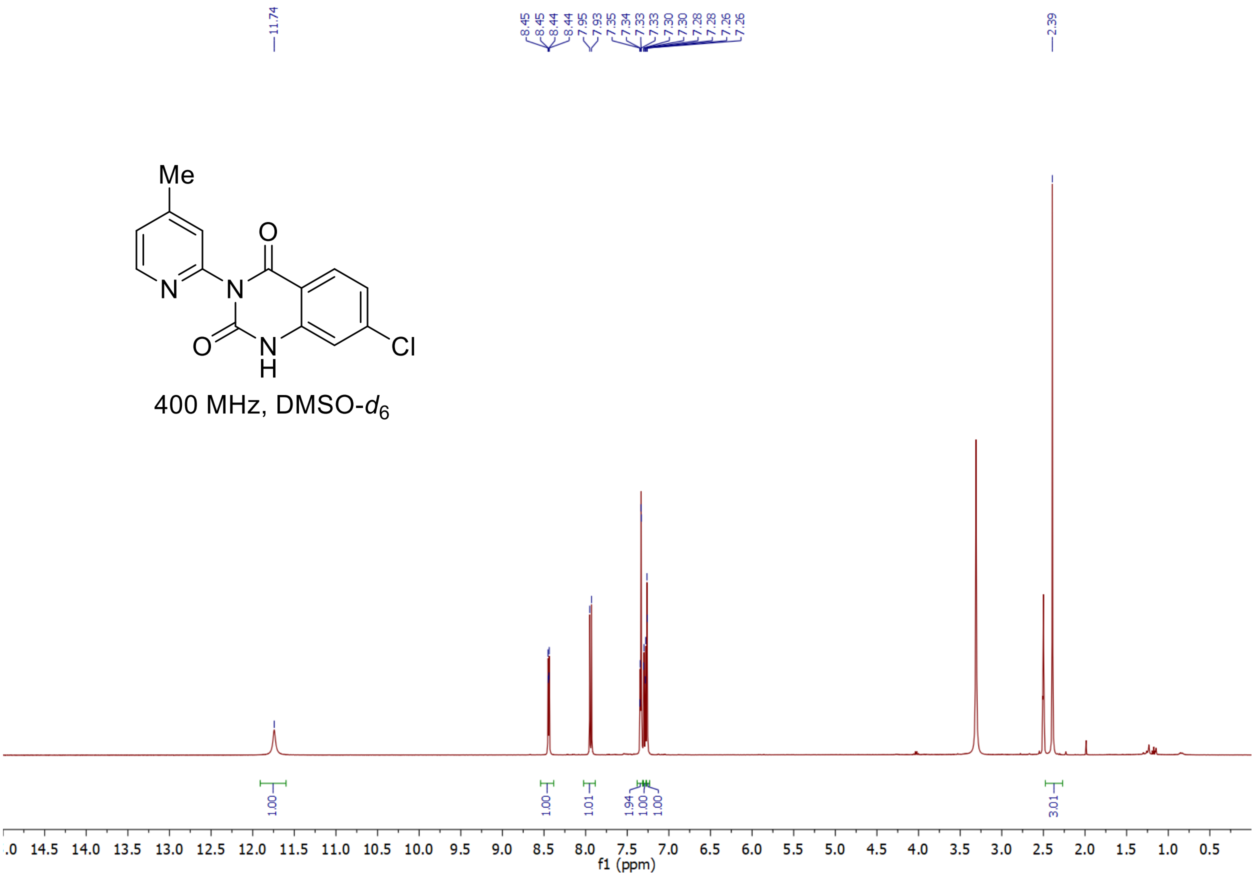


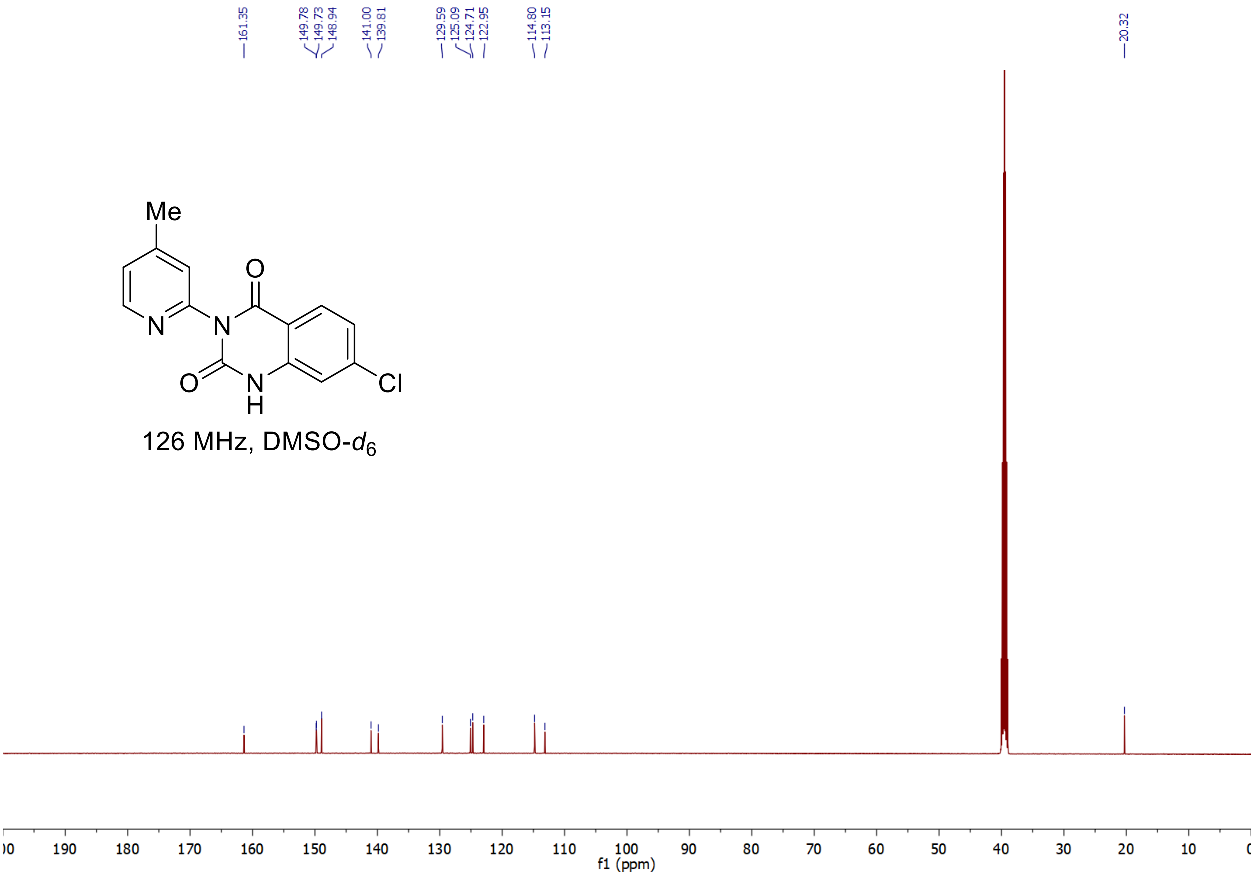

$^{1}H$ and $^{13}C$ spectra of 6-bromo-3-(4-methylpyridin-2-yl)quinazoline-2,4(1*H*,3*H*)-dione **3w**

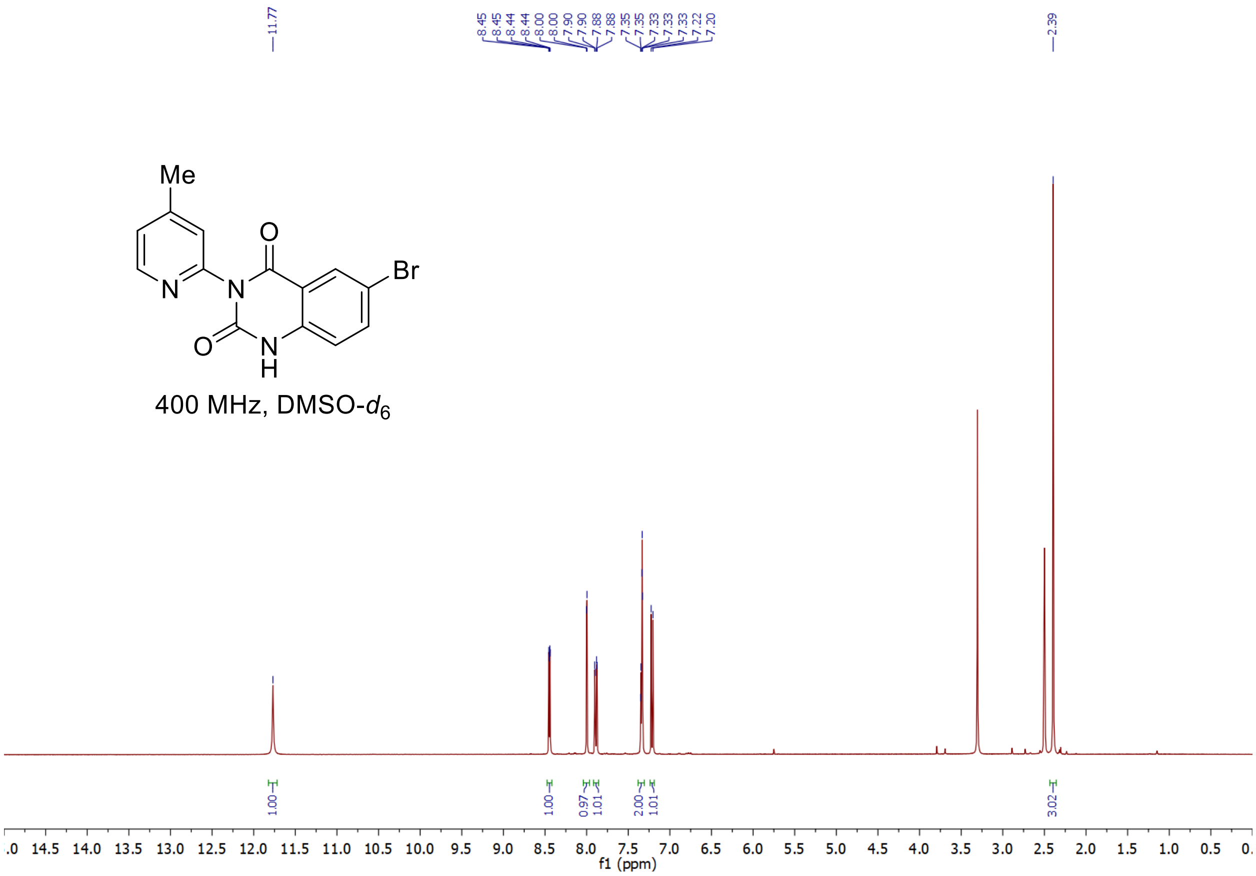


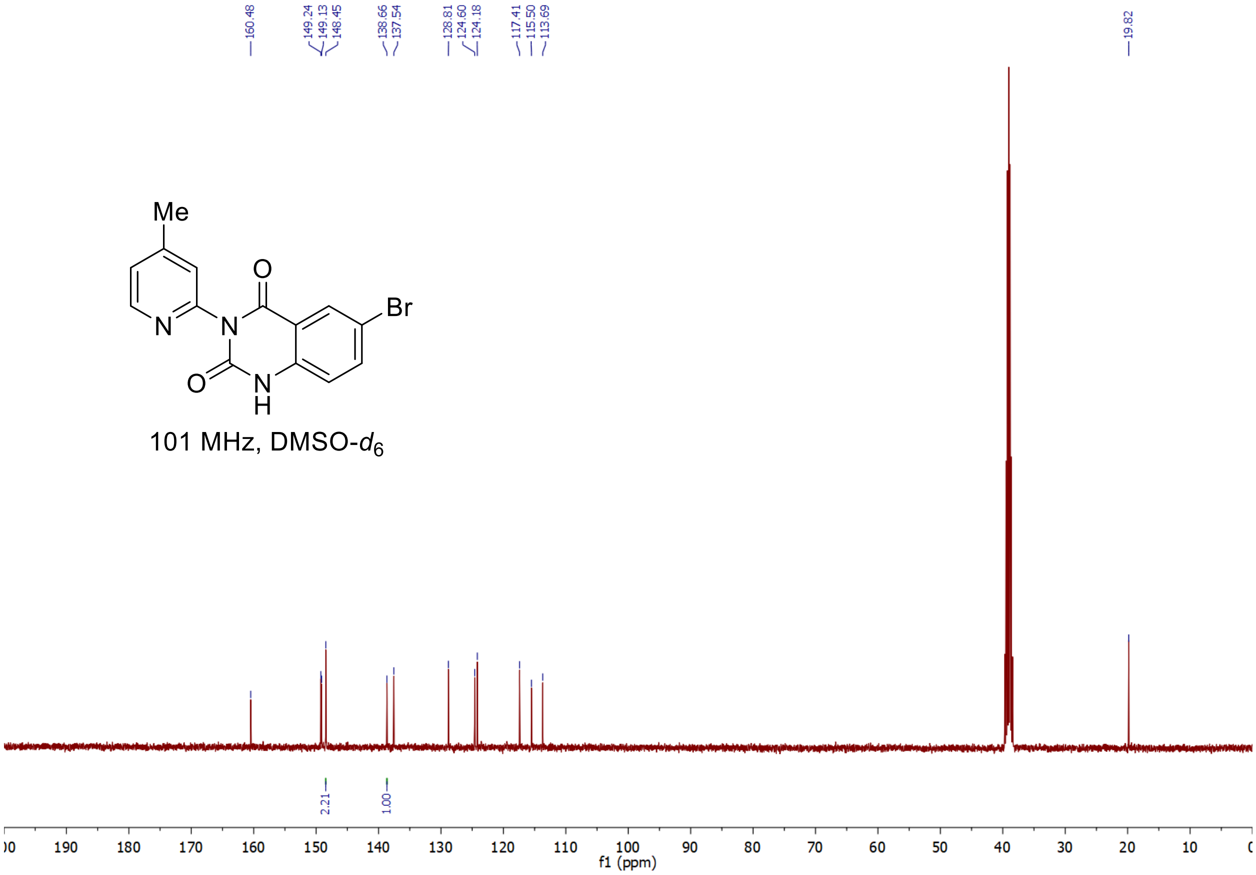

$^{1}$H and $^{13}$C spectra of methyl 3-(4-methylpyridin-2-yl)-2,4-dioxo-1,2,3,4-tetrahydroquinazoline-7-carboxylate **3x**

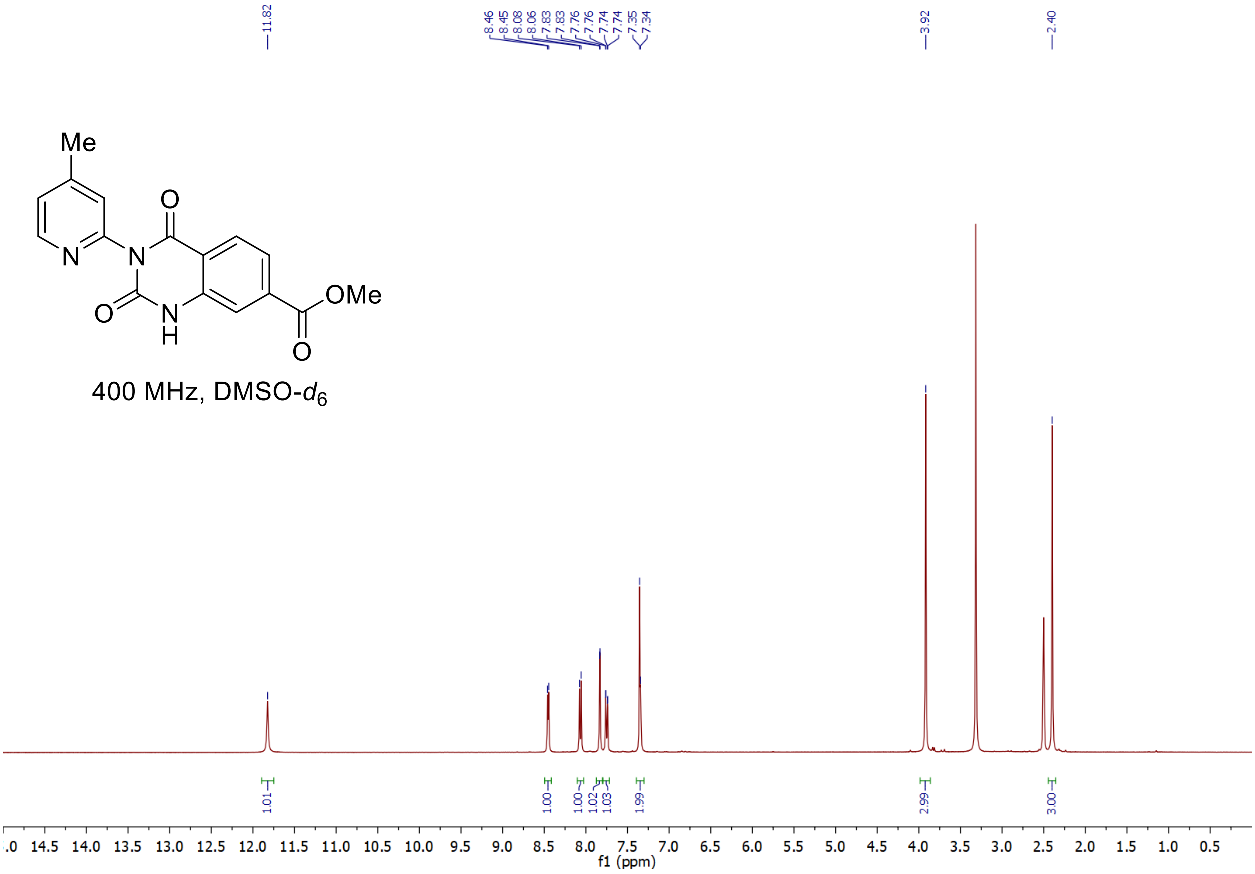


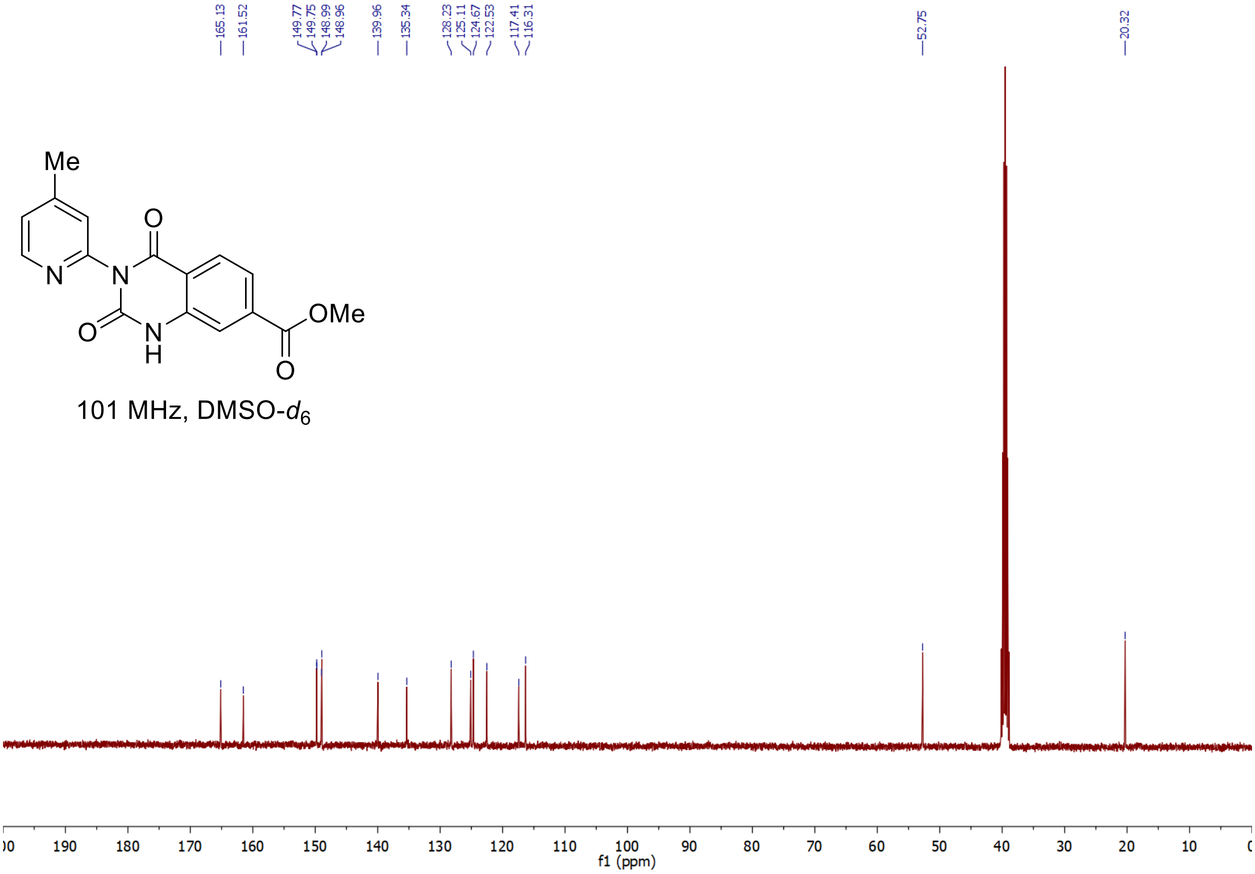

$^{1}$H and $^{13}$C spectra of 1-methyl-3-(4-methylpyridin-2-yl)quinazoline-2,4(1*H*,3*H*)-dione **3y**

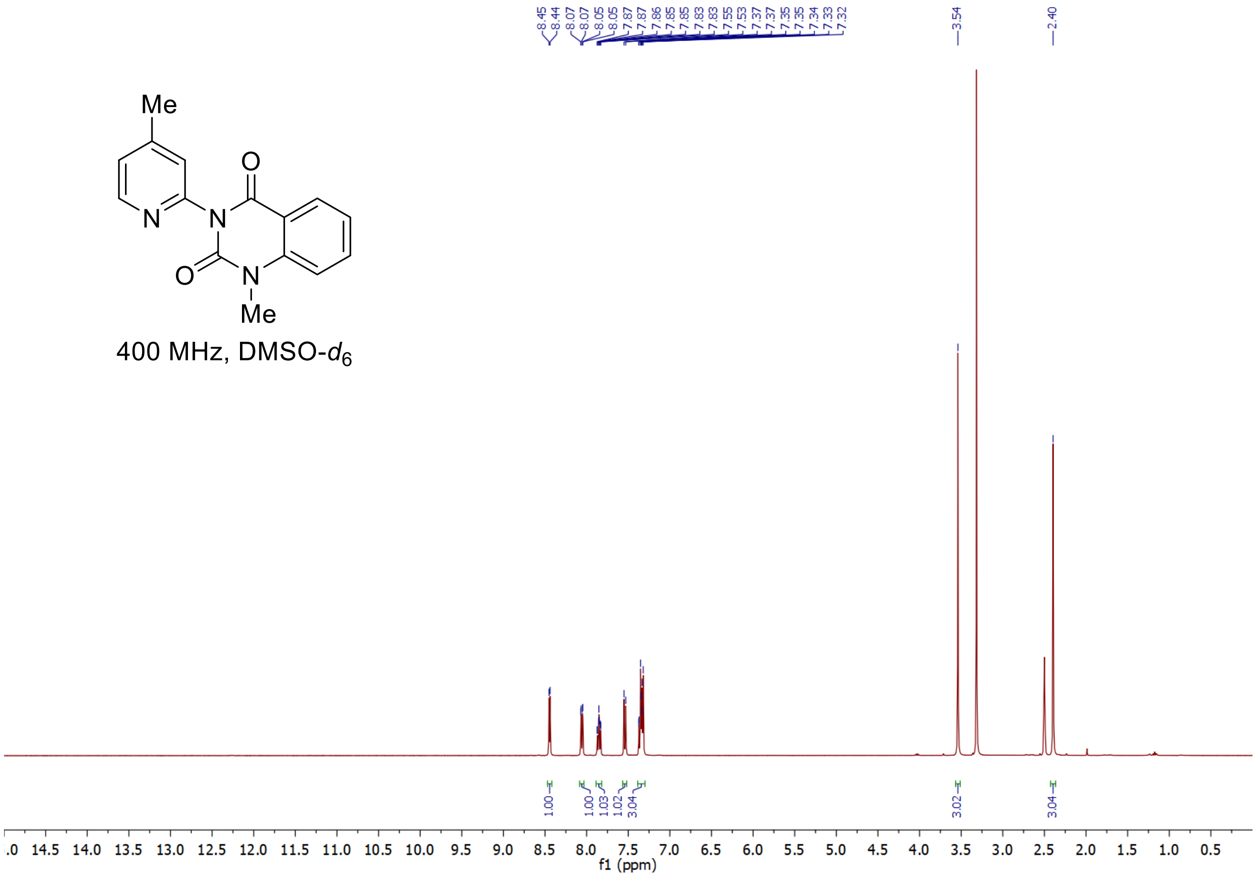


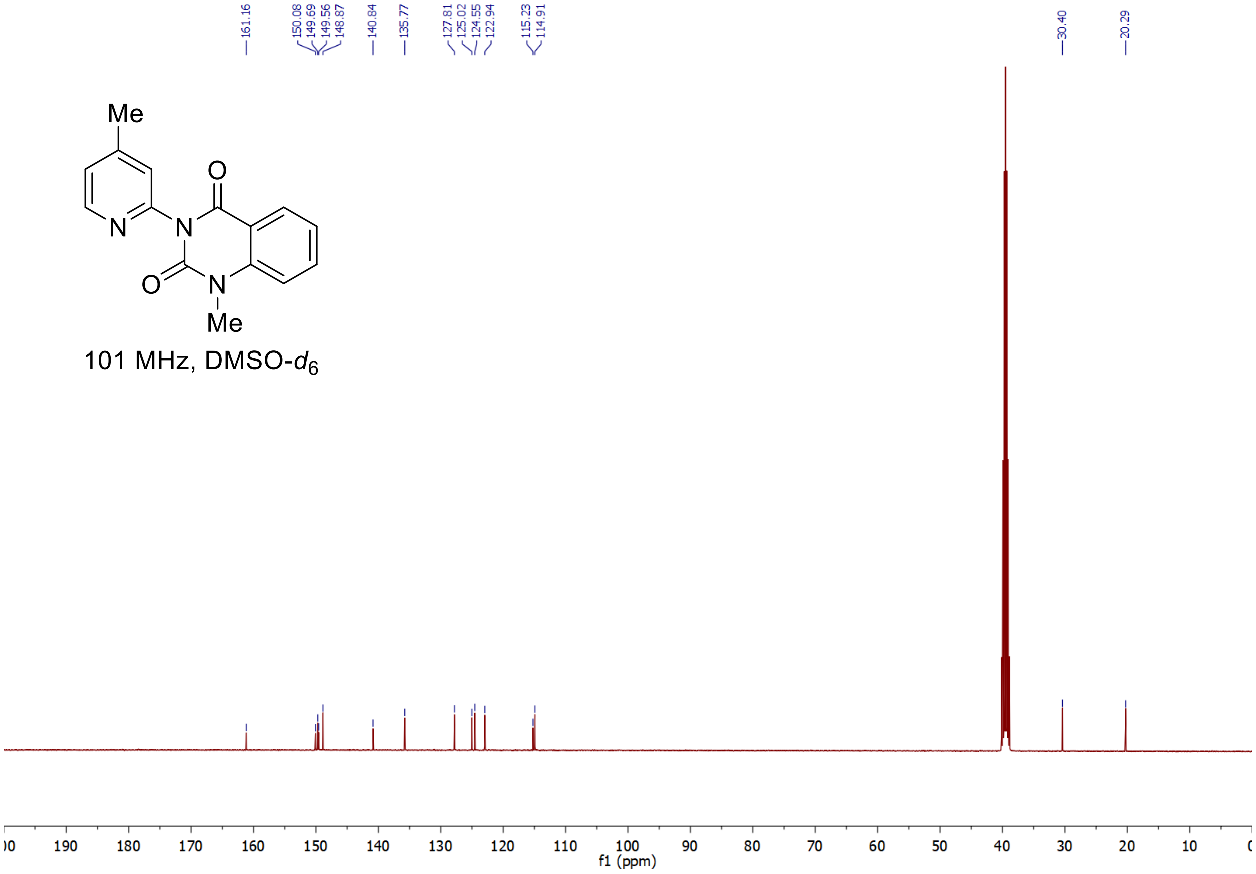

$^{1}$H and $^{13}$C spectra of 6,7-dimethoxy-3-(4-methoxypyridin-2-yl)quinazoline-2,4(1*H*,3*H*)-dione **3z**

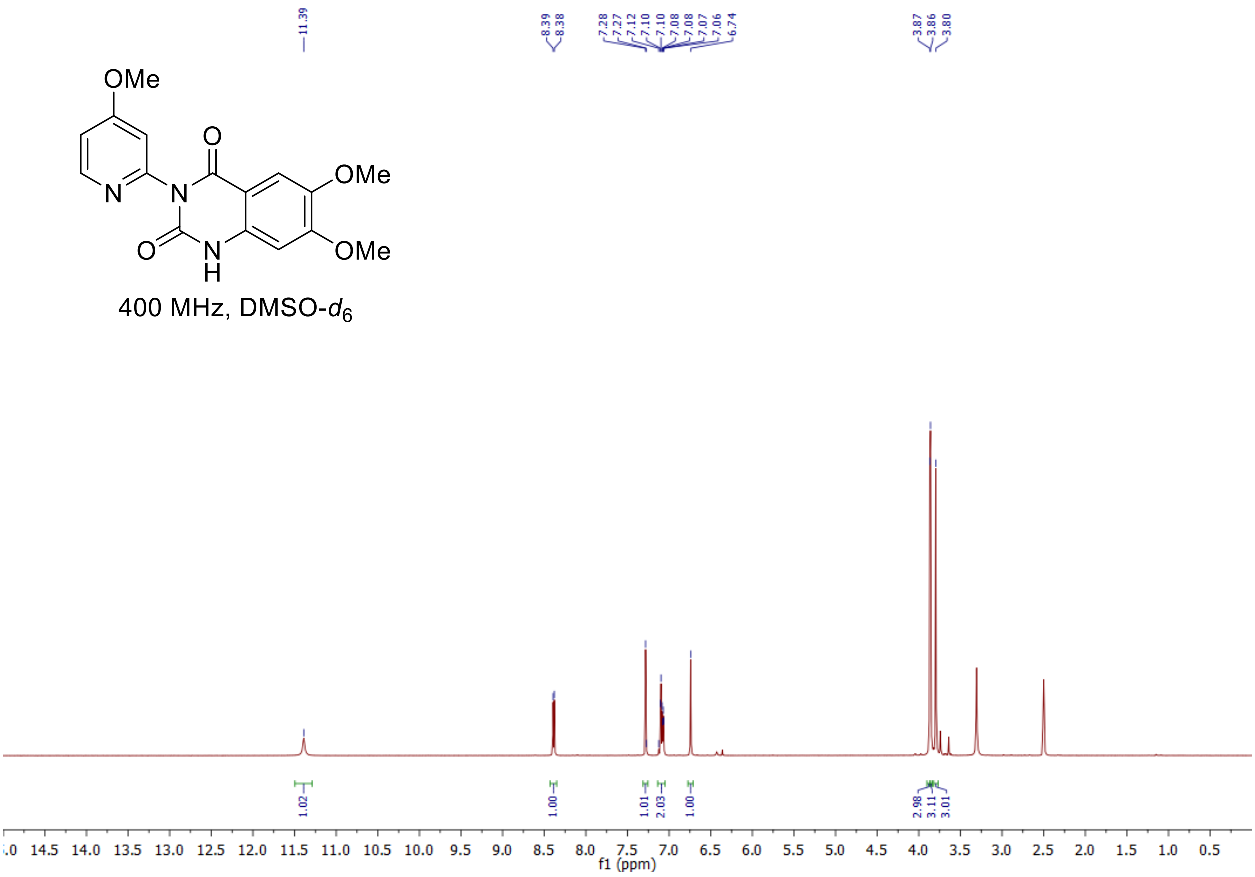


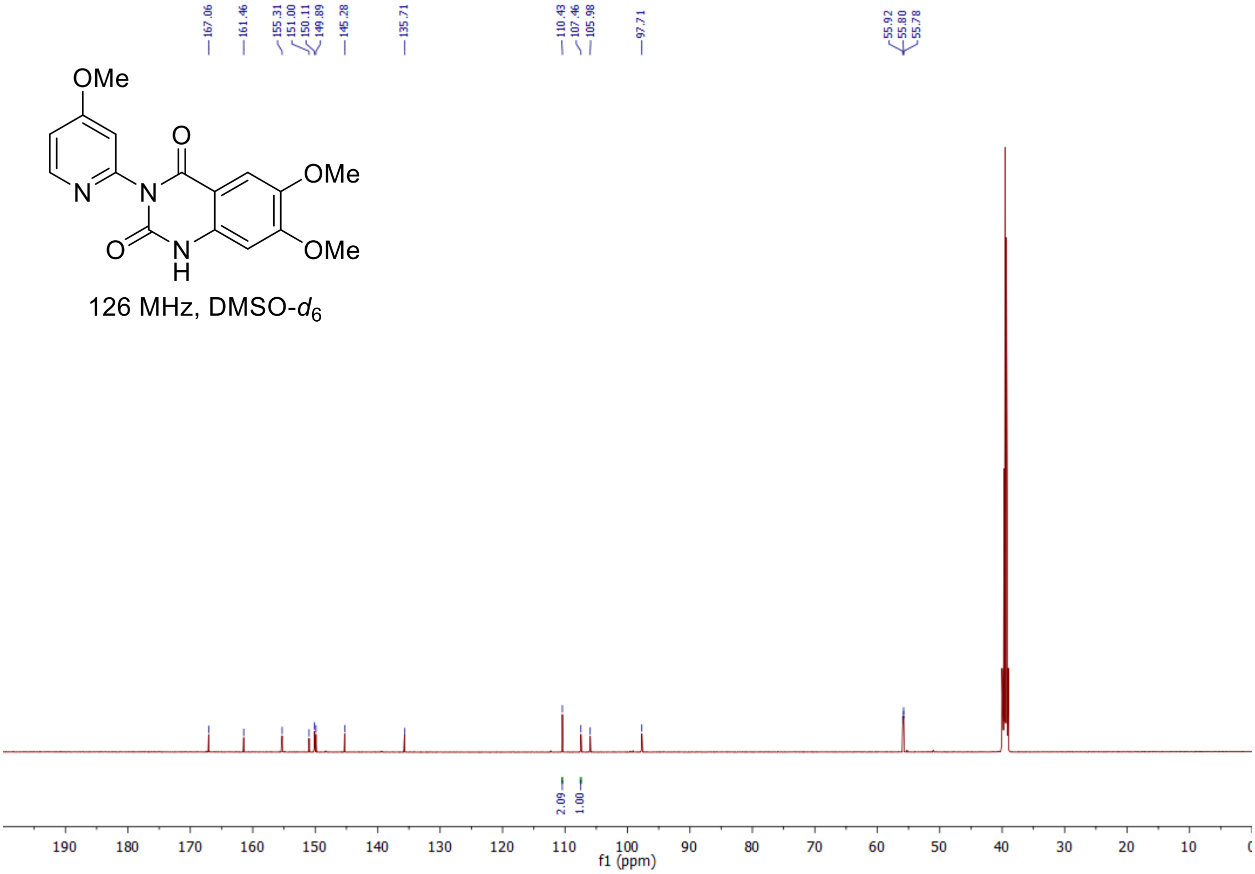

$^{1}H$ and $^{13}C$ spectra of ethyl 2-(3-(5-cyanopyridin-2-yl)ureido)benzoate **4b**

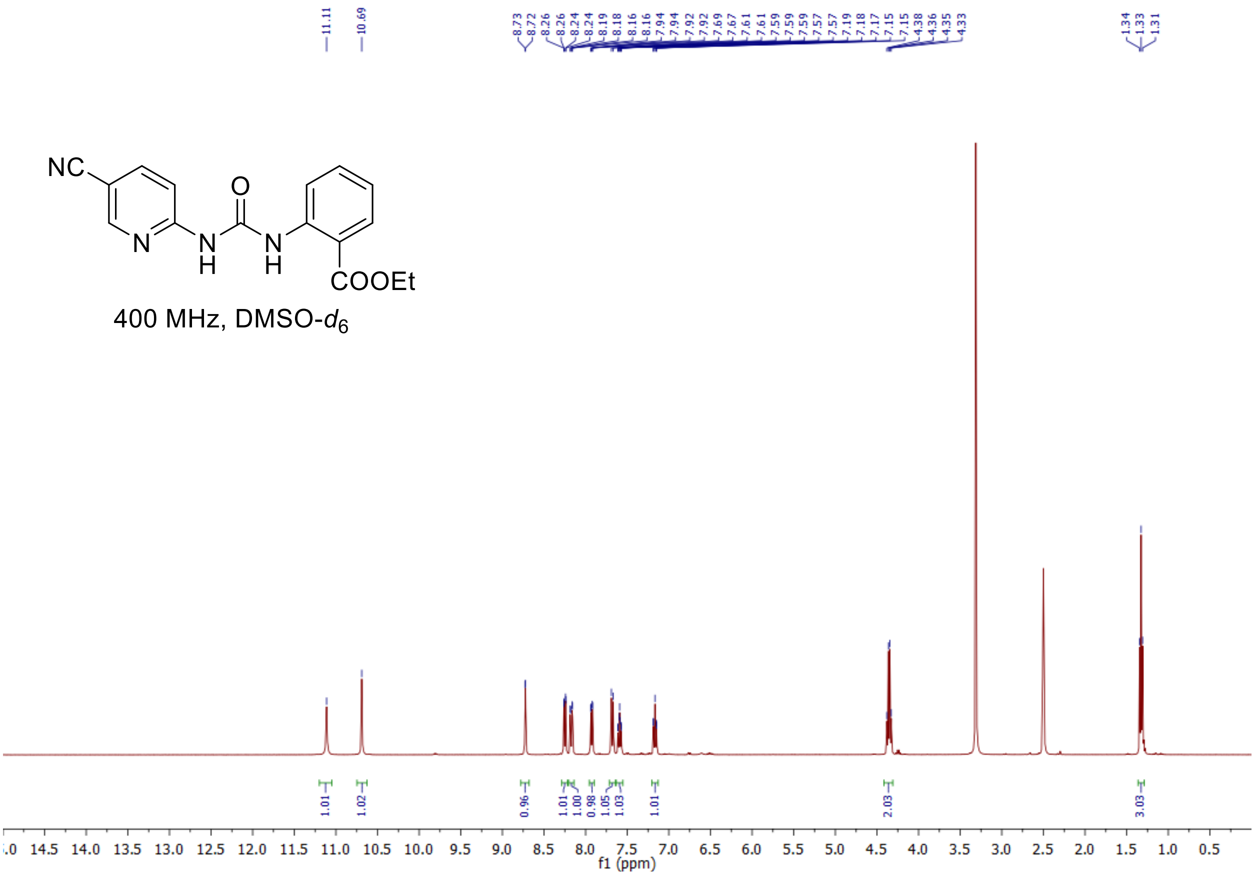


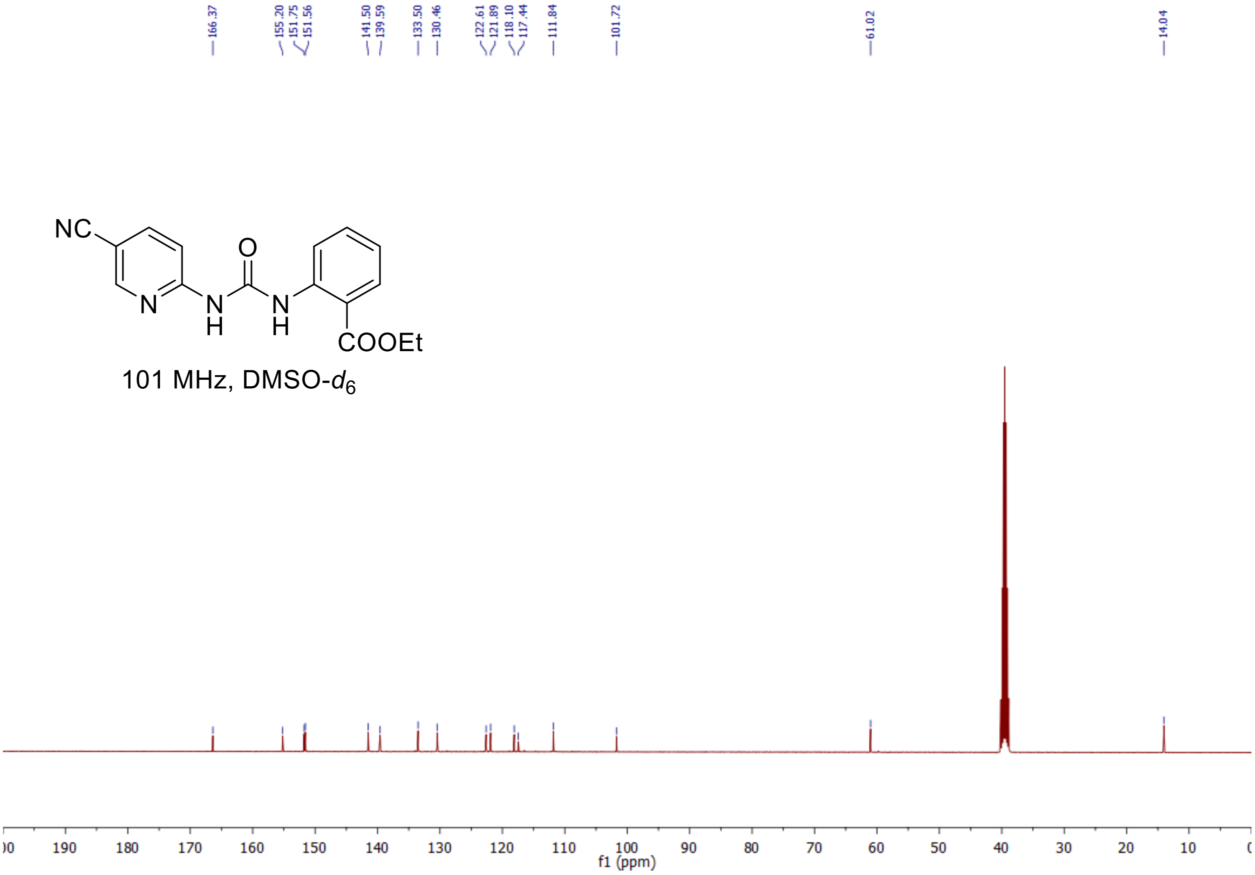

$^{1}$H and $^{13}$C spectra of ethyl 2-(3-(4-methylpyridin-2-yl)ureido)-4,5,6,7-tetrahydrobenzo[*b*]thiophene-3-carboxylate **7a**

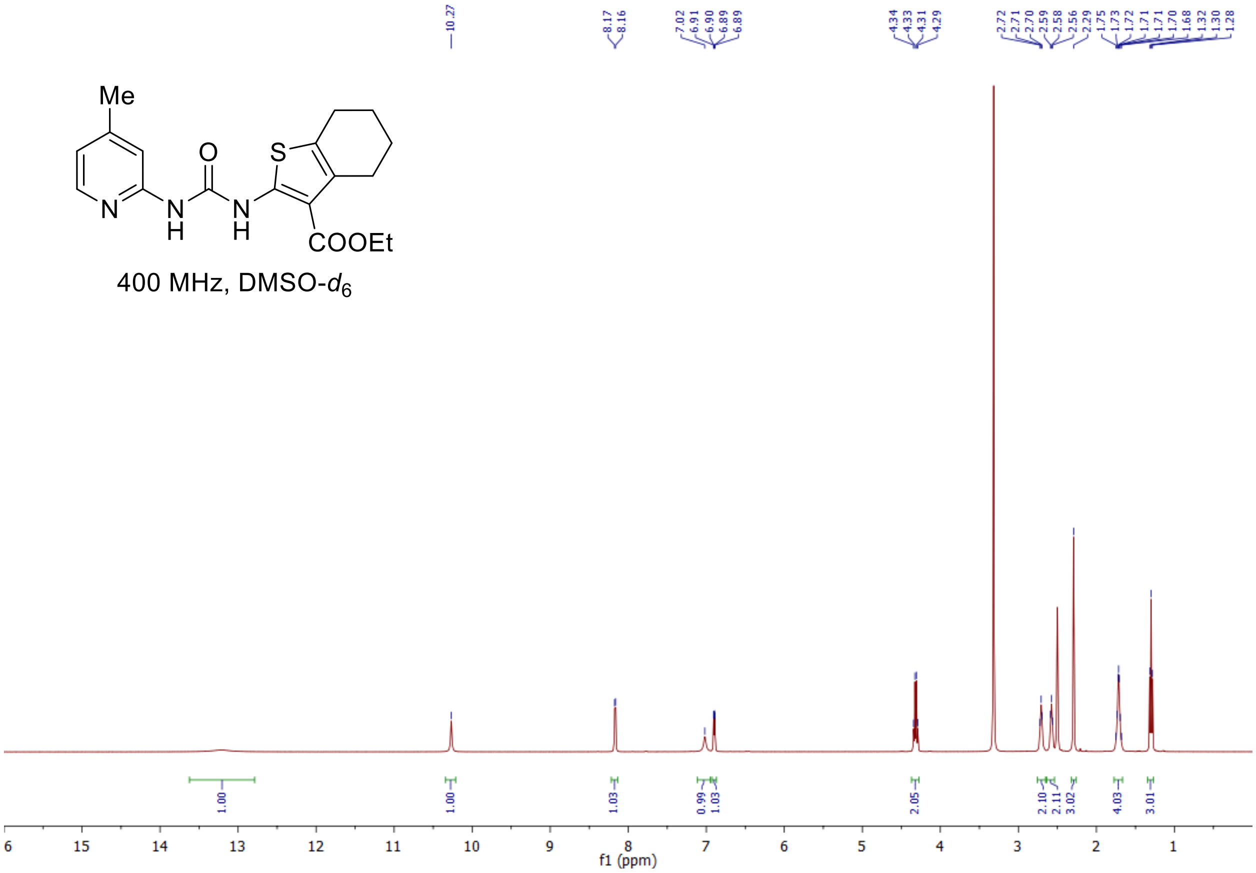


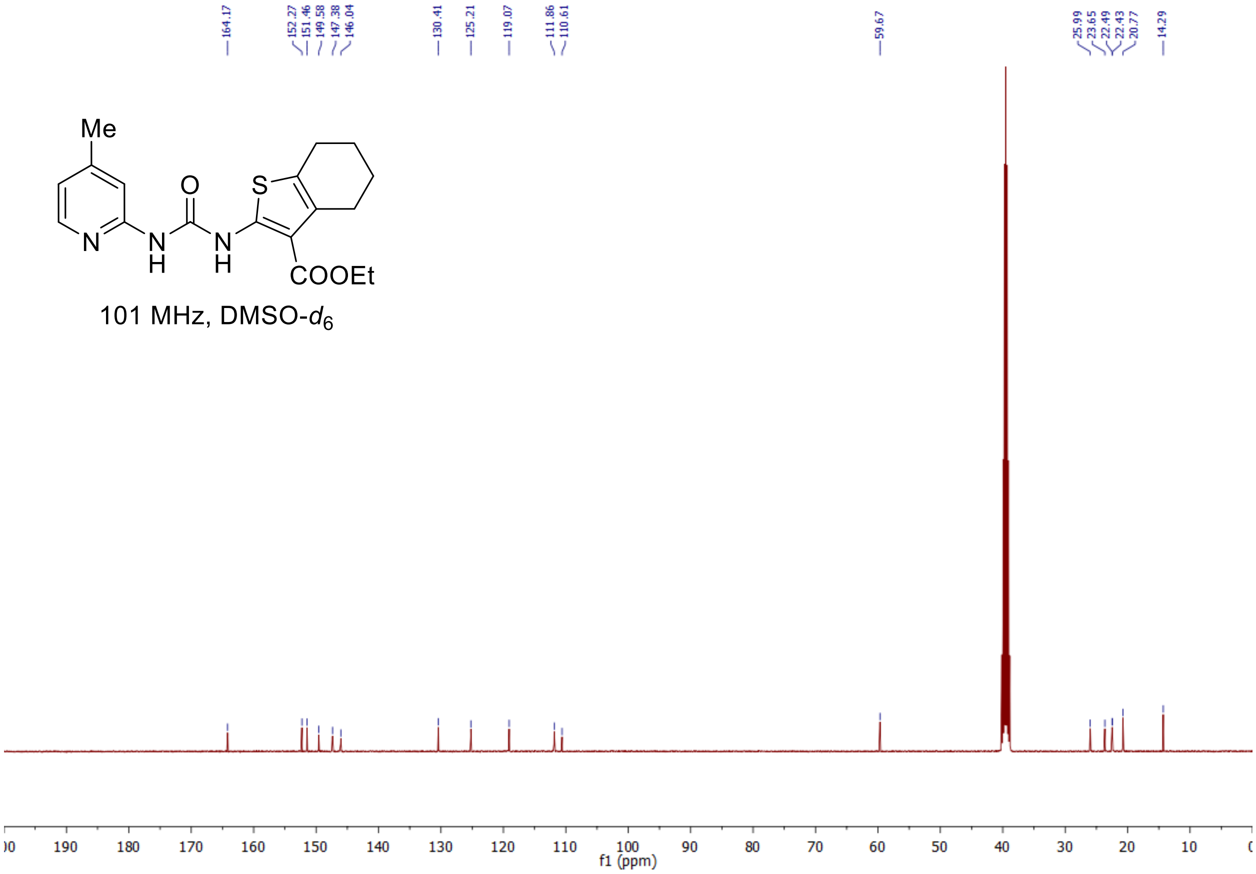

<sup></sup>

$^{1}$H and $^{13}$C spectra of 3-(4-methylpyridin-2-yl)-5,6,7,8-tetrahydrobenzo[4,5]thieno[2,3-*d*]pyrimidine-2,4(1*H*,3*H*)-dione **8a**

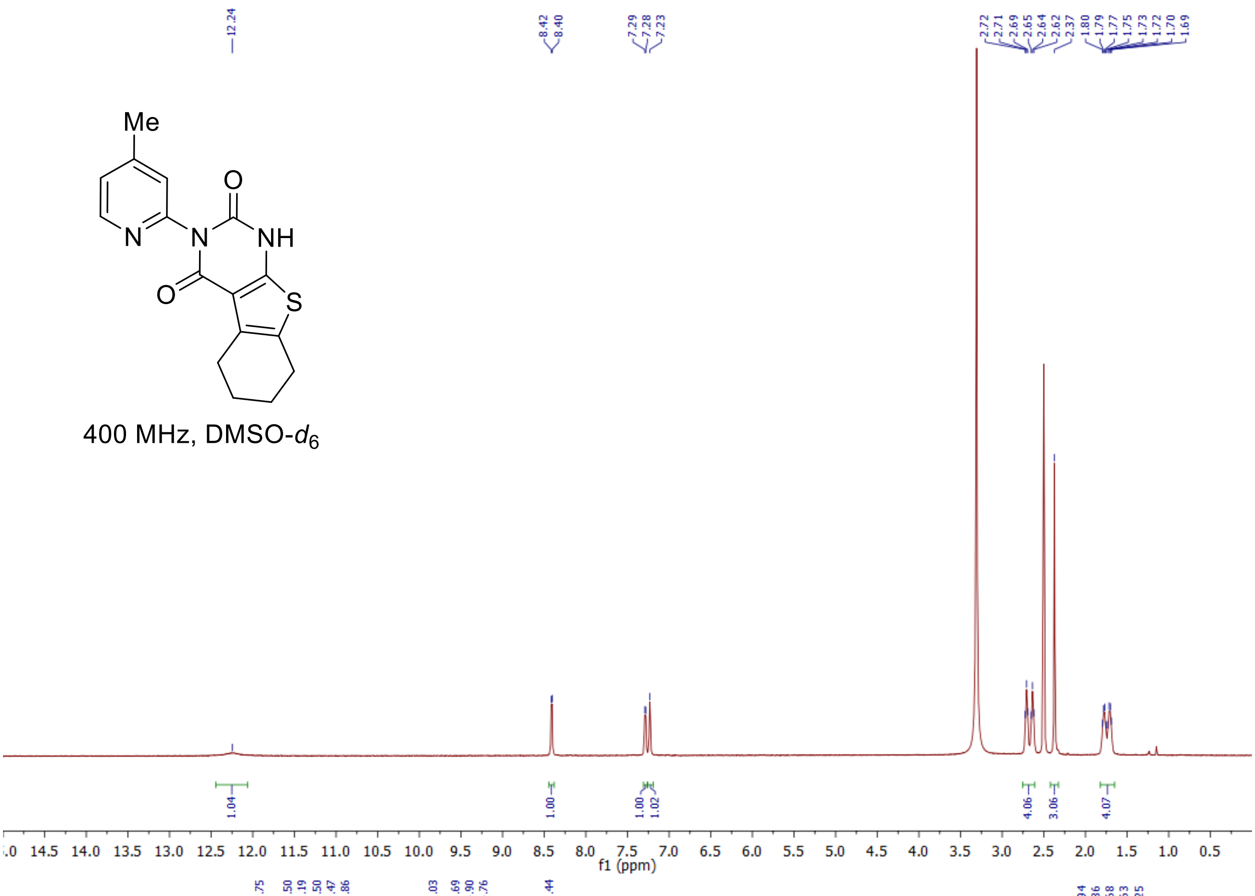


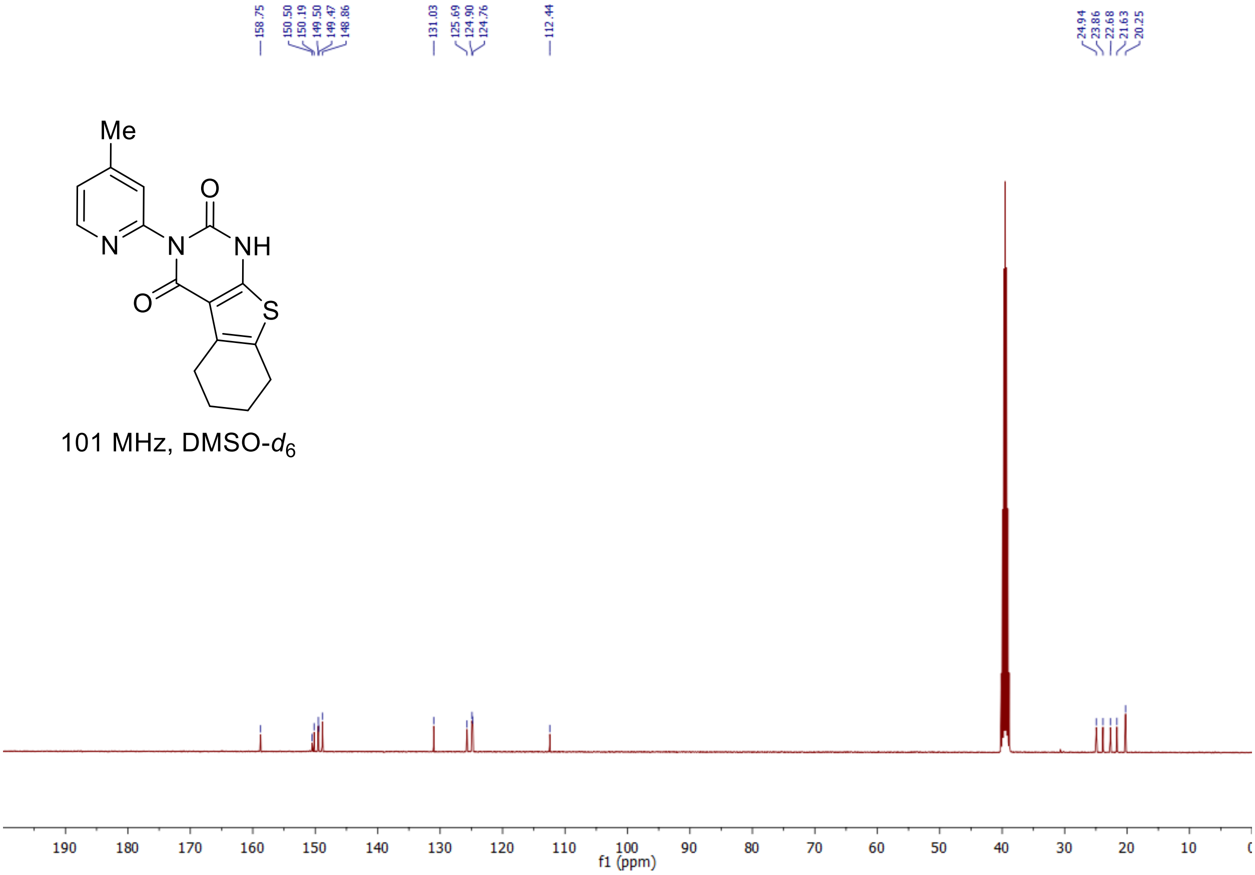

$^{1}$H and $^{13}$C spectra of 3-(pyridin-2-yl)-5,6,7,8-tetrahydrobenzo[4,5]thieno[2,3-*d*]pyrimidine-2,4(1*H*,3*H*)-dione **8b**

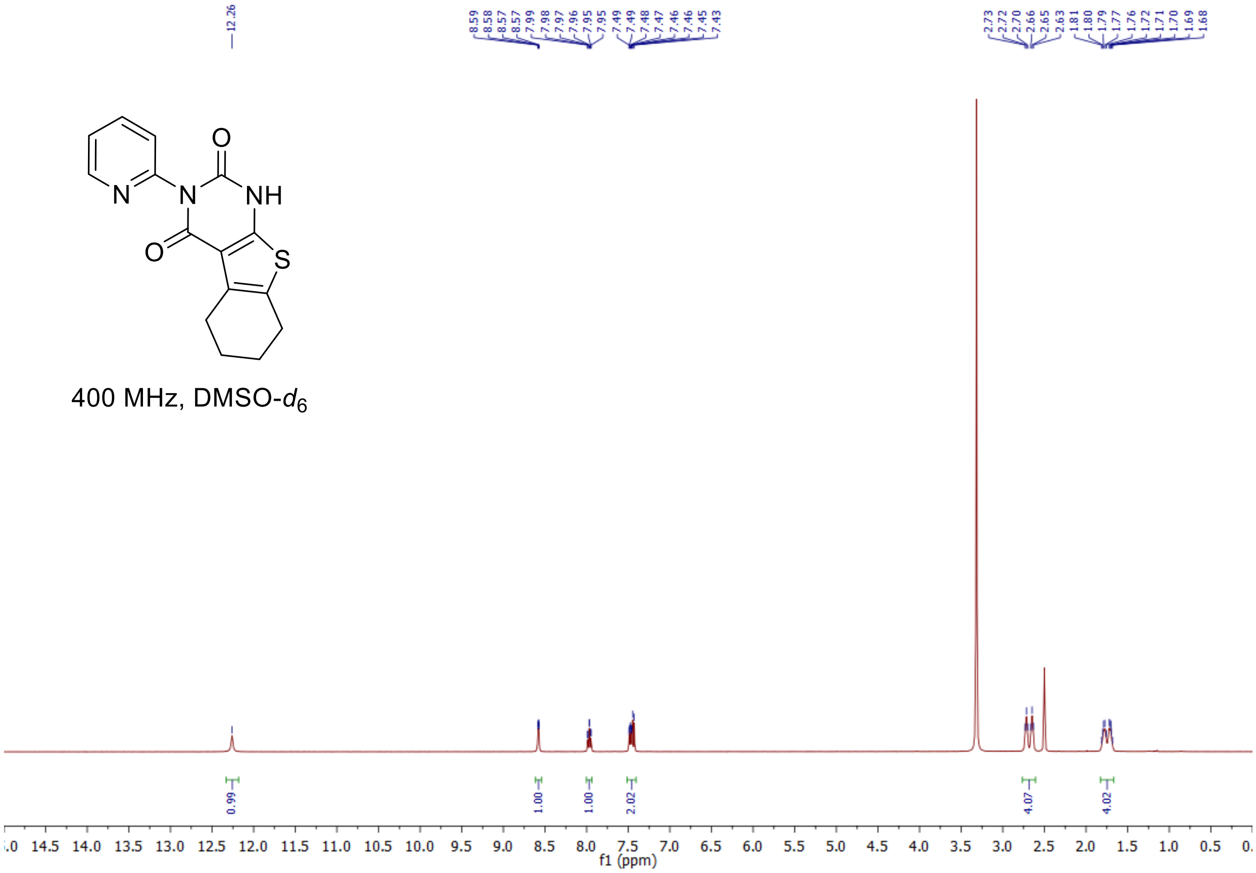


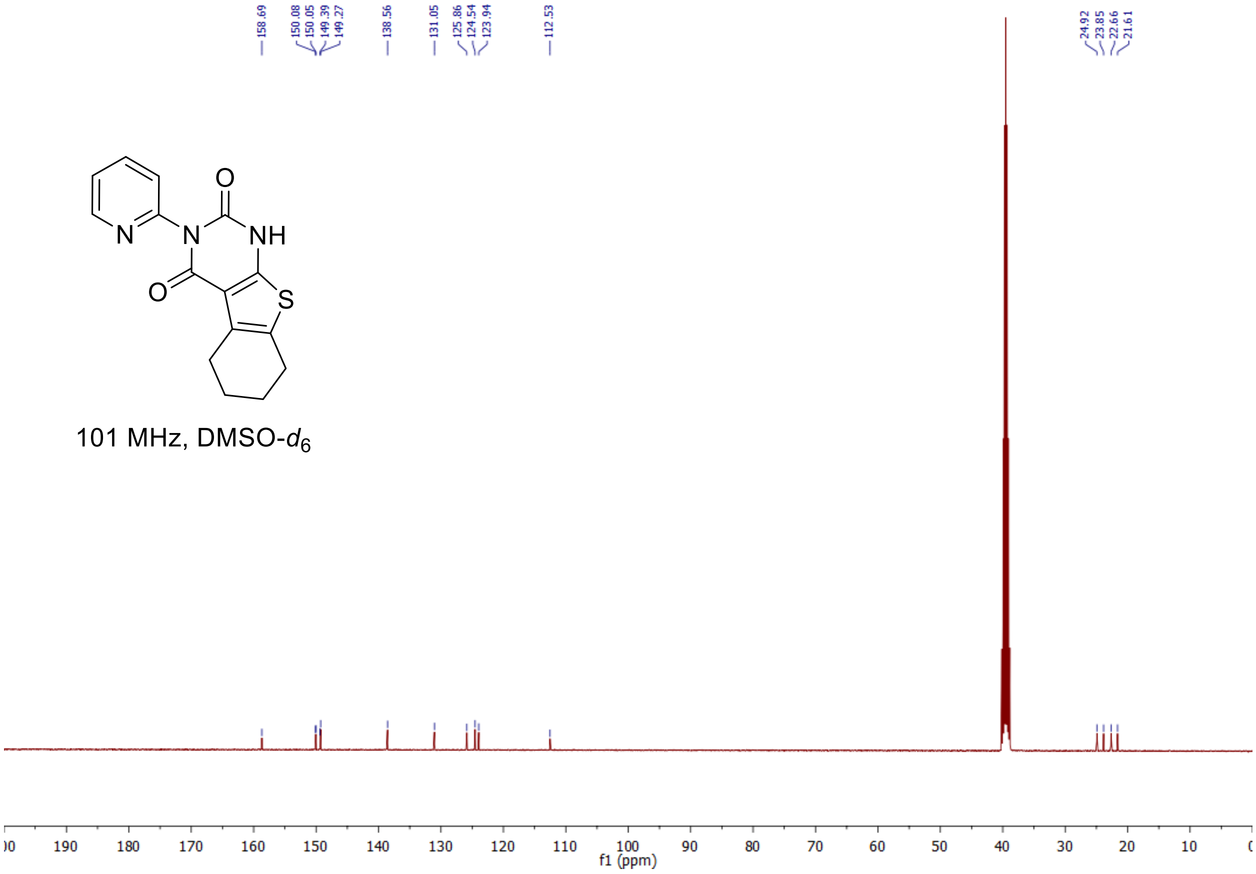

$^{1}H$ and $^{13}C$ spectra of 3-(4-methoxypyridin-2-yl)-5,6,7,8-tetrahydrobenzo[4,5]thieno[2,3-*d*]pyrimidine-2,4(1*H*,3*H*)-dione **8c**

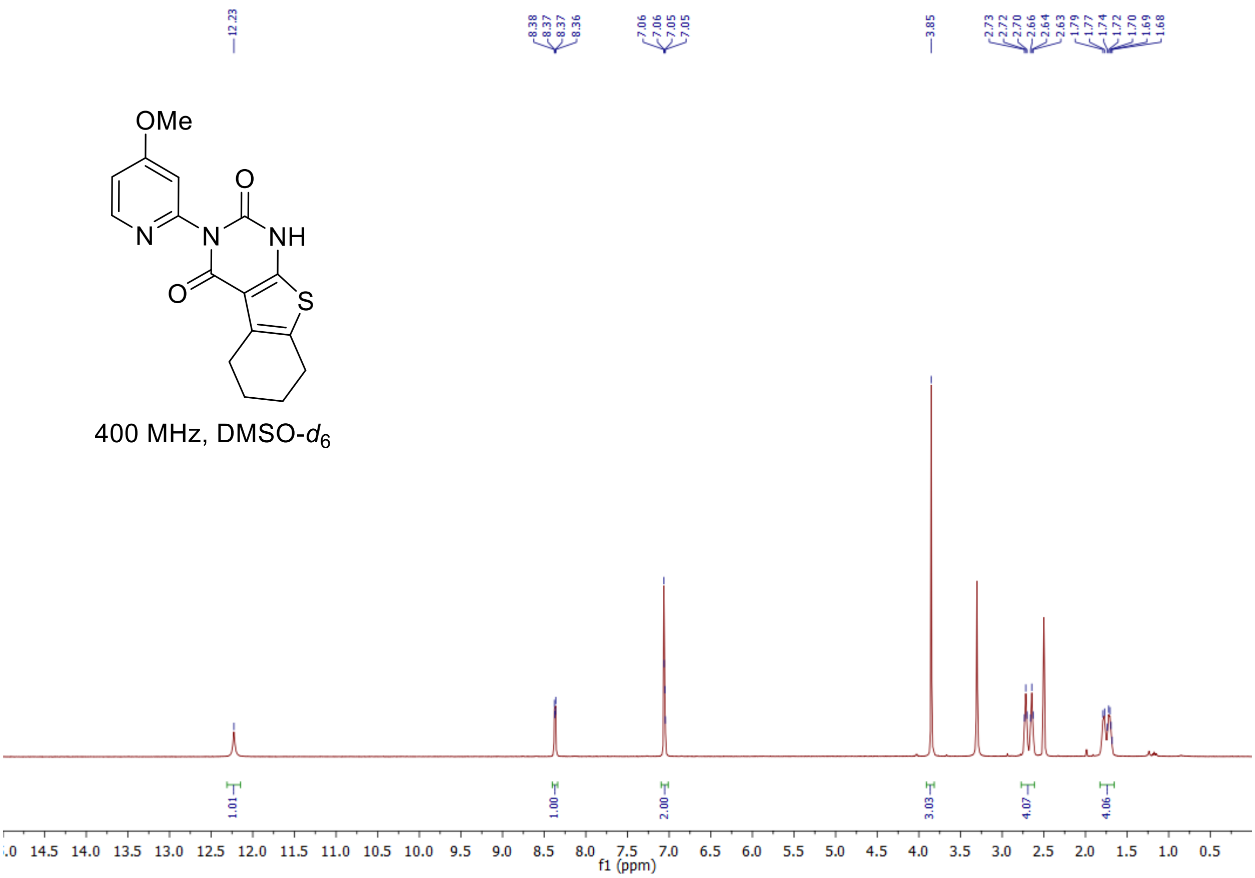


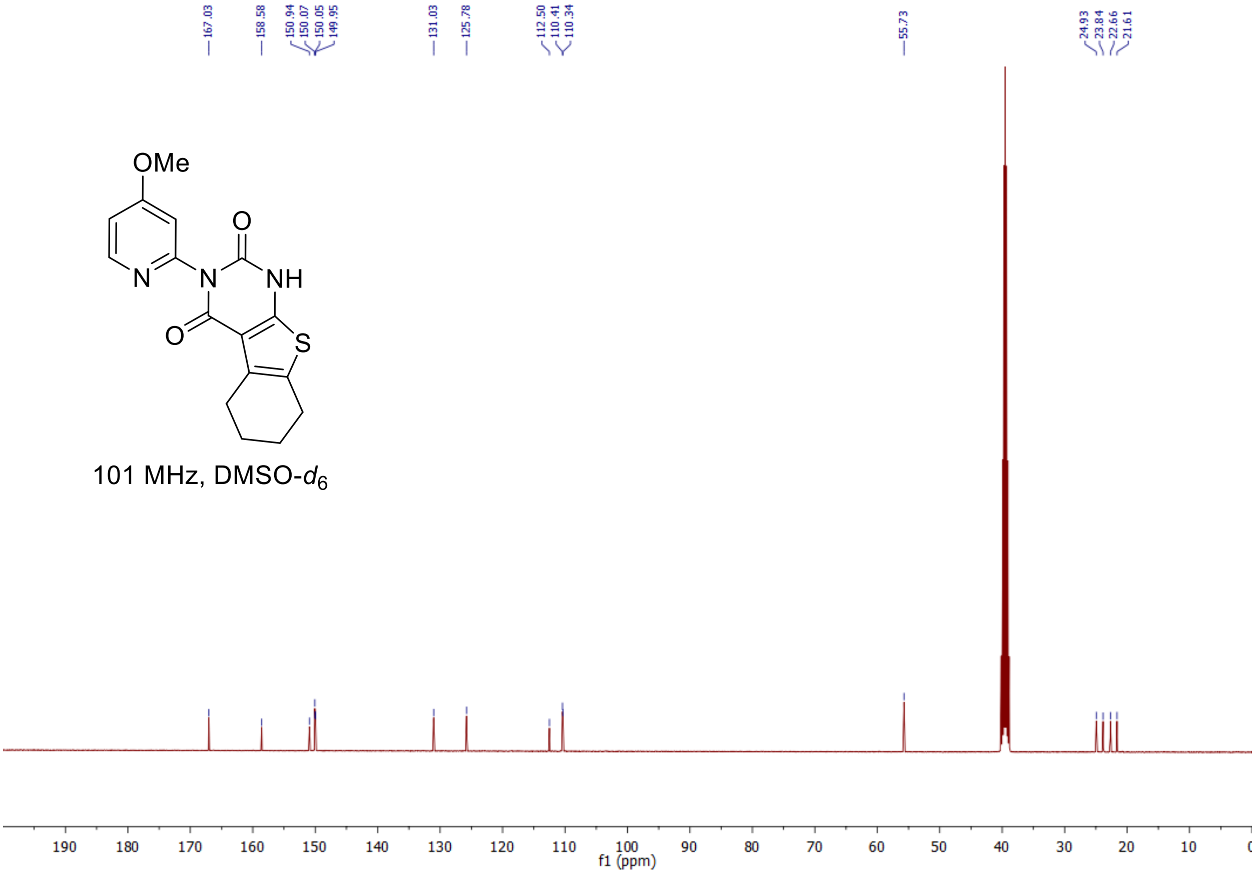

$^{1}H$ and $^{13}C$ spectra of 3-(6-phenylpyridin-2-yl)-5,6,7,8-tetrahydrobenzo[4,5]thieno[2,3-*d*]pyrimidine-2,4(1*H*,3*H*)-dione **8d**

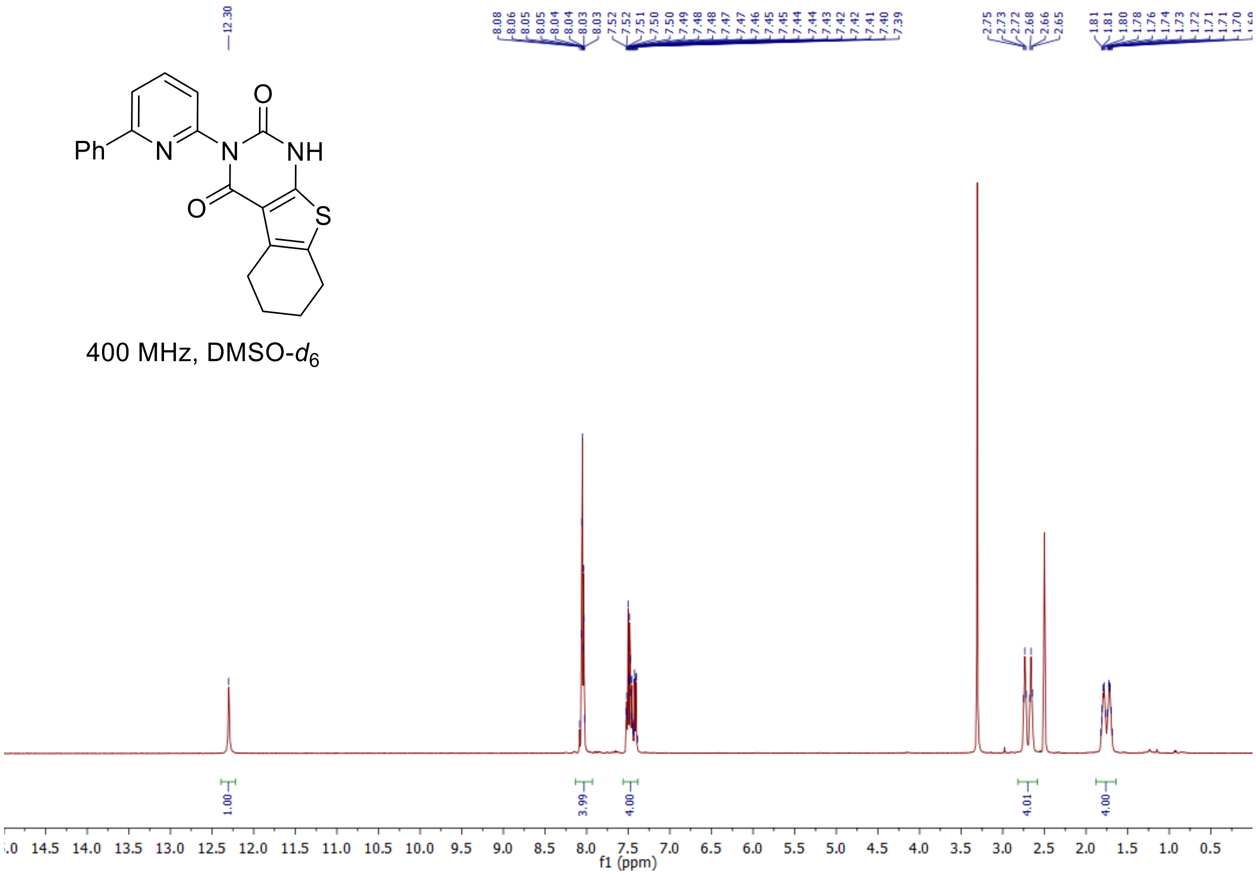


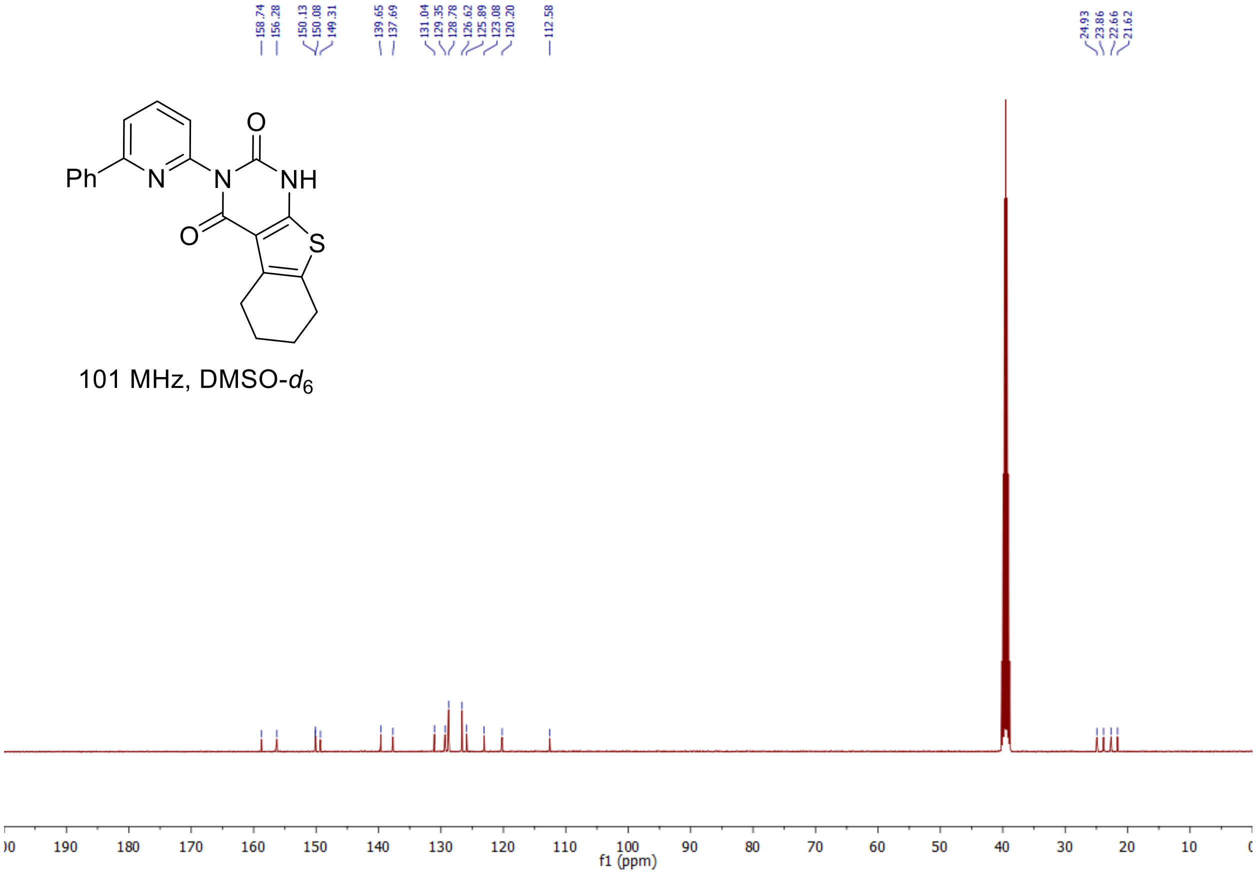

$^{1}$H and $^{13}$C spectra of 3-(quinolin-2-yl)-5,6,7,8-tetrahydrobenzo[4,5]thieno[2,3-*d*]pyrimidine-2,4(1*H*,3*H*)-dione **8e**

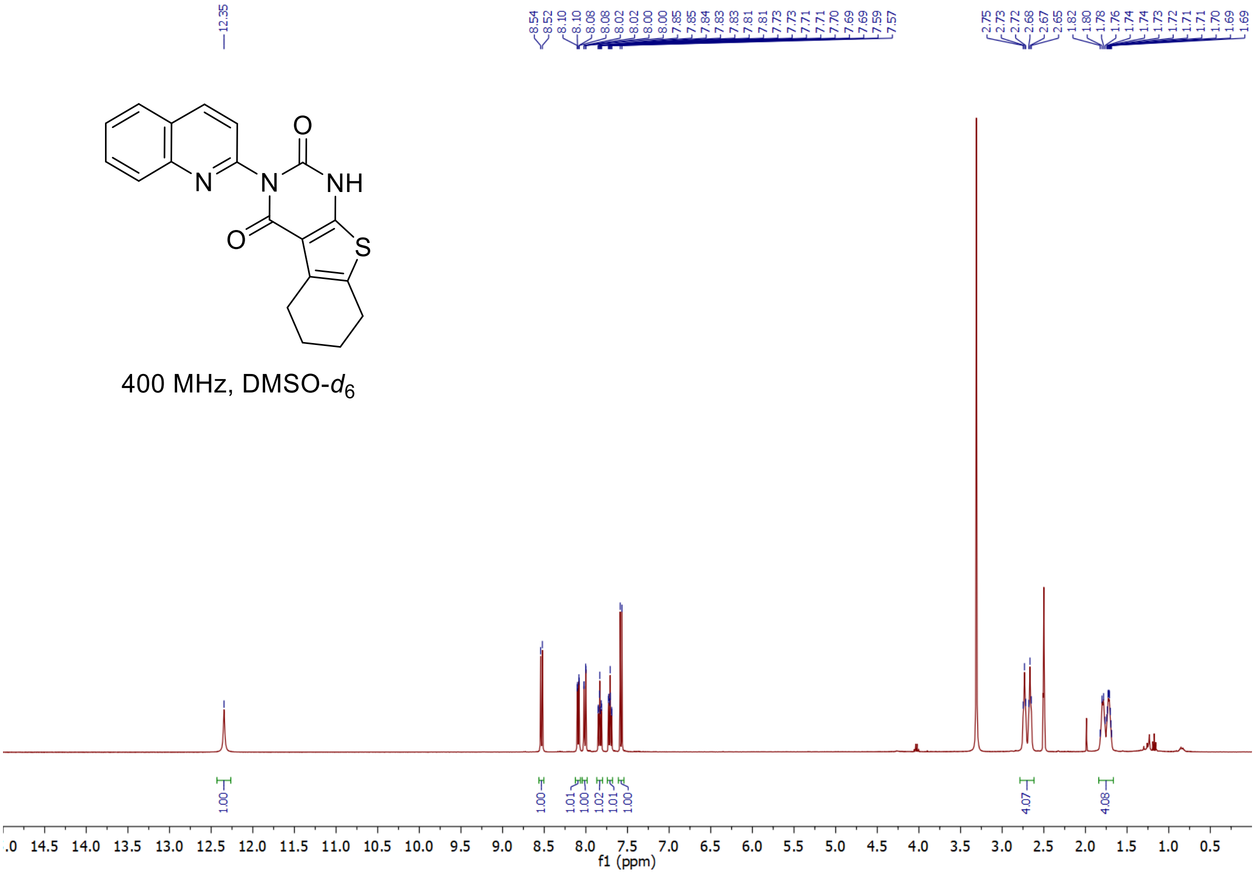


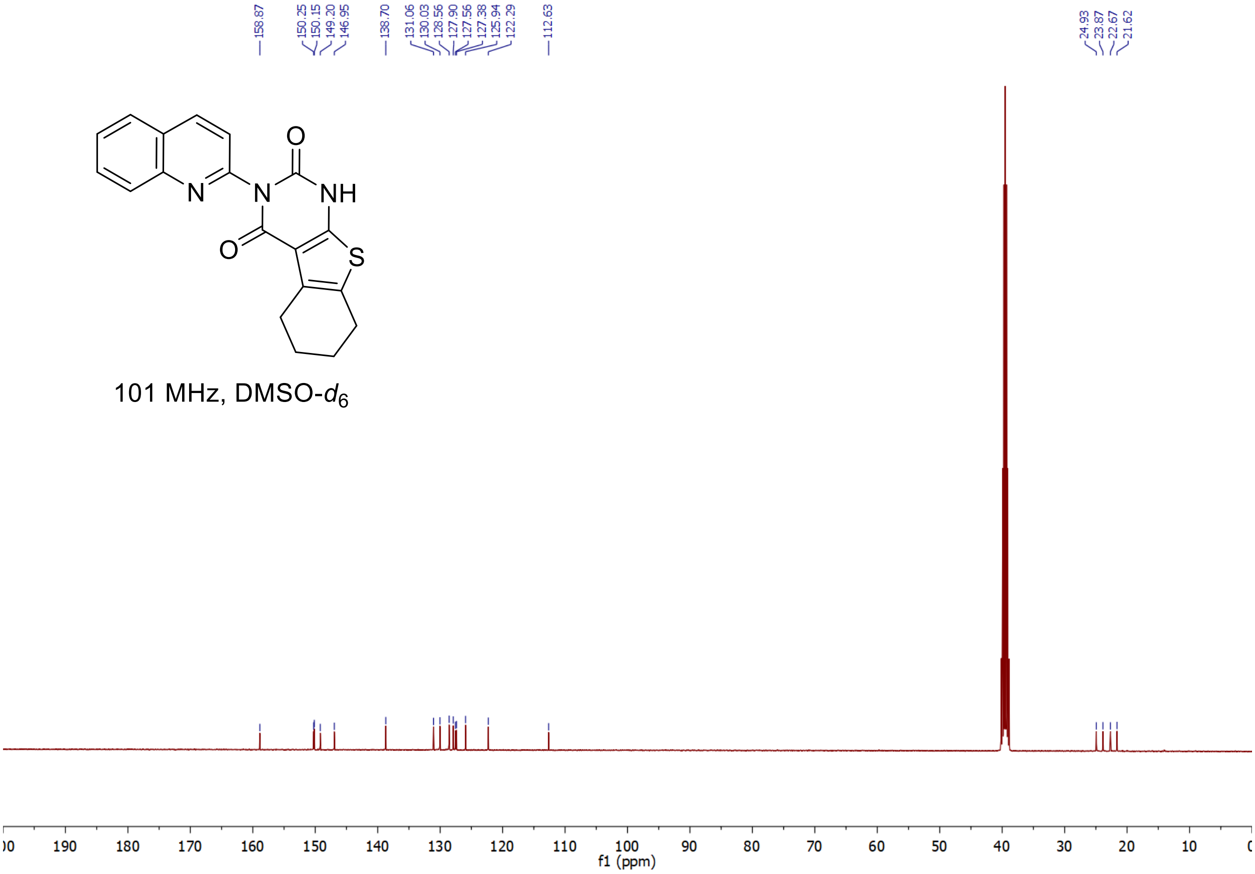

$^{1}H$ and $^{13}C$ spectra of 3-(4-methylpyridin-2-yl)-1,5,6,7-tetrahydro-2*H*-cyclopenta[4,5]thieno[2,3-*d*]pyrimidine-2,4(3*H*)-dione **8f**

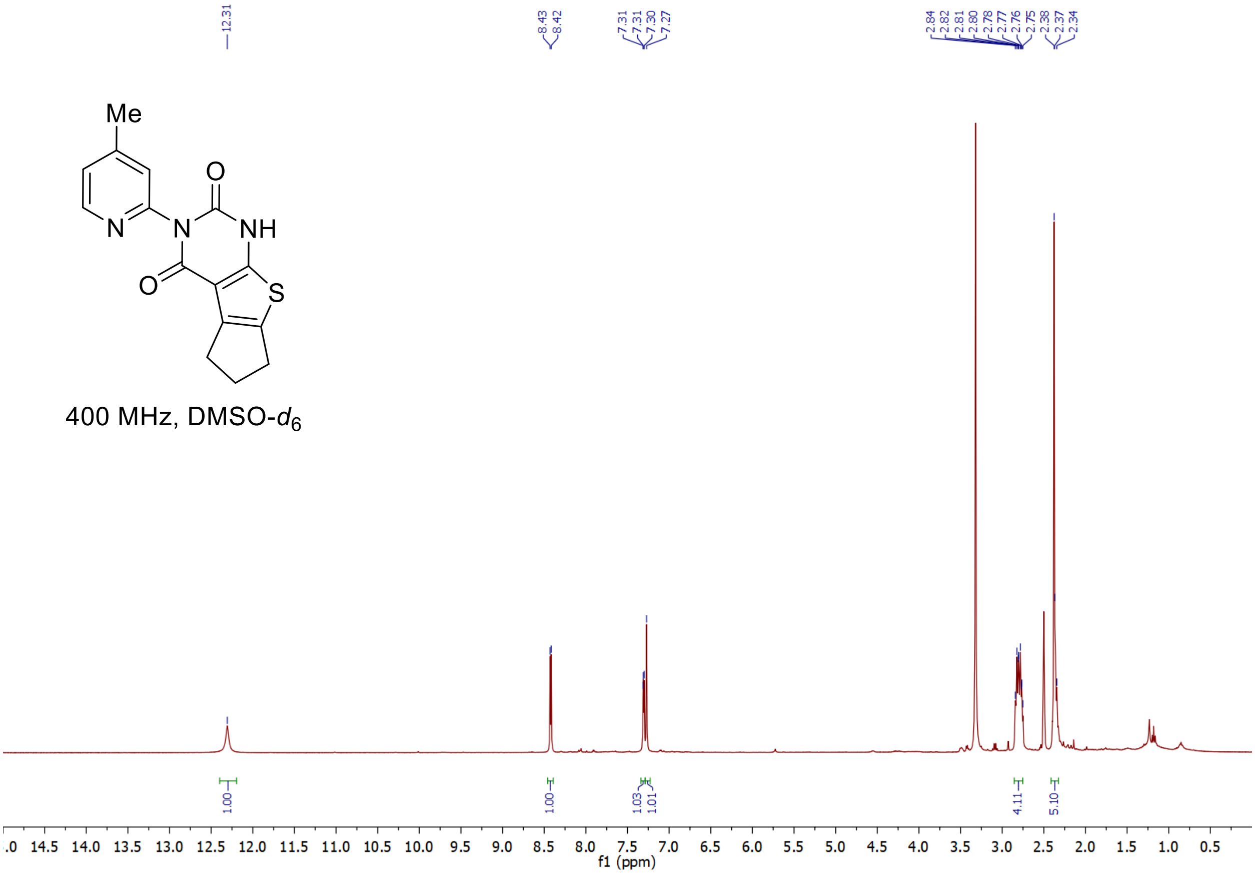


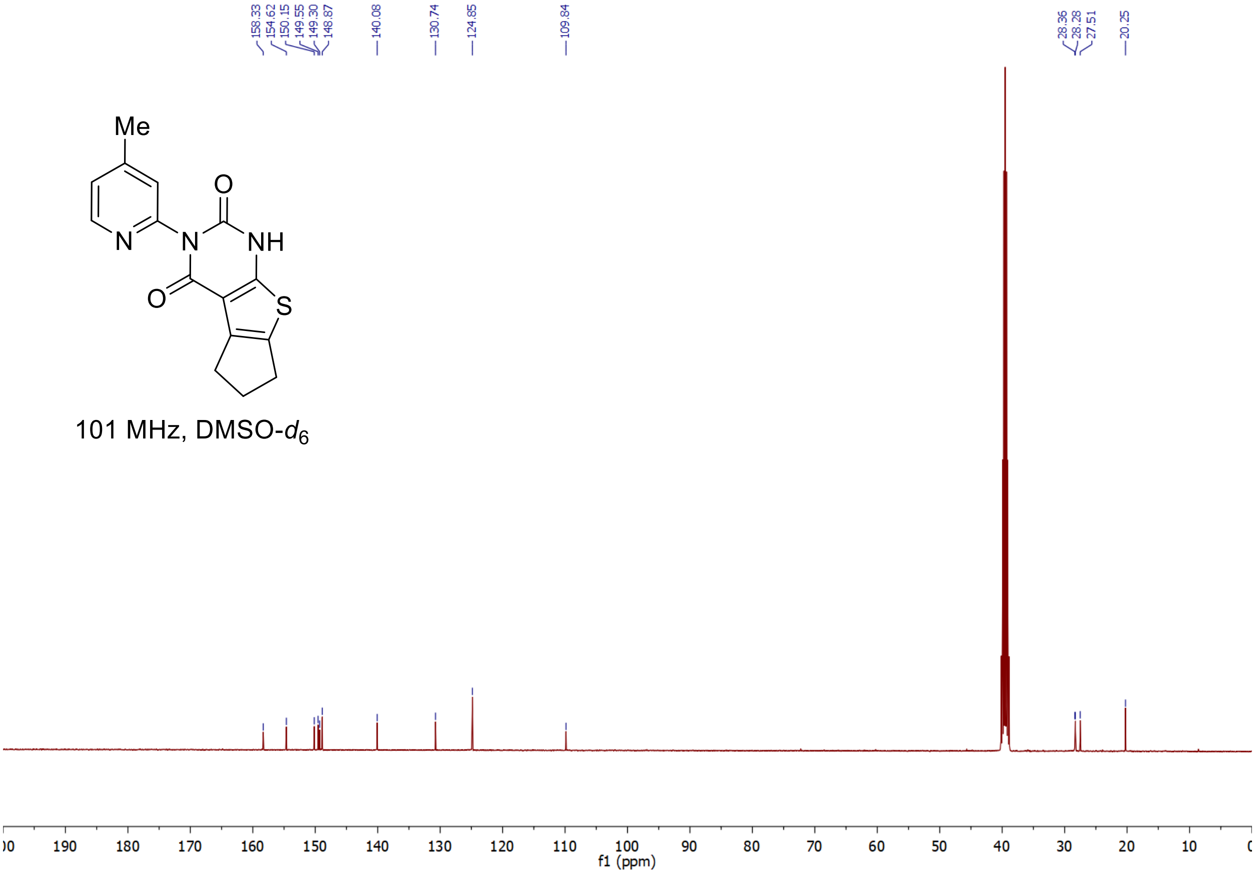

**Table S1 Crystal data and structure refinement for 3a.**

| | |
|---|---|
| Identification code | CJL-278 |
| CCDC number | 2249286 |
| Empirical formula | $C_{14}H_{11}N_3O_2$ |
| Formula weight | 253.26 |
| Temperature/K | 100(2) |
| Crystal system | monoclinic |
| Space group | $P2_1/c$ |
| a/Å | 11.4062(3) |
| b/Å | 14.6246(3) |
| c/Å | 6.9952(2) |
| α/° | 90 |
| β/° | 94.055(2) |
| γ/° | 90 |
| Volume/Å$^3$ | 1163.96(5) |
| Z | 4 |
| $\rho_{calc}$g/cm$^3$ | 1.445 |
| μ/mm$^{-1}$ | 0.822 |
| F(000) | 528.0 |
| Crystal size/mm$^3$ | 0.24 × 0.22 × 0.16 |
| Radiation | Cu Kα (λ = 1.54184) |
| 2Θ range for data collection/° | 7.77 to 159.398 |
| Index ranges | $-14 \leq h \leq 10$, $-17 \leq k \leq 18$, $-8 \leq l \leq 8$ |
| Reflections collected | 8636 |
| Independent reflections | 2434 [$R_{int}$ = 0.0380, $R_{sigma}$ = 0.0307] |
| Data/restraints/parameters | 2434/0/173 |
| Goodness-of-fit on $F^2$ | 1.045 |
| Final R indexes [I>=2σ (I)] | $R_1$ = 0.0432, $wR_2$ = 0.1137 |
| Final R indexes [all data] | $R_1$ = 0.0469, $wR_2$ = 0.1167 |
| Largest diff. peak/hole / e Å$^{-3}$ | 0.24/–0.32 |
